# A lncRNA-encoded mitochondrial micropeptide exacerbates microglia-mediated neuroinflammation in retinal ischemia/reperfusion injury

**DOI:** 10.1038/s41419-023-05617-2

**Published:** 2023-02-15

**Authors:** Xintong Zheng, Mingwei Wang, Shuting Liu, Haiqiao Chen, Yifei Li, Fa Yuan, Ludong Yang, Suo Qiu, Hongwei Wang, Zhi Xie, Mengqing Xiang

**Affiliations:** 1 State Key Laboratory of Ophthalmology, Zhongshan Ophthalmic Center, Sun Yat-sen University, Guangdong Provincial Key Laboratory of Ophthalmology and Visual Science, Guangzhou, 510060 China; 2 Guangdong Provincial Key Laboratory of Brain Function and Disease, Zhongshan School of Medicine, Sun Yat-sen University, Guangzhou, 510080 China

**Keywords:** NOD-like receptors, Neuroimmunology

## Abstract

As a common pathology of many ocular disorders such as diabetic retinopathy and glaucoma, retinal ischemia/reperfusion (IR) triggers inflammation and microglia activation that lead to irreversible retinal damage. The detailed molecular mechanism underlying retinal IR injury, however, remains poorly understood at present. Here we report the bioinformatic identification of a lncRNA *1810058I24Rik* (*181-Rik*) that was shown to encode a mitochondrion-located micropeptide Stmp1. Its deficiency in mice protected retinal ganglion cells from retinal IR injury by attenuating the activation of microglia and the Nlrp3 inflammasome pathway. Moreover, its genetic knockout in mice or knockdown in primary microglia promoted mitochondrial fusion, impaired mitochondrial membrane potential, and reactive oxygen species (ROS) production, diminished aerobic glycolysis, and ameliorated inflammation. It appears that *181-Rik* may trigger the Nlrp3 inflammasome activation by controlling mitochondrial functions through inhibiting expression of the metabolic sensor uncoupling protein 2 (Ucp2) and activating expression of the Ca^2+^ sensors S100a8/a9. Together, our findings shed new light on the molecular pathogenesis of retinal IR injury and may provide a fresh therapeutic target for IR-associated neurodegenerative diseases.

## Introduction

Ischemia/reperfusion (IR) is a pathological state caused by a restriction of blood supply to an organ followed by blood supply restoration accompanied by reoxygenation. It leads to metabolic imbalance, production of reactive oxygen species (ROS), pro-inflammatory response, severe tissue hypoxia, and microvascular dysfunction [1]. The reperfusion process in particular enhances the activation of innate and adaptive immune responses and cell death programs [1]. Retinal IR injury is a common cause of many ocular diseases such as diabetic retinopathy, glaucoma, retinal artery/vein occlusion, and so on. These disorders often lead to visual impairment or even blindness [2]. For instance, the increased intraocular pressure in glaucoma compresses the fundus blood vessels, resulting in blood flow imbalance, loss of oxygen and nutrition, and damage to retinal ganglion cells (RGCs); Following pressure release, blood perfusion exacerbates RGC degeneration through toxic glutamate invasion and ROS production, eventually leading to irreversible RGC damage and death [3]. Therefore, the study of the mechanism of retinal IR-mediated RGC injury is of great importance to the prevention and treatment of glaucoma.

Microglia are the resident immune cells of the central nervous system (CNS). As the most abundant immune cells in the retina, microglia are usually distributed in three locations: the ganglion cell layer (GCL), inner plexiform layer and outer plexiform layer [4, 5]. The functions of microglia include immune monitoring, regulating immune response, phagocytosis, synaptic pruning, maintaining synaptic structure and function, and regulating neurogenesis and axonal growth [5]. Upon tissue damage, microglia become activated, proliferate and migrate to the lesion site in a short time [6]. When activated, they change their morphology and gene expression, phagocytose dying cell debris and release a wide range of pro- or anti-inflammatory mediators [6–8]. In neurodegenerative retinal diseases such as glaucoma, neuroinflammation occurs immediately after IR injury and plays an important role in the pathological process, which is associated with the release of pro-inflammatory cytokines [9–12]. The production of cytokines by microglia is controlled by inflammasome activation [13, 14], which is marked by the oligomerization of the Asc protein into a complex containing Nlrp protein sensors and Casp1 (Caspase-1) [15]. Upon the formation of the protein complex, the inflammasome activates Casp1, which proteolytically activates the pro-inflammatory cytokines IL-1β and IL-18 as well as the pyroptosis effector protein Gsdmd (Gasdermin). Thus, this process is cytotoxic and causes a pro-inflammatory form of cell death called pyroptosis [15].

Pyroptosis plays an important role in RGC death induced by retinal IR injury [16–18]. It has been demonstrated that *Gsdmd* disruption in mice results in a significant decrease in RGC death in IR-treated retinas [18]. This is because retinal IR activates Casp8, which promotes NF-κB nuclear translocation and drives HIF1α signaling. HIF1α in turn promotes the formation of downstream Nlrp3/Nlrp12/Nlrc4 inflammasomes, leading to the activation of Casp1 [18]. Activated Casp1 not only proteolytically activates the pro-inflammatory cytokine IL-1β but also cleaves Gsdmd into the active, N-terminal Gsdmd that causes microglial pyroptosis and induces RGC death [18]. In addition, the lncRNA H19 has been shown to mediate RGC death during retinal IR injury by forming a competitive endogenous RNA network (ceRNET) with miR-21 and Pdcd4 (programmed cell death factor 4) to facilitate *Pdcd4* gene expression, which leads to decreased mitochondrial membrane potential, overproduction of ROS, imbalance of NLRP3/6 inflammasomes, pyroptosis, and release of IL-1β and IL-18 [17].

Mitochondria can be considered as a central hub of the immune system to control the activation, survival, differentiation, and transcription of immune cells by affecting their own dynamics, metabolism, ROS production, mitophagy, and so on [19–22]. They exist in a dynamic state of constant fission and fusion. Fission is the division of one mitochondrion into two daughter mitochondria, whereas fusion is a union of two mitochondria into one mitochondrion. Dynamic transitions are vital for diverse mitochondrial functions and cellular needs [23]. For example, deficiency of the mitochondrion-located protein Ucp2 upregulates the activities of macrophages while increasing immune response in mice [24–26]. It alters mitochondrial dynamics, diminishes mitochondrial membrane potential (MMP), inhibits ROS production, and modulates metabolism [26–29]. Interestingly, mitochondria are rich in a large number of micropeptides [<100 amino acids (AAs)], many of which are encoded by lncRNAs or circRNAs [30]. Previous studies have shown that mitochondrial micropeptides may regulate mitochondrial metabolism, oxidative phosphorylation (OXPHOS), respiratory complex assembly, ROS production, and other functions by binding to larger proteins [30–33]. For instance, Mtln promotes the exercise capacity and muscle formation in mice by regulating mitochondrial complex assembly and fatty acid β-oxidation [34–36]; Uqcc3 not only affects the assembly of respiratory complex III (CIII) and ATP production, but also participates in the maintenance of cristae and the composition of cardiolipin [37–39]. Patients with a *UQCC3* mutation display serious symptoms such as lactic acidosis, hypoglycemia, and developmental retardation [37].

Given the crucial roles of non-coding RNA-encoded micropeptides in regulating mitochondrial and metabolic functions, it is possible but remains to be determined whether lncRNA-encoded micropeptides participate in and regulate retinal IR-induced inflammatory responses. In this study, through transcriptome profiling of mouse retinas, we identified a lncRNA *1810058I24Rik* (*181-Rik*) that encodes a mitochondrial micropeptide homologous to zebrafish Stmp1. Mouse Stmp1 is widely distributed in the retina and located in both the inner and outer membranes of mitochondria. *181-Rik* deficiency in mice was able to enhance RGC survival and inhibit the activation of microglia and inflammasomes following retinal IR injury. Its knockout or knockdown resulted in mitochondrial dynamic changes, decreased mitochondrial membrane potential (MMP) and ROS production, unbalanced metabolism in glucose, fatty acid, and glutamine oxidation, and diminished inflammatory responses, indicating that *181-Rik*-encoded Stmp1 may regulate retinal IR-induced RGC death by modulating microglial inflammatory response through influencing mitochondrial functions.

## Methods

### Mice

All experiments on mice were performed according to the IACUC (Institutional Animal Care and Use Committee) standards, and approved by Zhongshan Ophthalmic Center and Sun Yat-sen University. The *1810058I24Rik* (*181-Rik*) knockout (KO) mice were generated using the CRISPR/Cas9 gene editing technology by Shanghai Model Organisms Center, Inc. (Shanghai, China). In KO mice, most of exon 2 and the entire exon 3 of *181-Rik* were substituted by the GFP sequence. The C57BL6 mice were purchased from the Vital River Laboratories (Beijing, China). All genotypes were determined by PCR using the following primers (from 5′ to 3′): *181-Rik* wild-type allele: AATAACCCTGGGCAAACCGCTCTA and TTCCCAAACCGTCTTCCTCCTA; KO allele: GGGGATGAGACTTTTGCTGGGTAG and GGTCTTGTAGTTGCCGTCGTCCTT.

### Establishment of retinal IR mouse models

The retinal ischemia/reperfusion (IR) mouse models were established as described [13, 17]. In brief, after anesthetization and mydriasis, a 30 G needle that contained normal saline (NS) was cannulated into the mouse anterior chamber. The height of NS was about 1.1 m, which would result in ~80 mmHg in intraocular pressure (IOP). After 60 min, the IOP was returned to normal level by carefully withdrawing the needle. Tobramycin was then administered to prevent bacterial infection.

### Immunohistochemistry, MitoTracker labeling, and TUNEL Labeling

Immunohistochemistry was carried out as described previously [40]. For section labeling, tissues were fixed in 4% paraformaldehyde (PFA) in PBS for 1 h at 4 °C and sectioned at 14–20 μm. For retinal wholemount labeling, mice were perfused with PBS followed by 4% PFA. The retinas were then harvested and fixed in 4% PFA for 1 hr. For cell labeling, cells grown on matrigel-precoated glass coverslips were fixed in 4% PFA for 15 min at room temperature (RT).

Samples were washed three times with 0.1% Triton-X in PBS (PBST) for 5 min each before being incubated in 5% normal donkey serum in PBST for 1 h at RT. The primary antibodies in 5% normal donkey serum in PBST were added for overnight (for tissue sections and cells) or 48 h (for retinal wholemount) incubation at 4 °C. After washing with PBST, secondary antibodies with DAPI were added and incubated for 1 h at RT. Images were captured by a laser scanning confocal microscope (Carl Zeiss, LSM700). Primary and secondary antibodies for immunostaining are listed in Tables S4 and S6, respectively.

MitoTracker labeling was performed using MitoTracker Red CMXRos following the manufacturer’s protocol (Thermo Fisher Scientific). Cell samples were prepared as described above, labeled with MitoTracker and counterstained with DAPI.

TUNEL labeling was performed using the In Situ Cell Death Detection Kit following the manufacturer’s protocol (Roche Diagnostics). Retinal sections were prepared as described above, TUNEL-labeled and counterstained with DAPI, and the number of TUNEL^+^ cells in the GCL was counted.

### Generation of Stmp1 antibody

The non-commercial Stmp1 antibody was produced by Abmart Shanghai Co., Ltd (Shanghai, China). The Stmp1 expression plasmid was constructed in the PET15b-Hsp70 vector to produce the antigen, which was then used to immunize New Zealand rabbits to generate polyclonal antisera. The antisera were purified by protein A/G beads to isolate IgG.

### In-situ hybridization and RNAscope

RNA in situ hybridization was carried out using a protocol described by Jensen and Wallace [41]. Retinal sections were fixed overnight in 4% PFA in PBS at 4 °C and sectioned at 14–20 μm. Digoxigenin-labeled probes were prepared following the manufacturer’s protocol (Roche Diagnostics). In brief, a *181-Rik* DNA fragment was amplified by PCR from the mouse retinal cDNA using the following primers (from 5′ to 3′): CCGGAATTCTAGACAGTGAGTCCCCAGC and CGCGGATCCGCAGTGATAACAGGAGCTT. It was then subcloned into the pBluescript vector from which the *181-Rik* probes were transcribed.

RNAscope was performed following the manufacturer’s protocol (RNAscope^®^ Multiplex Fluorescent v2). The *181-Rik* probe was obtained from Advanced Cell Diagnostics (ACD).

### Electroretinogram (ERG)

ERG recordings were carried out as previously described [42] for 3 or 4 WT and *181-Rik* KO mice (2-month-old) using the Celeris D430 rodent ERG testing system (Diagnosys LLC, MA, USA). All animals were dark-adapted overnight before experiments and recorded under the same settings and conditions. All experiments were repeated more than three times.

### Hematoxylin-eosin (HE) staining and thickness measurement

Eyeballs were fixed overnight in FAS Eyeball Fixator (Servicebio, Wuhan, China) and sectioned in paraffin at 15 μm followed by HE staining. Sectioning and staining were carried out by Servicebio. Retinal thickness was measured from GCL to the edge of the outer nuclear layer using the ImageJ software.

### Real-time quantitative reverse transcription (qRT)-PCR

The total RNA of retinas or cells was isolated using the TRIzol reagent (Invitrogen). RNA from each sample was converted to cDNA using the HiScript III 1st Strand cDNA Synthesis Kit (Vazyme, Nanjing, China). qRT-PCR was then performed using the Taq Pro Universal SYBR qPCR Master Mix (Vazyme) in the Light Cycler^®^ 384 Real-Time PCR system (Roche Diagnostics). The RNA level of each gene was normalized to that of a β-actin gene in the same sample and relative RNA quantities were obtained using the comparative cycle threshold method (∆∆CT method). All used qRT-PCR primers are listed in Table S3.

### Western blotting

Total protein was isolated from retinas or cultured cells using the RIPA buffer (Beyotime, Shanghai, China) and quantified using the BCA kit (Beyotime). Samples were loaded on gradient gels (Beyotime). The expression levels of target proteins were quantified by ImageJ and normalized to β-tubulin in the same sample. Primary and secondary antibodies for Western blotting were listed in Tables S5 and S6, respectively. Full-length Western blots are provided in the Supplemental Material.

Digitonin (Sigma) extraction of mitochondrial membrane proteins was performed as previously described [30]. In brief, we overexpressed Stmp1-Flag in mouse NIH3T3 cells and then isolated fresh mitochondria using the Mitochondria Isolation Kit (Abcam). After incubation with serial concentrations of Digitonin, the solution was centrifuged at 20,000×*g* for 20 min. The supernatant and pellet were then loaded for Western blotting analysis.

### Microglial morphology analysis

Microglial morphology analysis was carried out as described by Young and Morrison [43]. Original images of Iba1-immunolabeled retinal wholemounts were analyzed using the ImageJ software. After contrast modification, gray processing, background noise cancellation, and binarization (Fig. S7D–G), we obtained clear sketches of microglial processes. The ensuing skeletonization transformed microglial processes into absolute skeletons. The number of endpoints and total branch length of skeletons from each cell were measured and calculated. All data points were averaged from at least 20 cells.

### Cell culture and isolation of primary microglia

The HEK293T, BV2, and NIH3T3 cells were purchased from iCell Bioscience (Shanghai, China). These cells were tested for mycoplasma contamination before experiments and they were all negative. These cell lines and primary microglia were all expanded in the following culture medium containing: Dulbecco’s modified Eagle’s medium (DMEM, Hyclone), 10% fetal bovine serum (FBS) (Gibco), 1x Pen/Strep (Gibco), and 1x MEM non-essential amino acids (Gibco). The cell incubator provided an environment of 5% CO_2_ and 37 °C.

Cerebral microglia were isolated from P0-P2 mice as previously described [44]. The forebrain was dissected out of the pia mater and then digested. The mixed cell suspension was incubated in the above-mentioned medium in 5% CO_2_ at 37 °C. After 14 days, the suspending cells (microglia) were rolled down and incubated for further experiments.

### Plasmid construction, cell transfection, and lentiviral preparation and infection

The *181-Rik* ORF sequence was fused in frame with the Flag tag sequence and subcloned into the MCS (multiple cloning site) of the pcDNA3.1 expression vector (Yingrun Biotechnology, Changsha, China) which contains a CMV promoter. Plasmid DNA was transfected into HEK293T/NIH3T3 cells using the Liposomal Transfection Reagent (Yeasen Biotechnology, Shanghai, China) for about 24 h. After medium replacement, cells were cultured for another 24 h for further assays. To prepare lentiviral constructs, the Stmp1 sequence was synthesized by Genewiz and subcloned into the MCS of the lentiviral vector pLVX-MCS-3Flag-Puro (Testobio, Ningbo, China). The *181-Rik* and *S100a8* shRNAs were designed using the BLOCK-iT™ RNAi Designer tool (Thermo Fisher Scientific), synthesized by Genewiz, and then subcloned into the MCS of the lentiviral vector pLKO.1-TRC (Addgene #10878) which contains a U6 promoter. Their sequences are listed in Table S2.

To prepare lentiviruses, the pLVX-MCS-3Flag-Puro or pLKO.1-TRC viral constructs and package plasmids Pspax2 (Addgene #12260) and PMD2.G (Addgene #12259) were transfected into HEK293T cells at a DNA molar ratio of 2:1:1. After 48 h, the culture medium was harvested and ultra-centrifuged at 12,000 rpm (rotor SW28, Beckman) for 1.5 h to precipitate the lentiviruses. GFP-carrying lentiviruses of 1*81-Rik* shRNA or scramble shRNA using the vector pSLenti-U6-shRNA-CMV-EGFP-F2A-Puro-WPRE were purchased from OBiO Technology (Shanghai, China).

For lentiviral infection, the desired lentiviruses were carefully added to cultured cells. Twenty-four hrs later, the medium was changed to fresh medium containing puromycin dihydrochloride (Thermo Fisher Scientific) at 5 μg/ml to screen successfully infected cells. After 24–48 h, cells were harvested for further assays.

### Cell proliferation/viability assays

Cell proliferation/viability was measured using the CCK-8 (Cell Counting Kit-8) and MTT (methyl thiazolyl tetrazolium) kits, and by EdU (5-ethynyl-2′-deoxyuridine)-pulse labeling. The CCK-8 Kit (Biosharp, Hefei, China) and MTT Kit (Solarbio, Beijing, China) were used to detect by Microplate Reader (BioTek) the light absorbance of cells seeded in a 96-well plate. EdU labeling was performed using the Click-iT™ EdU Cell Proliferation Kit (Thermo Fisher Scientific). Cells were seeded on coverslips and the fluorescence was visualized by confocal microscopy.

### OGD/R (oxygen-glucose deprivation and reperfusion) cell model establishment

The OGD/R cell model was established as described [17]. In brief, after the culture medium was replaced with glucose-free DMEM (Gibco), the cells were incubated in a low oxygen environment (5% CO_2_ and 5% O_2_ in a modular incubator chamber) for 3 h. Following this treatment, the cells were returned to a normal environment (5% CO_2_ and 95% air) and incubated with glucose-containing and serum-free medium for 12 h (Fig. 8A).

### Blue native polyacrylamide gel electrophoresis (BN-PAGE)

The BN-PAGE was performed as described [45, 46]. The mitochondria were isolated from mouse brains using the Mitochondria Isolation Kit (Abcam) and the membrane proteins were then extracted by Digitonin at 4 mg/mg mitochondrial protein. Native protein samples combined with Coomassie blue (Beyotime) were loaded in the Hepes system (Beyotime) and electrophoresis was carried out to visualize the mitochondrial respiratory complexes.

### Transmission electron microscopy (TEM)

IR-injured retinas from WT and *181-Rik* KO mice (four retinas in each group) were dissected out and fixed with TEM Fixator (Servicebio) for 1 h. Tissue sectioning and TEM were performed by Biomisp (Wuhan, China). Cellular organelles with double-membrane structures were considered mitochondria. The morphology of each mitochondrion was quantified using ImageJ following previous descriptions [47, 48].

### ROS (reactive oxygen species) and MMP (mitochondrial membrane potential) measurement

The ROS measurement was performed using DHE (dihydroethidium, Thermo Fisher Scientific). Live cells were incubated in 50 μM DHE in PBS for 30 min at 37 °C. The fluorescence was visualized by confocal microscopy and quantified by ImageJ.

The MMP measurement was carried out using the JC-1 Mitochondrial Membrane Potential Assay Kit (Abcam). The cells were seeded on a 96-well plate and the fluorescence was measured by Microplate Reader.

### Single-cell RNA sequencing (scRNA-seq) analysis

IR-treated retinas were carefully dissected and digested with papain. Dissociated cells were sorted by FACS (fluorescence-activated cell sorting) to isolate Iba1^+^ microglia using the Alexa Fluor® 488-conjugated Iba1 antibody (Cell Signaling Technology). Single-cell library construction, sequencing, Cell Ranger processing, and Seurat analysis were performed as described previously [49, 50]. The scRNA-seq data have been deposited in the NCBI Sequence Read Archive (SRA) database under accession code PRJNA907755.

### LncRNA sequence and analysis

LncRNA sequencing was carried out as described previously [51]. In brief, retinas were dissected from mice of ten stages (embryonic day13.5–9 months), the RNA was extracted from retinas using Trizol and then reversely transcribed into cDNA, which was then sequenced using Illumina Hiseq X Ten instrument in the paired-end mode. All remaining reads were mapped to the mouse reference genome (GENCODE, Release M18: GRCm38.p6) using STAR (v2.5.2) with default parameters. The raw counts from all samples were then combined and normalized together by the DESeq2 R package. The Pheatmap R package was then used for k-means clustering (k = 8) of genes whose annotation type was lncRNA (GENCODE, vM20).

### LDH measurement

The release of LDH from microglia was measured using the LDH Cytotoxicity Assay Kit (Beyotime). Microglia were seeded on a 96-well plate and the absorbance of cell supernatant was measured at 490 nm by a microplate reader.

### Complex IV (CIV) activity measurement

The mitochondrial CIV activity measurement was carried out using the Complex IV Rodent Enzyme Activity Microplate Assay Kit (Abcam). Microglia were seeded on a 96-well plate and the changes of absorbance at 550 nm was monitored by a microplate reader.

### Cell fractionation

The mitochondria of HEK293T cells were isolated using the Mitochondria Isolation Kit for Cultured Cells (Abcam), and the cytoplasm and nucleus of HEK293T cells were fractionated using the Nuclear and Cytoplasmic Protein Extraction Kit (Beyotime).

### Quantification and statistical analysis

Each data point of cell quantification in sections or retinal wholemounts was averaged from at least three fields. To compare the fluorescent intensity, images were taken using the same parameters. Fluorescent intensity was measured by the ImageJ software using auto threshold selection. Statistical analysis was performed using GraphPad Prism 9 and Microsoft Excel Software. All the results are presented as mean ± SD for experiments conducted at least in triplicates. Unpaired or paired two-tailed Student’s *t*-test was used to assess differences between two groups, and one-way ANOVA was used to assess differences between three or more groups. A value of *p* < 0.05 is considered statistically significant.

## Results

### Identification of a conserved and developmentally regulated lncRNA 1810058I24Rik in the retina

To investigate temporal changes of transcriptomes during retinal development, we previously carried out systematic bulk RNA-seq analyses of mouse retinas from embryos to adults including stages E13.5, E15.5, E18.5, P0, P6, P13, P21, 6-week, 5-month, and 9-month [51]. In this dataset, 3641 lncRNAs are found to be expressed at various stages of retinal development and the expression heatmap shows that they are distributed in 7 clusters with distinct expression patterns (Fig. S1A, B). For instance, the lncRNAs in cluster c2 are expressed at low levels embryonically but gradually become highly expressed postnatally; by contrast, those in c5 are highly expressed in early embryonic stages but then at low levels thereafter, while those in c7 are expressed at high levels only at early postnatal stages (Fig. S1B). We noticed that one of the lncRNAs clustered in c5, *1810058I24Rik* (*181-Rik*), contains a putative small open reading frame (ORF) of 141 bp that is highly conserved in human, rat, chicken, and zebrafish, suggesting that it may have a retinal function. Further analysis revealed that *181-Rik* expression is positively correlated with 1068 protein-coding genes differentially expressed during retinal development while negatively correlated with 617 ones (Fig. S1C, D). These correlated genes are enriched for Gene Ontology (GO) terms in three major categories: (1) cell cycles such as mitotic nuclear division and sister chromatid segregation; (2) mitochondrial function including regulation of membrane potential, regulation of ion transmembrane transport, and organelle fission, and (3) visual perception such as detection of light stimulus and phototransduction (Fig. S1E).

### *181-Rik* is widely expressed in the retina and encodes a mitochondrion-located micropeptide Stmp1

**Fig. 1:**
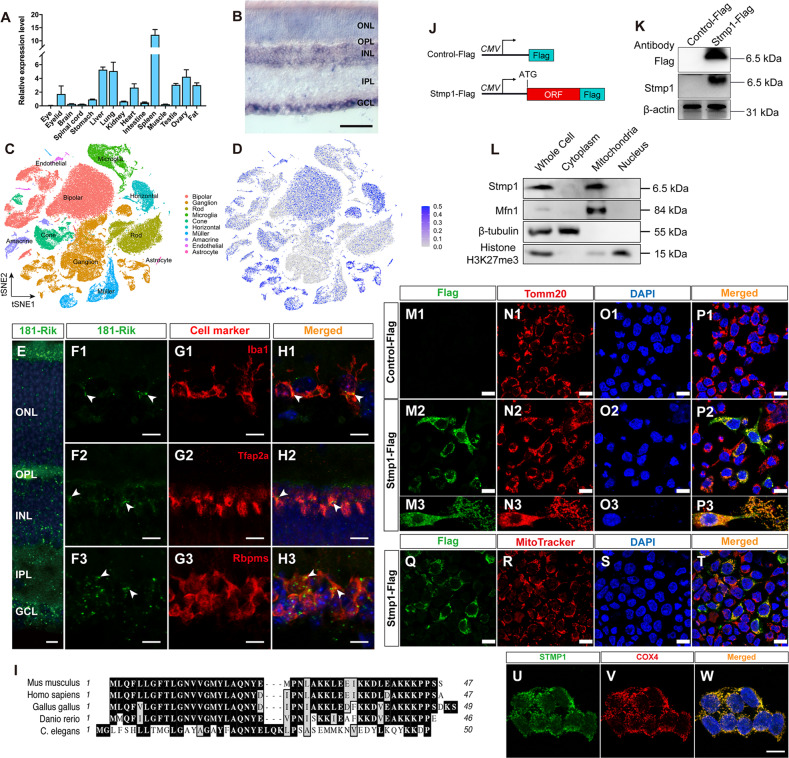
RNA and protein expression patterns of *181-Rik*. **A** Relative RNA expression levels of *181-Rik* in various murine tissues were determined by qRT-PCR assay. Data were presented as mean ± SD (*n* = 3). **B** Adult mouse retinal section in situ hybridized with a *181-Rik* probe. Scale bar: 50 μm. **C**, **D** scRNA-seq analysis of adult mouse retinal cells indicates a widespread expression of *181-Rik* in nearly all retinal cell classes. **E**, **F1**–**H1**, **F2**–**H2**, **F3**–**H3** Fluorescent RNAscope assay of *181-Rik* on adult mouse retinal sections (**E**) and in combination with immunolabeling with antibodies against Iba1, Tfap2a, and Rbpms (**F1**–**H1**, **F2**–**H2**, **F3**–**H3**), which are markers for microglia, amacrine cells, and ganglion cells, respectively. All sections were also counterstained with nuclear DAPI (blue). Scale bar: 15 μm. **I** Alignment of putative Stmp1 amino acid (AA) sequences from the indicated species. Numbers indicate AA length. **J** Schematic of tagged Stmp1 (Stmp1-Flag) and control (Control-Flag) expression constructs driven by the CMV promoter. The putative *181-Rik* ORF containing the ATG start codon is fused with a Flag tag sequence. **K** Western blotting analysis of total proteins from HEK293T cells transfected with the Control-Flag or Stmp1-Flag construct. Both the Flag antibody and a non-commercial Stmp1 antibody detected the expression of the exogenous Stmp1-Flag protein. β-actin served as the internal protein control. **L** Western blotting analysis of proteins from the whole cell, cytoplasm, mitochondria, and nucleus of HEK293T cells transfected with the Stmp1-Flag construct using the indicated antibodies. **M1**–**P1**, **M2**–**P2**, **M3**–**P3**, **Q**–**T** Immunostaining of HEK293T cells transfected with the Control-Flag or Stmp1-Flag construct with anti-Flag and anti-Tomm20 or MitoTracker. Flag and Tomm20 or MitoTracker were co-immunolabeled in mitochondria. Scale bars: 20 μm. **U**–**W** Double-immunostaining of untransfected HEK293T cells with a commercial Stmp1 antibody and an anti-COX4 antibody, along with counterlabeling by nuclear DAPI (blue). STMP1 was colocalized with COX4 in mitochondria. Scale bar: 25 μm. GCL ganglion cell layer, INL inner nuclear layer, IPL inner plexiform layer, ONL outer nuclear layer, OPL outer plexiform layer.

To gauge the possible retinal function of *181-Rik* gene, we identified retinal cell types that express its RNA transcripts. By qRT-PCR assay, *181-Rik* RNA appears to be widely expressed in many different tissues including the eye (Fig. 1A). Analysis by RNA in situ hybridization further showed that *181-Rik* signals were distributed in all major cellular layers of the mouse retina including the ganglion cell layer (GCL), inner nuclear layer and outer nuclear layer (Fig. 1B). Indeed, in a scRNA-seq dataset of single mouse retinal cells, 1*81-Rik* expression is seen at various levels in nearly all major retinal cell types such as cones, bipolar cells, amacrine cells, subsets of retinal ganglion cells (RGCs), and microglia (Fig. 1C, D). In agreement, RNAscope assay confirmed this result and demonstrated the localization of *181-Rik* in situ hybridization signals in Iba1^+^ microglia, Tfap2a^+^ amacrine cells, and Rbpms^+^ RGCs (Fig. 1E, F1–H1, F2–H2, F3–H3).

Despite being previously classified as a lncRNA, the *181-Rik* transcript was predicted to contain a putative small ORF encoding a 47-AA micropeptide that is conserved across distant species from human to nematode; in particular, it is highly homologous to the zebrafish Stmp1 short peptide which was speculated to be a subunit of the mitochondrial respiratory complexes [52] (Fig. 1I). Therefore, we assumed *181-Rik* gene would encode the mouse homolog of zebrafish Stmp1 protein. To confirm that *181-Rik* indeed encodes Stmp1 located in mitochondria, we fused the putative ORF to the Flag tag in a cDNA expression construct, and confirmed the expression of the Stmp1-Flag peptide in HEK293T cells by Western blotting with an anti-Flag antibody or a non-commercial anti-Stmp1 antibody (Fig. 1J, K). Cell fractionation combined with Western blotting further showed that Stmp1 was enriched in the mitochondrial fraction (Fig. 1L). Moreover, by immunofluorescence, Stmp1-Flag was shown to colocalize with the mitochondrial marker Tomm20 and MitoTracker probe (Fig. 1M1–P1, M2–P2, M3–P3, Q–T). Aside from exogenous Stmp1, we further verified the presence of endogenous STMP1 and its colocalization with the mitochondrial marker protein COX4 in HEK293T cells by immunolabeling with a commercial Stmp1 antibody (Fig. 1U–W), confirming the existence of mitochondrion-located Stmp1.

Consistent with the evidence that *181-Rik* encodes a mitochondrial micropeptide, existing RNA-seq and ribosomal profiling datasets clearly show the capacity of transcription and translation from this gene locus (Fig. S2A). To determine the suborganelle location of Stmp1, we extracted mitochondrial proteins from Stmp1-Flag-transfected NIH3T3 cells using a series of concentrations of Digitonin. The Stmp1-Flag peptide was shown by Western blotting to be co-extracted with proteins located in either inner or outer mitochondrial membranes (IMM or OMM) (Fig. S2B), indicating Stmp1 localization in both IMM and OMM. The mitochondrial location of Stmp1 is consistent with the observation that the genes whose expression is correlated with *181-Rik* are enriched for mitochondrial function (Fig. S1E). As well, our results agree well with a recent report which has provided evidence that mouse Stmp1, designated as Mm47 therein, is expressed in cells and located in mitochondria [53].

### *181-Rik* ablation attenuates apoptosis in retinas of IR mouse models

**Fig. 2:**
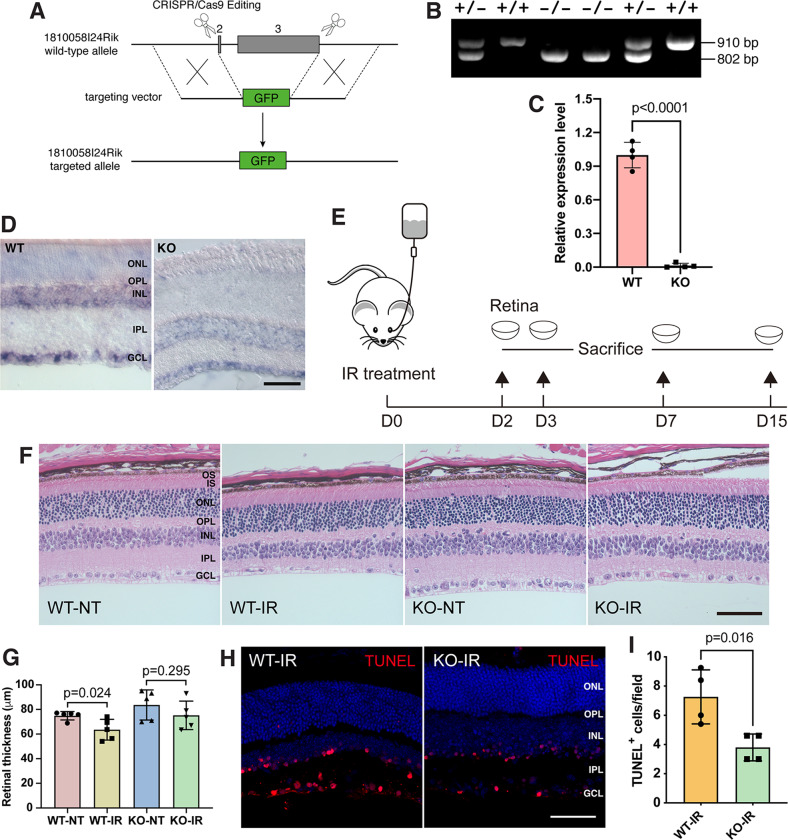
Generation of *181-Rik* knockout mice and attenuation of apoptosis by *181-Rik* deletion in IR-injured retinas. **A** Schematic diagram of targeted disruption of the *181-Rik* locus in mice. By CRISPR/Cas9 gene editing, the great majority of exon 2 and all exon 3 were substituted by the GFP ORF sequence. **B** PCR analysis of DNA from wild-type (WT), heterozygous, and homozygous (KO) mutant mice. The wild-type and targeted alleles yield a product of 910 bp and 802 bp, respectively. **C** qRT-PCR assay validated the absence of *181-Rik* RNA expression in KO mouse retinas. Data were presented as mean ± SD (*n* = 4). **D** Diminished *181-Rik* in situ hybridization signals in KO mouse retinas. Scale bar: 50 μm. **E** Schematic diagram of the establishment of ischemia and reperfusion (IR) mouse models. Acute intraocular hypertension was induced by normal saline in anesthetized mice at day 0 (D0). The retinas were harvested on days 2, 3, 7, and 15 depending on experimental requirements. **F** At 7 days post-IR injury, untreated (NT) and treated (IR) WT and KO retinal sections were stained by hematoxylin-eosin (HE). Shown are images from the peripheral region. Scale bar: 50 μm. **G** Measurements of peripheral retinal thickness. Data were presented as mean ± SD (*n* = 5). **H** At 2 days post-IR injury, cells undergoing apoptosis were TUNEL-labeled in WT and KO retinas with counterstaining by nuclear DAPI (blue). Scale bars: 50 μm. **I** Quantification of apoptotic cell death in the GCL of IR-injured WT and KO retinas. Data were presented as mean ± SD (*n* = 4). GCL ganglion cell layer, INL inner nuclear layer, IPL inner plexiform layer, IS inner segment, ONL outer nuclear layer, OPL outer plexiform layer, OS outer segment.

To examine the role of *181-Rik* in the retina, we generated knockout mice in which the reporter GFP ORF was inserted in frame at the 7th AA position of the *181-Rik* ORF and essentially replaced exons 2 and 3 (Fig. 2A, B). To confirm the substitution, we performed qRT-PCR and RNA in situ hybridization analyses and both validated a dramatic reduction of *181-Rik* RNA expression in null mutant retinas (Fig. 2C, D). However, in spite of the abundant expression of GFP transcripts, no GFP protein was detectable in mutant retinas by either Western blotting or immunostaining (Fig. S3), suggesting that the GFP fusion protein may be quite unstable. *181-Rik* inactivation did not appear to alter the morphology or cell composition of the retina. In *181-Rik*^*−/−*^ (KO) retinas, there was no significant change in the quantity of major cell types such as Chx10^+^ bipolar cells, Tfap2a^+^ amacrine cells, Rbpms^+^ RGCs, or Sox9^+^ Müller cells (Fig. S4A–F). In addition, there was no obvious size difference in the mutant optic nerve, tract, and chiasm (Fig. S4G). Accordingly, electroretinogram (ERG) recordings revealed no significant changes in amplitudes of a and b waves either in scotopic or photopic conditions in *181-Rik* KO animals (Fig. S4H–K).

Given the mitochondrial localization of the *181-Rik* product, we evaluated the effect of *181-Rik* ablation on the retinal structure and integrity under stress. We first established IR mouse models with acute intraocular hypertension and sacrificed them after 2–15 days depending on different assays (Fig. 2E). Compared to untreated (NT) retinas, in wild-type (WT) IR retinas, qRT-PCR assay revealed a significant increase in expression of inflammatory (e.g. *Casp1* and *Gsdmd*) and apoptotic (e.g., *Casp3* and *7*) genes (Fig. S5A). Additionally, both microglia and astrocytes were activated as indicated by increased Iba1 and Casp1, and Gfap immunoreactivity, respectively, while Rbpms^+^ RGCs were decreased (Fig. S5B–E, B′–E′).

By hematoxylin-eosin (HE) staining, we measured and compared the retinal thickness of *181-Rik* WT and KO retinas at NT and IR conditions (Fig. 2E). While retinas of the central region exhibited no detectable difference in thickness among the four groups (Fig. S6), IR treatment caused a significant decrease in thickness of the peripheral retina of WT mice but no change in that of KO animals (Fig. 2F, G). Consistent with this, in KO retinas at 2 days post-IR treatment, we observed an approximately twofold reduction of TUNEL-labeled apoptotic cells in the GCL compared to that in WT retinas (Fig. 2H, I), suggesting a possible neuroprotective effect of *181-Rik* deletion.

### *181-Rik* ablation enhances RGC survival in IR-injured retinas

**Fig. 3:**
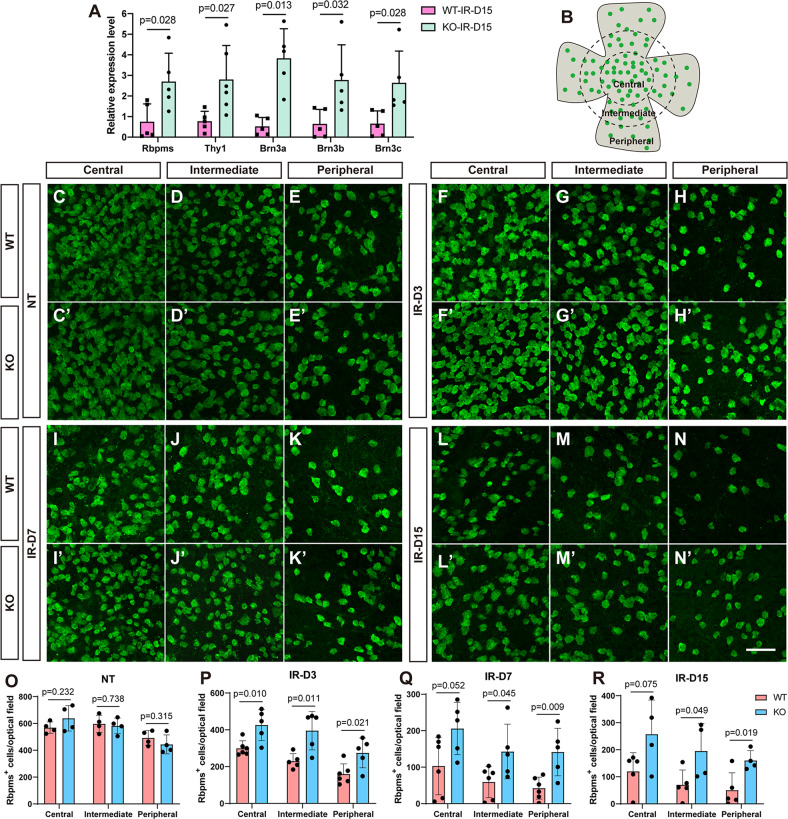
Improved retinal ganglion cell (RGC) survival in IR-injured retinas in the absence of *181-Rik*. **A** At 15 days (D15) post-IR injury, a qRT-PCR assay of RNA expression of the indicated RGC marker genes in WT and KO retinas. Data were presented as mean ± SD (*n* = 5 or 6). **B** Schematic diagram indicating that the retinal wholemount was divided into central, intermediate, and peripheral regions for more accurate quantification. **C**–**N**, **C**′–**N**′ Immunostaining of WT and KO retinal wholemounts from untreated mice (NT) and mice at 3, 7, or 15 days post-IR injury (IR-D3, 7, or 15), with an anti-Rbpms antibody. Scale bar: 100 μm. **O**–**R** Quantification of Rbpms^+^ RGCs in the three regions of NT, IR-D3, IR-D7, and IR-D15 retinas. Data were presented as mean ± SD (*n* = 4–6).

Given the observed decrease of apoptosis in the GCL of IR-treated *181-Rik* KO retinas, we investigated RGC survival in mutant retinas following IR injury. As determined by qRT-PCR assay, RNA expression levels of RGC marker genes *Rbpms, Thy1, Brn3a, Brn3b*, and *Brn3c* were all increased by severalfold (ranging from 3–7) in KO retinas at 15 days post-IR treatment compared to WT retinas (Fig. 3A), suggesting improved RGC survival in the absence of *181-Rik*. To confirm this result, we quantified Rbpms^+^ RGCs in the central, intermediate, and peripheral regions of wholemount retinas immunolabeled with an anti-Rbpms antibody (Fig. 3B). In the absence of IR treatment, WT and KO retinas displayed no significant difference in RGC cell number in all three regions (Fig. 3C–E, C′–E′, O). However, by 3 days post-IR injury, there were ~70% more RGCs remained in the intermediate and peripheral regions of KO retinas and about 40% more RGCs remained in the central area compared to WT retinas (Fig. 3F–H, F′–H′, P). By days 7 and 15, even more RGCs survived IR injury in KO retinas compared to WT ones. We found that approximately 2–3-fold more RGCs remained in KO retinas (Fig. 3I–N, I′–N′, Q, R), indicating that *181-Rik* inactivation has a strong effect on preventing RGCs from degeneration following IR injury.

### *181-Rik* ablation inhibits inflammatory response in IR-injured retinas

**Fig. 4:**
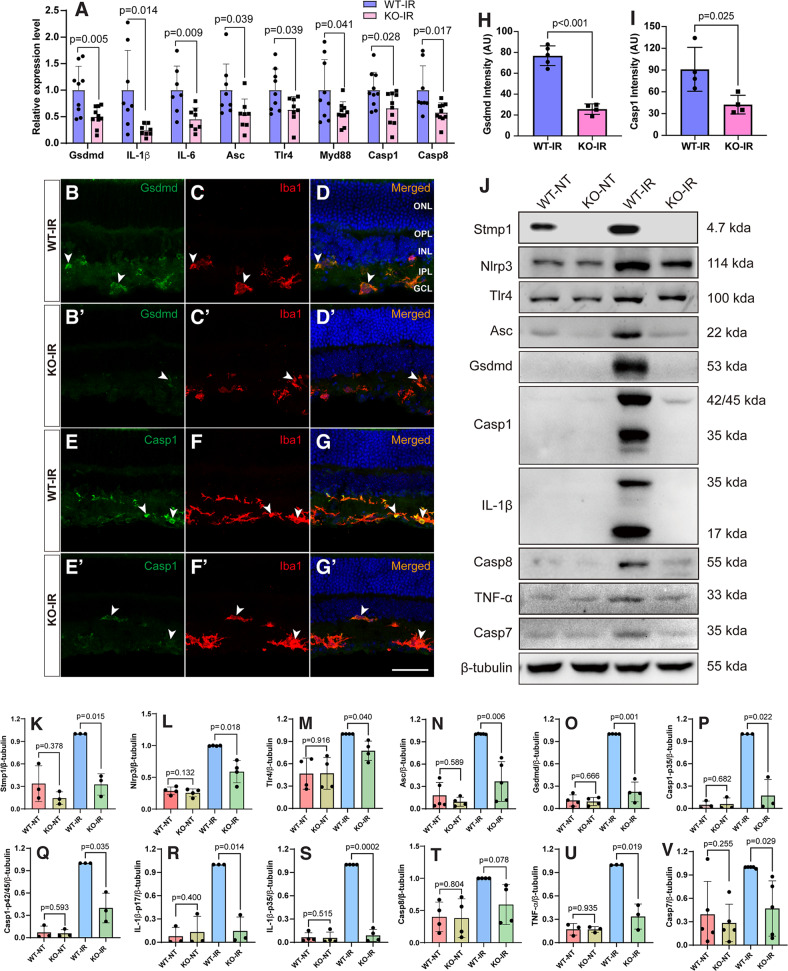
*181-Rik* inactivation impairs the Nlrp3 inflammasome pathway in IR-injured retinas. **A** At 2 days (D2) post-IR injury, a qRT-PCR assay of the indicated genes involved in the Nlrp3 inflammasome pathway in WT and KO retinas. Data were presented as mean ± SD (*n* = 8–10). **B**–**G**, **B**′–**G**′ Double-immunostaining of WT and KO retinal sections from mice at 2 days post-IR injury with antibodies against Iba1 and Gsdmd or Casp1. All sections were also counterstained with nuclear DAPI (blue). Both Gsdmd and Casp1 immunofluorescence was obviously diminished in KO retinas. Scale bar: 50 μm. **H**, **I** Quantification of Gsdmd and Casp1 immunofluorescence intensity in WT and KO retinas. Data were presented as mean ± SD (*n* = 4 or 5) in arbitrary unit (AU). **J** On day 2 post-IR injury, Western blotting analysis was performed for Stmp1, the indicated Nlrp3 inflammasome pathway-related proteins, and Casp7 expressed in untreated (NT) and treated (IR) WT and KO retinas. Compared to the NT groups, all proteins showed an upregulation in WT-IR but not in KO-IR retinas. Moreover, Casp1 and IL-1β produced cleaved proteins (35 and 17 kDa respectively) in WT-IR retinas. β-tubulin served as the internal protein control. **K**–**V** Quantification of relative protein expression levels in WT-NT, KO-NT, WT-IR, and KO-IR retinas. Data were presented as mean ± SD (*n* = 3–5).

As shown previously and above, IR causes retinal inflammation (Fig. S5) which leads to RGC degeneration [2, 9, 54]. We, therefore, asked whether *181-Rik* inactivation improves RGC survival by alleviating inflammation. At 2 days post-IR injury, qRT-PCR assay revealed that many genes involved in the Nlrp3 inflammasome pathway (e.g., *Gsdmd, IL-1β, IL-6, Asc, Tlr4, Myd88, Casp1*, and *Casp8*) were downregulated by 34–76% in KO retinas compared to WT ones (Fig. 4A), suggesting that *181-Rik* ablation suppresses inflammatory response. Consistent with this result, immunostaining for Gsdmd and Caspase-1 (Casp1) in IR retinal sections revealed their colocalization with Iba1 in microglia, and that their fluorescence intensity in KO retinas dropped markedly by about 3- and 2-fold, respectively (Fig. 4B–I, B′–G′). By Western blotting, we observed prominent upregulation of inflammasome-related proteins in WT-IR retinas compared to NT ones, including Nlrp3, Tlr4, Asc, Gsdmd, Casp1, IL-1β, and TNF-α along with apoptotic protein Caspase 7 (Casp7) (Fig. 4J–V). However, no obvious increase of these proteins was seen in KO-IR retinas, and compared to WT-IR retinas, the levels of these proteins were all significantly reduced in KO-IR retinas (Fig. 4J–V). In agreement with the elevated inflammatory response in WT-IR retinas, Stmp1 expression was greatly upregulated; but in KO retinas, it was essentially absent confirming targeted *Rik-181* deletion (Fig. 4J, K). These results thus suggest that inactivating *181-Rik* has a strong inhibitory effect on retinal inflammation caused by IR injury.

### Microglial changes caused by *181-Rik* ablation in IR-injured retinas

**Fig. 5:**
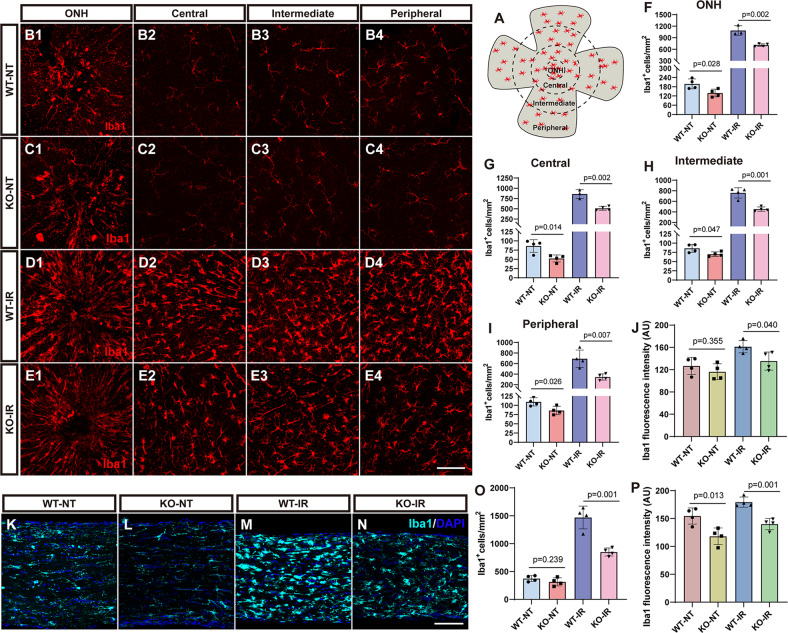
Reduced microglia in IR-injured *181-Rik* mutant retinas and optic nerves. **A** Schematic diagram indicating that the retinal wholemount was divided into four regions for more accurate quantification: optic-nerve head (ONH), central, intermediate, and peripheral. **B1**–**E1**, **B2**–**E2**, **B3**–**E3**, **B4**–**E4** Immunostaining of WT and KO retinal wholemounts from untreated mice (NT) and mice at 7 days post-IR injury, with an anti-Iba1 antibody. There were fewer microglia in KO retinas. Scale bar: 100 μm. **F**–**I** Iba1^+^ microglia density in different regions (ONH, central, intermediate and peripheral) of WT-NT, KO-NT, WT-IR, and KO-IR retinas. Data were presented as mean ± SD (*n* = 3 or 4). **J** Quantification of Iba1 immunofluorescence intensity in WT-NT, KO-NT, WT-IR, and KO-IR retinas. Data were presented as mean ± SD (*n* = 4) in arbitrary unit (AU). **K**–**N** Immunostaining of longitudinal optic nerve sections from WT-NT, KO-NT, WT-IR, and KO-IR mice with an anti-Iba1 antibody. Scale bar: 50 μm. **O** Iba1^+^ microglia density in WT-NT, KO-NT, WT-IR, and KO-IR optic nerves. Data were presented as mean ± SD (*n* = 4). **P** Quantification of Iba1 immunofluorescence intensity in WT-NT, KO-NT, WT-IR, and KO-IR optic nerves. Data were presented as mean ± SD (*n* = 4) in AU.

Since microglia are the major cell type responsible for the inflammatory response in the retina, we examined microglial changes by immunostaining with an antibody against the microglial cell marker Iba1. On retinal sections, it appeared that there were fewer microglia in KO retinas at P0 and adults independent of IR treatment (Fig. S7A–C, A′–C′). To quantify the number of microglia, we immunofluorescently labeled retinal wholemounts, which were divided into four regions for more accurate quantification: optic-nerve head (ONH), central, intermediate, and peripheral (Fig. 5A). This analysis showed that in untreated animals, there was ~20–40% decrease of microglia in all four areas of KO retinas compared to the WT ones (Fig. 5B1–B4, C1–C4, F–I). Similarly, by 7 days after IR injury, KO retinas exhibited about 35–50% reduction of microglia compared to WT retinas (Fig. 5D1–D4, E1–E4, F–I). Moreover, their Iba1 immunofluorescence was about 20% weaker than that in WT-IR retinas (Fig. 5D1–D4, E1–E4, J), consistent with the finding that Iba1 expression levels partly reflect the extent of microglial activation [55]. In longitudinal sections of the optic nerve, Iba1 labeling showed that the density of microglia was also decreased in KO mice but only if they were injured by IR (Fig. 5K–O). Similarly, KO optic nerves displayed weaker Iba1 immunofluorescence than WT controls (Fig. 5K–N, P). These results suggest that *181-Rik* ablation inhibits microglia activation in IR-injured retinas and optic nerves.

**Fig. 6:**
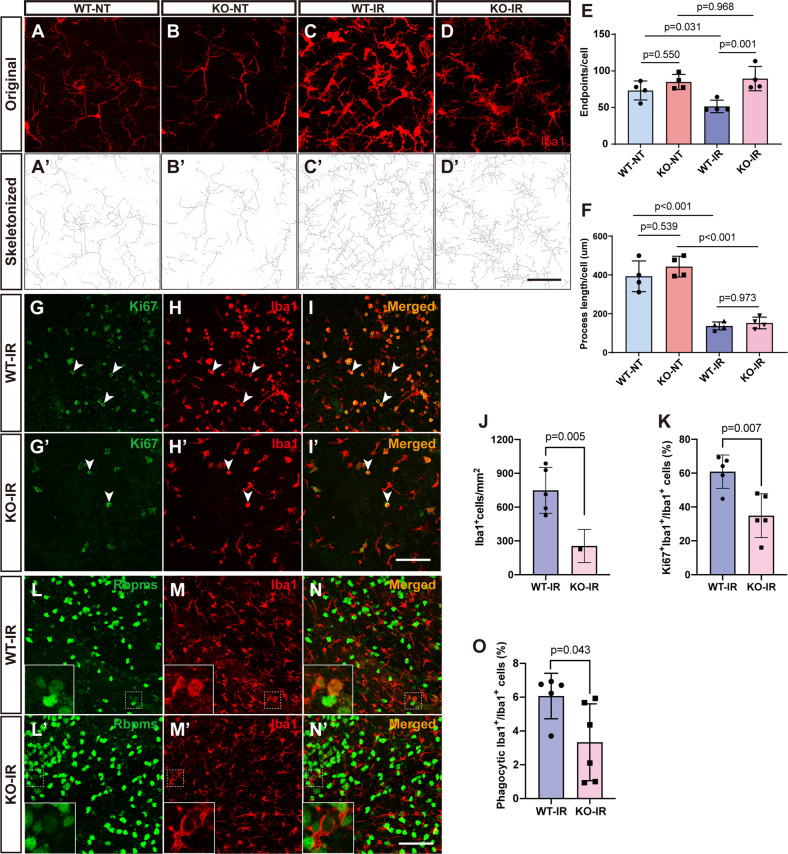
Alteration of microglial morphology, proliferation, and phagocytosis in IR-injured *181-Rik* mutant retinas. **A**–**D**, **A**′–**D**′ Original and skeletonized images of Iba1 immunolabeling in WT and KO retinal wholemounts from untreated mice (NT) and those at 7 days post-IR injury. Scale bar: 50 μm. **E**, **F** Number of endpoints and branch length of Iba1^+^ microglia in WT-NT, KO-NT, WT-IR, and KO-IR retinas. Data were presented as mean ± SD (*n* = 4). **G**–**I**, **G**′–**I**′ Double-immunostaining of WT and KO retinal wholemounts from mice at 2 days post-IR injury with antibodies against Ki67 and Iba1. Scale bar: 100 μm. **J** Microglial density in WT and KO peripheral retinas from mice at 2 days post-IR injury. Data were presented as mean ± SD (*n* = 5). **K** Proportion of proliferative microglia in WT and KO retinas at 2 days post-IR injury. Data were presented as mean ± SD (*n* = 5). **L**–**N**, **L**′–**N**′ Double-immunostaining of WT and KO retinal wholemounts from mice at 7 days post-IR injury with antibodies against Rbpms and Iba1. Arrowheads point to RGCs that were being phagocytosed by microglia and insets show corresponding outlined regions at a higher magnification. Scale bar: 100 μm. **O** Proportion of phagocytic microglia in WT and KO retinas at 7 days post-IR injury. Data were presented as mean ± SD (*n* = 5 or 6).

Because microglia undergo a typical morphologic change from the ramified toward an ameboid shape upon activation, we analyzed microglia morphology on binary and skeletonized images to evaluate microglia activation (Fig. S7D–G). For the number of endpoints and branch length, untreated WT and KO microglia in the retina were similar (Fig. 6A, B, A′, B′, E, F). By 7 days following IR injury, as expected, WT microglia had fewer endpoints and much shorter process branch length compared with WT-NT microglia, consistent with activated microglia with a stubbier morphology (Fig. 6A, C, A′, C′, E, F). However, KO-IR microglia had ~77% more endpoints per cell than WT-IR microglia although their branch length has no significant difference (Fig. 6C–F, C′, D′). This result suggests that KO microglia are more “fragmented” than activated microglia and closer to inactivated ones in shape, in agreement with the conclusion that *181-Rik* ablation has an inhibitory effect on microglia activation.

The observed reduction of microglia in KO-IR retinas may be due to decreased proliferation of microglia during the activation process. We tested this possibility by double-immunostaining of retinal wholemounts with antibodies against Iba1 and the proliferation marker Ki67. On day 2 following IR injury, the density of Iba1^+^ microglia in KO retinas was dropped by about threefold compared to that of WT retinas (Fig. 6H, H′, J). Similarly, the proportion of Ki67^+^Iba1^+^ double-positive cells was diminished by approximately twofold in KO retinas compared to WT controls (Fig. 6G–I, G′–I′, K), indicating decreased microglia proliferation in IR-injured KO retinas. This result is consistent with the finding that the genes positively correlated with *181-Rik* are enriched for cell cycle genes (Fig. S1E).

Phagocytosis is a key function of activated microglia, and after IR injury they frequently approach and engulf apoptotic neurons, especially RGCs [56]. We, therefore, examined the phagocytic activity of microglia by double-immunostaining with Rbpms and Iba1 antibodies to assess the effect of *181-Rik* inactivation. We found that by 7 days after IR injury, the proportion of phagocytic microglia, which are those wrapping dying RGCs weakly labeled for Rbpms, was decreased by nearly twofold in KO retinas compared to WT controls (Fig. 6L–O, L′–N′), consistent with the observed reduction of activated microglia and RGC death in IR-injured KO retinas.

### Microglial changes revealed by single-cell transcriptome profiling in IR-injured *181-Rik* mutant retinas

**Fig. 7:**
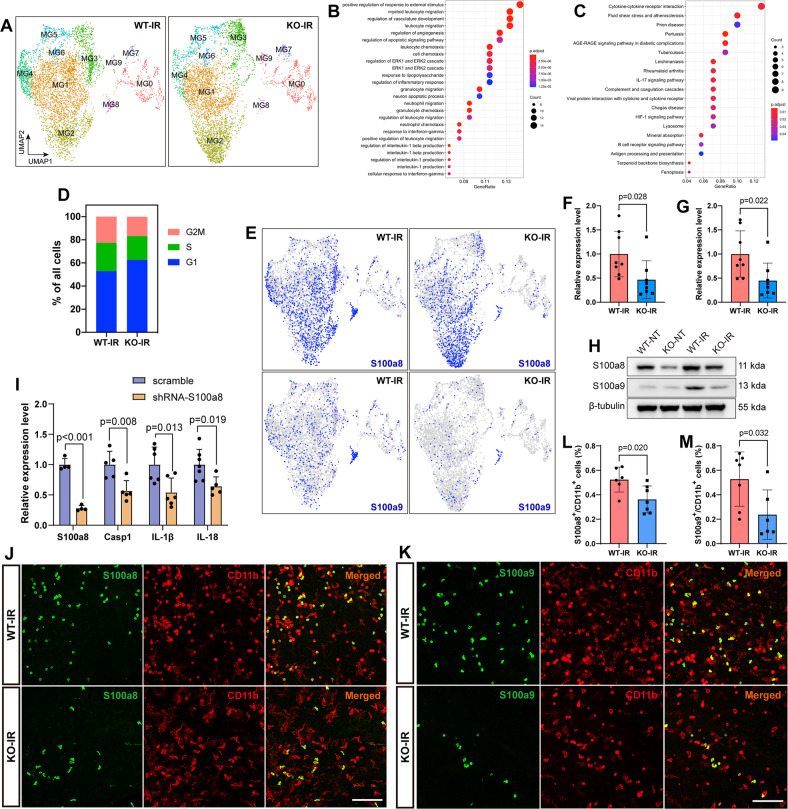
Single-cell transcriptome profiling changes and downregulation of *S100a8/9* in microglia of IR-injured *181-Rik* mutant retinas. **A** Comparison of UMAP plots of single microglial cells from WT and KO retinas at 2 days post-IR injury. The names of the ten microglial clusters are indicated. **B**, **C** Analyses of GO term enrichment (**B**) and KEGG pathway enrichment (**C**) for differentially expressed genes between WT-IR and KO-IR microglia. **D** Cell phase analysis shows a change in cell phase distribution between WT-IR and KO-IR microglia. **E** UMAP plots colored by *S100a8* or *S100a9* expression in WT-IR and KO-IR microglia. **F**, **G** qRT-PCR assays validating downregulation of *S100a8* (**F**) and *S100a9* (**G**) transcripts in KO-IR microglia. Data were presented as mean ± SD (*n* = 8–10). **H** On day 2 post-IR injury, Western blotting analysis was performed for S100a8 and S100a9 expressed in untreated (NT) and treated (IR) WT and KO retinas. These two proteins were upregulated in WT-IR but not in KO-IR retinas. β-tubulin served as the internal protein control. **I** qRT-PCR assays of *S100a8* and the indicated Nlrp3 inflammasome pathway-related genes in OGD/R-treated microglia infected with *S100a8* shRNA or scramble lentiviruses. Data were presented as mean ± SD (*n* = 4–7). **J**, **K** Double-immunostaining of WT and KO retinal wholemounts from mice at 2 days post-IR injury with antibodies against CD11b and S100a8 (**J**) or S100a9 (**K**). Scale bar: 100 μm. **L**, **M** Percentage of S100a8^+^ and S100a9^+^ microglia in WT-IR and KO-IR retinas. Data were presented as mean ± SD (*n* = 6–8).

To further explore the effect of *181-Rik* ablation on microglia, we performed single-cell transcriptome profiling of Iba1^+^ microglia which were sorted by FACS (fluorescence-activated cell sorting) from pooled WT and KO retinas on day 2 post-IR injury. A total of 10646 cells passed quality control metrics and UMAP (Uniform Manifold Approximation and Projection) visualization by Seurat [50] yielded ten unsupervised clusters MG0-MG9 (Fig. S8A). Although these clusters express common microglial markers such as Iba1, CD68, and CD11b, they have distinct transcriptional profiles (Fig. S8B, C, E, F, E′, F′). In addition, the proportions of microglia distributed in these clusters also alter in KO-IR retinas compared to WT-IR controls (Fig. 7A and S8D). We performed a differential gene expression test and found a set of genes differentially expressed in KO-IR retinas, which are enriched for GO and KEGG terms such as response to lipopolysaccharide, regulation of inflammatory response, IL-1β production, and rheumatoid arthritis (Fig. 7B, C and Table S1), in agreement with a role of *181-Rik* in inflammation. Consistent also with the observed decrease of microglia proliferation in IR-injured KO retinas, cell cycle phase analysis revealed fewer microglia in the S and G2M phases in KO-IR retinas compared to the controls (Fig. 7D), consistent with the recent finding that Stmp1 is able to promote G1/S transition and cell proliferation [57]. We further confirmed this phenomenon in the OGD/R (oxygen-glucose deprivation and reperfusion)-injured BV2 microglial cells and found that *181-Rik* knockdown significantly diminished BV2 cell proliferation and viability (Fig. S9).

The MG0 cluster represents the homeostatic microglia which are characterized by the expression of *P2ry12, Tmem119, Hexb, Gpr34, Sparc*, etc. (Fig. S8C). In agreement with the observed reduction of microglial activation in KO-IR retinas, there are more MG0 microglia as well as *Tmem119* and *P2ry12* RNA expression and Tmem119^+^ microglia in KO-IR retinas compared to the controls (Fig. 7A and S8C, G–L, G′–K′). The microglia of the MG9 cluster express a number of photoreceptor marker genes such as *Rp1, Rcvrn, Gnat1*, and *Pde6g* (Fig. S8C), resembling the previously identified photoreceptor-phagocytosing microglial type [58]. MG6 cells express high levels of *Oasl1, Rsad2* and *Ifit1-3* while MG7 microglia exhibit distinct expression of *Fads2, Cpne8, Lyve1, Bmp2*, and *Jun* (Fig. S8C).

The MG8 microglia express some neutrophil marker genes such as *Hdc, S100a8, S100a9, G0s2*, and *Retnlg* (Fig. S8C). S100a8 and S100a9 have been shown previously to promote inflammation via the Nlrp3 inflammasome pathway [59], so MG8 microglia may be considered to be pro-inflammatory. Consistent with this idea and the observed suppression of inflammatory response by *181-Rik* inactivation, the proportion of MG8 cells is dramatically reduced by about fourfold in KO-IR retinas compared to controls (Fig. 7A and S8D). Correspondingly, the fraction of S100a8- and S100a9-immunoreactive microglia was decreased by 31% and 55%, respectively (Fig. 7J–M). Indeed, *S100a8* and *S100a9* were two of the most downregulated genes in KO-IR microglia (Fig. 7E–G and Table S1), and Western blotting showed a marked decrease of both proteins in KO-IR retinas as well (Fig. 7H). To assess the effect of downregulation of *S100a* genes on inflammation, we knocked down S100a8 expression in mouse microglial cells and found that this knockdown resulted in downregulation of *Casp1, IL-1β*, and *IL-18* by 36–46% in OGD/R-injured microglia (Fig. 7I), suggesting that S100a8 and S100a9 may be able to partly mediate the *181-Rik* function in regulating the inflammatory response.

### *181-Rik* knockdown impairs inflammatory response in primary microglia

**Fig. 8:**
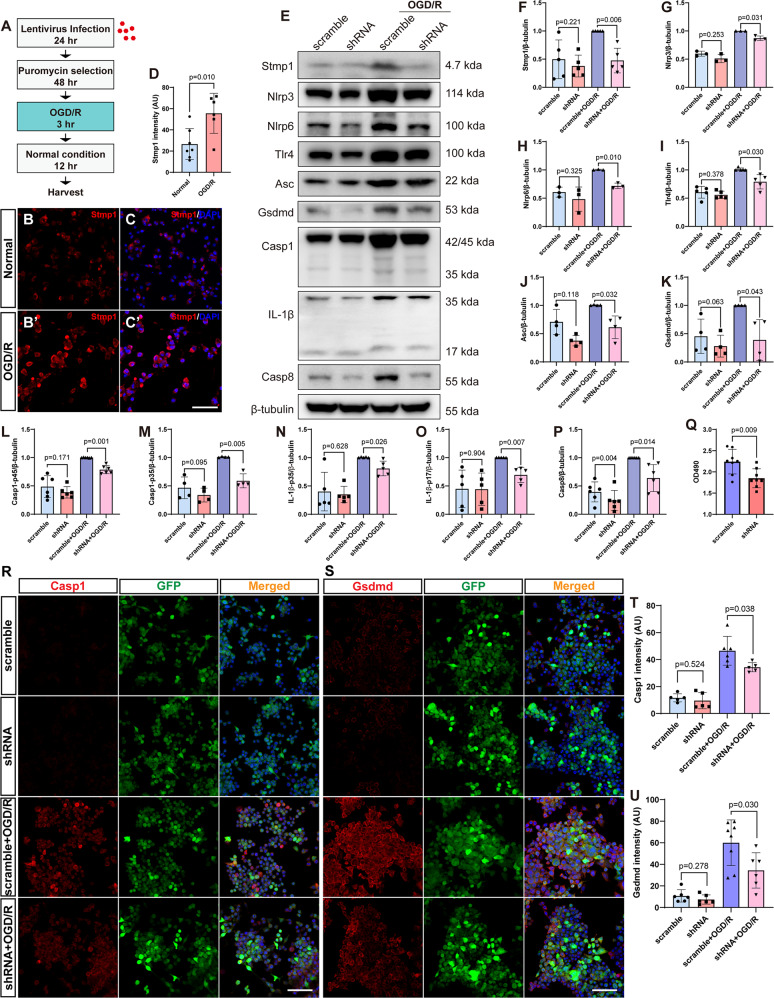
*181-Rik* knockdown inhibits the Nlrp3 inflammasome pathway in primary microglia. **A** Workflow of the establishment of the oxygen-glucose deprivation and reperfusion (OGD/R) cell model from mouse primary microglia. **B**, **C**, **B**′, **C**′ Immunostaining of normal and OGD/R-injured microglia with an anti-Stmp1 antibody revealed Stmp1 upregulation upon OGD/R treatment. Scale bar: 100 μm. **D** Quantification of Stmp1 immunofluorescence intensity in normal and OGD/R-treated microglia. Data were presented as mean ± SD (*n* = 6) in arbitrary unit (AU). **E** Western blotting analysis was carried out for Stmp1 and the indicated Nlrp3 inflammasome pathway-related proteins expressed in normal and OGD/R-injured microglia infected with lentiviruses expressing the scramble or *181-Rik* shRNA. β-tubulin served as the internal protein control. **F**–**P** Quantification of relative protein expression levels in the four groups of microglia (scramble, shRNA, scramble+OGD/R, and shRNA+OGD/R). Data were presented as mean ± SD (*n* = 3–6). **Q** LDH release (OD490) from microglia infected with lentiviruses expressing the scramble or *181-Rik* shRNA. Data were presented as mean ± SD (*n* = 8). **R**, **S** Double-immunostaining of normal and OGD/R-injured microglia infected with lentiviruses expressing the scramble or *181-Rik* shRNA with antibodies against GFP and Casp1 or Gsdmd. They were also counterstained with nuclear DAPI (blue). All viruses express the GFP reporter to indicate successful cell infection. Scale bar: 100 μm. **T**, **U** Quantification of immunofluorescence intensity of Casp1 and Gsdmd in the four groups of microglia (scramble, shRNA, scramble+OGD/R, and shRNA+OGD/R). Data were presented as mean ± SD (*n* = 5–8) in arbitrary unit (AU).

To further investigate the function of *181-Rik* in inflammation, we established an OGD/R cell model from murine primary microglia (Fig. 8A). Following the OGD/R challenge, Stmp1 micropeptide immunofluorescence was seen to be increased by more than twofold (Fig. 8B–D, B′, C′), implicating a potential role of Stmp1 in OGD/R injury. Indeed, consistent with in vivo results (Fig. 4), Western blotting showed that in OGD/R-treated microglia, knockdown of *181-Rik* expression by shRNA significantly reduced the expression of Nlrp3, Nlrp6, Tlr4, Asc, Gsdmd, Casp1, IL-1β, and Casp8 proteins, compared to that in the scramble shRNA controls (Fig. 8E–P and S9A). In addition, Stmp1 was also markedly upregulated in scramble shRNA control cells upon OGD/R treatment (Fig. 8E, F). Since lactate dehydrogenase (LDH) release is reported to correlate with Nlrp3 inflammasome activation [60], we also examined LDH release from microglia infected with lentiviruses expressing the scramble or *181-Rik* shRNA. The result showed that *181-Rik* knockdown decreased LDH release by 17.46% (Fig. 8Q). We further validated the change of Casp1 and Gsdmd expression by immunolabeling. As expected, at the normal condition, only low levels of Casp1 and Gsdmd immunoreactivity were detected in microglia infected with either the scramble shRNA or *181-Rik* shRNA lentiviruses (Fig. 8R–U). Casp1 and Gsdmd immunoreactivity was obviously increased in OGD/R-treated microglia, but *181-Rik* knockdown resulted in a significant reduction in immunofluorescence of both markers (Fig. 8R–U), in agreement with the finding that decreased *181-Rik* expression negatively affects inflammation and pyroptosis.

Since *181-Rik* is expected to exert its function through the encoded Stmp1 micropeptide, we attempted to rescue its knockdown phenotype by lentivirus-mediated Stmp1 expression in cultured microglial cells. By qRT-PCR assay, we found that Stmp1 overexpression restored the RNA expression levels of G*sdmd, Casp1, Casp8, IL-1β*, and *IL-18* to control or even higher levels in OGD/R-treated microglia with knocked-down *Rik-181* (Fig. S10A). Similarly, Western blotting showed that Stmp1 overexpression elevated protein expression levels of Nlrp3, Asc, Gsdmd, IL-1β, and Casp1 compared to *181-Rik* knockdown alone (Fig. S10B), suggesting that Stmp1 micropeptide is able to promote inflammation and pyroptosis via the Nlrp3 inflammasome pathway.

### Mitochondrial changes caused by *181-Rik* ablation or knockdown

**Fig. 9:**
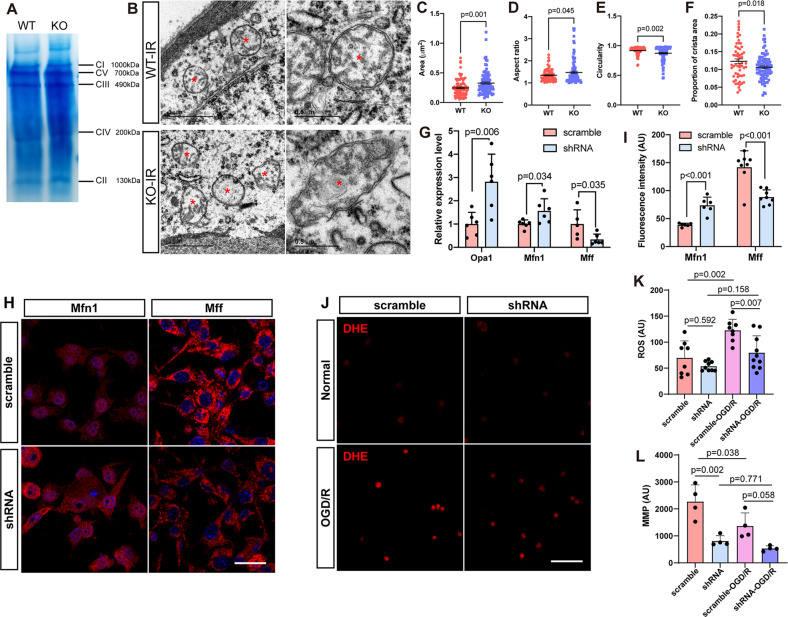
Mitochondrial alterations caused by *181-Rik* ablation or knockdown. **A** BN-PAGE (blue native polyacrylamide gel electrophoresis) visualizes the integrity of the indicated respiratory complexes (CI-V) in mitochondria of *181-Rik* WT and KO mouse telencephalons. **B** Transmission electron microscopy of microglial mitochondria from WT and KO retinas at 2 days post-IR injury. Red asterisks denote mitochondria. Scale bars: 1 μm (left panels) and 0.5 μm (right panels). **C**–**F** Quantification of the mitochondrial area (**C**), aspect ratio (**D**), circularity (**E**), and crista area proportion (**F**). Data were presented as mean ± SD [*n* = 91 and 97 (**C**), 89 and 97 (**D**), 90 and 97 (**E**), and 57 and 88 (**F**) for WT and KO retinas, respectively]. **G**–**I** qRT-PCR assay of mitochondrial fusion-related genes *Opa1* and *Mfn1* and fission-related gene *Mff* (**G**), immunostaining for Mfn1 and Mff (**H**), and quantification of the corresponding immunofluorescence intensity (**I**) in microglia infected with *181-Rik* shRNA or scramble lentiviruses. Data were presented as mean ± SD [*n* = 5 or 6 (**G**) and 6–8 (**I**)]. AU arbitrary unit. Scale bar: 25 μm. **J**–**L** DHE (dihydroethidium) labeling (**J**), quantification of the corresponding immunofluorescence (representing ROS) intensity (**K**), and MMP (mitochondrial membrane potential) measurements (**L**) in the four groups of microglia (scramble, shRNA, scramble+OGD/R, and shRNA+OGD/R). Data were presented as mean ± SD [*n* = 8–10 (**K**) and 4 (**L**)] in arbitrary unit (AU). Scale bar: 100 μm.

Given the location of *181-Rik*-encoded Stmp1 in mitochondria, there is a possibility that *181-Rik* may affect the inflammatory response of microglia by regulating mitochondrial functions. Stmp1 was previously speculated to be a subunit of the mitochondrial respiratory complex [61] and micropeptides such as Brawnin and Uqcc3 are involved in the assembly of the respiratory CIII and hence affect its stability [30, 37]. We, therefore, determined whether *181-Rik* ablation had any deleterious effect on respiratory complex stability. We isolated mitochondria from telencephalic tissues of adult WT and KO mice, extracted mitochondrial membrane proteins with Digitonin, and performed non-denaturing polyacrylamide gel electrophoresis. Clear bands of complexes CI-V were visualized and no obvious differences were observed between the WT and KO bands (Fig. 9A). Because unstable complexes appear as fuzzy or abnormally sized bands during non-denaturing electrophoresis [45], this result suggests that *181-Rik* inactivation may have no effect on the stability of respiratory complexes at a gross level.

Mitochondria undergo constant fission and fusion [23]. We, therefore, investigated whether *181-Rik* inactivation affected this dynamics. By transmission electron microscopy, we visualized and compared the mitochondria of microglia located around the GCL of IR-injured WT and KO retinas (Fig. 9B). By quantification of several morphological parameters, we found that the area of KO mitochondria was significantly larger than that of WT mitochondria and so was the aspect ratio (longer diameter/shorter diameter); while the circularity and proportion of crista area of KO mitochondria were significantly lower than those of the WT controls (Fig. 9B–F). These results indicate that the KO mitochondria are larger and closer to an ellipse in shape than the WT ones, with rougher surface and decreased crista surface ratio, suggesting that the absence of *181-Rik* may promote mitochondrial fusion. It has been shown that Opa1 participates in IMM fusion, Mfn1 in OMM fusion, and Mff in OMM fission [62, 63]. We thus examined whether 1*81-Rik* would affect their expression and found by qRT-PCR that the expression of both *Opa1* and *Mfn1* RNA was significantly upregulated by *181-Rik* knockdown in primary microglia whereas *Mff* was downregulated (Fig. 9G). In agreement, Mfn1 immunoreactivity was increased while Mff immunoreactivity was decreased by 1*81-Rik* knockdown (Fig. 9H, I), suggesting that *181-Rik* may influence mitochondrial dynamics by regulating the expression of Opa1, Mfn1, and Mff.

Since ROS produced by mitochondria is a major driver for activating immune cells and producing inflammatory factors [19, 20, 22], we used the DHE (dihydroethidium) probe to detect the ROS content in primary microglia with *181-Rik* knocked down. In NT cells, there were only weak DHE fluorescent signals whereas OGD/R treatment resulted in bright signals which were reduced by approximately 40% by *181-Rik* knockdown (Fig. 9J, K). The MMP is important for ROS production so we measured MMP in four groups of control and treated microglia (Fig. 9L). In both OGD/R-treated and untreated microglia, *181-Rik* knockdown caused a ~60% decrease in MMP compared to the scramble control (Fig. 9L). Thus, *181-Rik* appears to be required for ROS production and MMP maintenance, thereby affecting inflammatory response.

It has been shown that overexpression of the uncoupling protein Ucp2 can reduce MMP and thus decrease ROS production [24–27]. Additionally, Ucp2 has been demonstrated to inhibit mitochondrial fission [27–29] and regulate cell metabolism [26, 64, 65]. We, therefore, analyzed its expression by qRT-PCR and found that *181-Rik* knockdown increased *Ucp2* RNA expression level by more than 2.5-fold in microglia (Fig. S11A). Immunofluorescence labeling confirmed this result at the protein expression level in microglial cells (Fig. S11D, E), suggesting that Ucp2 may be able to partly mediate the mitochondrial function of *181-Rik*.

In general, activated immune cells tend to undergo aerobic glycolysis, which is accompanied by an increase in glucose metabolism, meanwhile, reduce the metabolism of fatty acids and amino acids to provide more raw materials for the synthesis of inflammatory factors [66–69]. Since Ucp2 was reported to regulate metabolism [70], we examined by qRT-PCR assay expression changes of genes involved in glucose, fatty acid, and glutamine metabolism in primary microglia resulting from the *181-Rik* knockdown. *181-Rik* knockdown downregulated the expression of glucose metabolism-related genes *Hk1, Hk2, Pkm, Ldha, Glut1*, and *Vdac1* by 41–83% (Fig. S11B). However, it upregulated the expression of fatty acid metabolism-related genes *Cpt1a, Vlcad, Hadha*, and *Hadhb* by 29–181% (Fig. S11C). Consistent with their RNA expression changes, subsequent immunofluorescence staining showed that Glut1, Hk2, and Pkm immunoreactivity was significantly decreased whereas Hadha and Cpt1a immunoreactivity was significantly increased by *181-Rik* knockdown (Fig. S11D, E). In addition, *181-Rik* knockdown upregulated glutamine metabolism-related genes *Slcla5* and *Gls* by ~130% (Fig. S11F), suggesting that *181-Rik* may normally promote glucose metabolism but inhibit fatty acid and glutamine metabolism.

Stmp1 is recently reported to interact with mitochondrial respiratory complex IV (CIV) and enhance its activity in tumor cells [57]. We, therefore, measured the CIV activity of mitochondria isolated from primary microglia infected with lentiviruses expressing the scramble or *181-Rik* shRNA. By calculating the slopes of the absorbance curves, we found that the reaction rate of CIV in microglia with *181-Rik* knockdown was 10.94% lower than that with the scramble (Fig. S12), consistent with the previous finding that Stmp1 is involved in CIV activity [57].

## Discussion

**Fig. 10:**
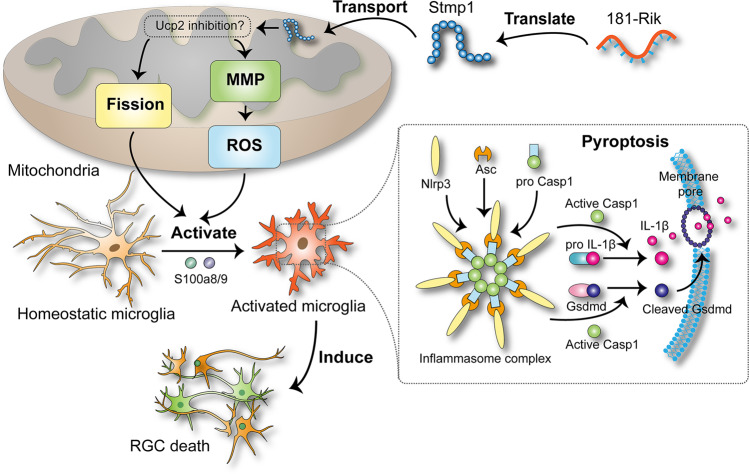
Work model of *181-Rik* function in the mitochondria-mediated inflammatory response of microglia. LncRNA *181-Rik* is translated into a 47-AA mitochondrial micropeptide Stmp1, which triggers mitochondrial fission and increases MMP and ROS production. As one possible mechanism, these effects may be exerted through the inhibition of Ucp2 expression by Stmp1. These mitochondrial changes contribute to activating microglia as well as the Nlrp3 inflammasome pathway, which results in microglial pyroptosis and the release of cytokines, eventually leading to RGC death during retinal IR injury. S100a8 and S100a9 appear to function downstream of *181-Rik* in the activation of inflammasomes.

In this study, we have found that a widely distributed lncRNA *181-Rik* is translated into a 47-AA conserved micropeptide Stmp1 located in mitochondria. Its inactivation leads to the suppression of retinal inflammation and microglial activation, thereby protecting RGCs from apoptosis after retinal IR injury. Stmp1 appears to exert its function by regulating mitochondrial functions (Fig. 10).

### *181-Rik*-encoded Stmp1 stimulates neuroinflammatory response by regulating mitochondrial and metabolic functions

In this work, we set out to identify lncRNAs, in particular micropeptide-encoding lncRNAs, that would regulate RGC survival during retinal IR injury and have indeed identified one, *181-Rik*. It is predicted to encode a conserved 47-AA micropeptide homologous to the zebrafish Stmp1, which was presumed to be a putative subunit of the mitochondrial respiratory complex [61]. Indeed, by Western blotting, immunostaining, protein extraction and separation, and analysis of existing RNA-seq and ribosomal profiling datasets, we are able to confirm that Stmp1 is translated and located specifically to both the IMM and OMM of mitochondria. In agreement, the mitochondrial location has recently also been demonstrated for Mm47/Stmp1 in mouse embryonic fibroblasts [53]. Interestingly, an unknown bovine protein PL-5283 (now clearly the Stmp1 paralog) was previously identified by tandem mass spectrometry of protein ions, which was co-purified with the respiratory complex CIII [52].

Given the localization of Stmp1 in mitochondrial membranes, we have speculated that it may participate in the regulation of mitochondrial functions (Fig. 10), and indeed this has turned out to be the case. Previously, it has been shown that mitochondria are enriched for non-coding RNA-encoded micropeptides, many of which are involved in mitochondrial metabolism, OXPHOS and respiratory complex assembly [30]. Because of the apparent binding of Stmp1 to CIII [52], we conducted non-denaturing gel electrophoresis to monitor the intactness of CIII in mitochondria of *181-Rik* deficient microglia but observed no obvious changes, suggesting that Stmp1 may not be involved in the assembly of mitochondrial respiratory complexes. On the other hand, *181-Rik* deficiency caused a significant increase in mitochondrial area and aspect ratio but a decrease in the circularity and proportion of crista area, indicating a clear mitochondria dynamic change. Therefore, the mutant mitochondria are larger with a rougher surface and reduced crista surface ratio, suggesting that *181-Rik* inactivation may facilitate mitochondrial fusion. In agreement, a recent study reported that Stmp1 could enhance mitochondrial fission to promote tumor metastasis [71]. This effect may be caused by the altered expression of Opa1, Mfn1, and Mff given the observed upregulation of Opa1 and Mfn1 and downregulation of Mff in *181-Rik* knocked-down microglia, and is consistent with the finding that Opa1 and Mfn1 participate in IMM/OMM fusion whereas Mff in OMM fission [63].

Aside from mitochondrial dynamics, we have provided evidence to show that *181-Rik* has also an important regulatory role in ROS production. We found that the ROS level of microglia increased after OGD/R treatment, consistent with previous findings [17], but was significantly decreased by knocking down *181-Rik* expression; and so was MMP. The MMP change is expected since ROS is primarily generated during OXPHOS by the electron transport chain consisting of respiratory complexes whose activities are supported by MMP [72–75]. Therefore *181-Rik* may control ROS production by affecting MMP maintenance. ROS is known to widely regulate immune response-it not only plays an important role in the activation, polarization, and antibacterial defense of macrophages, but also promotes the maturation of B and T cells [19, 20, 22]. More importantly, it directly promotes the activation of Nlrp3 inflammasome and the subsequent maturation of cytokines such as IL-1β [76–78]. Thus, *181-Rik*-encoded Stmp1 may stimulate a neuroinflammatory response in part by driving ROS production (Fig. 10).

*181-Rik* appears to also play a crucial role in controlling oxidative metabolism. First, its knockdown in microglia resulted in the downregulation of glucose oxidation-related genes but upregulation of fatty acid oxidation-related and glutamine oxidation-related genes, suggesting that *181-Rik* may contribute to maintaining balanced metabolism by promoting glucose oxidation but inhibiting the oxidation of both fatty acids and glutamine. This is interesting because activated immune cells usually prefer the oxidation of glucose rather than fatty acids and amino acids [66]. For instance, to support the functions of rapid mobility, ROS production, cytokine release, and phagocytosis in pro-inflammatory response, activated macrophages prefer aerobic glycolysis rather than OXPHOS for a rapid energy supply, which is accompanied by increased glucose uptake; on the other hand, they need abundant materials such as fatty acids and amino acids to synthesize inflammatory factors or signal molecules, so they prevent these molecules from oxidation [67–69]. Therefore, *181-Rik* may facilitate microglial inflammation in part by regulating metabolism.

Our experimental data indicate that *181-Rik* may control neuroinflammatory response through multiple pathways. The detailed mechanism of how *181-Rik* acts is not quite clear at present but one possibility may be by suppressing the expression of the metabolic sensor Ucp2 (Fig. 10). In microglia, *181-Rik* knockdown resulted in upregulation of Ucp2 expression at both RNA and protein levels. Previous studies have shown that Ucp2 inhibits mitochondrial ROS production by reducing MMP [26, 72–75]. It is also involved in the selection of substrates for mitochondrial oxidation. It reduces the oxidation of glucose-derived pyruvate but is beneficial to the oxidation of fatty acids and glutamine in macrophages and other cells [26, 64, 65, 79–81]. In addition, Ucp2 has been demonstrated to inhibit mitochondrial fission. Mitochondria in *Ucp2*-deficient mice/cells tended to be fragmented whereas Ucp2-overexpressing mice/cells showed the opposite effect [27–29]. As a result, macrophages lacking *Ucp2* displayed increased activation and *Ucp2* knockout mice suffered from exacerbated inflammation [24–26]. Because of the strong correlation between *181-Rik*-deficiency phenotypes and the Ucp2 properties, we tentatively propose that *181-Rik* may perform multiple regulatory functions in mitochondrial dynamics, ROS generation, and oxidation metabolism to exacerbate inflammatory response by inhibiting the expression of a key downstream mediator Ucp2 (Fig. 10).

At present, it remains to be determined how *181-Rik* may indirectly or directly control Ucp2 expression. Both *181-Rik* encoded Stmp1 and Ucp2 are located in the IMM, and Stmp1 is supposed to interact with CIII [52, 82] and CIV [57], the sites where protons are pumped from the matrix into the intermembrane space. Stmp1 is reported to play a role in facilitating the activities of CIII and CIV [52, 82] whereas Ucp2 appears to play the opposite role [26]. Thus, we speculate that by inhibiting the Ucp2 activity, Stmp1 may function as an accessory protein of CIII and CIV to assist in the maintenance of proton pumping. The expression of *181-Rik*/Stmp1 may prevent Ucp2 from being transcribed or translated by some currently unknown mechanisms. Once *181-Rik*/Stmp1 is eliminated, the expression level of Ucp2 would be upregulated. Further studies are needed to understand the relationship between Stmp1 and Ucp2 and their functional regulation.

### *181-Rik*-encoded Stmp1 regulates IR-induced RGC death by facilitating microglia-mediated neuroinflammation

Retinal IR injury is known to cause RGC death [3] but we have demonstrated that *181-Rik* inactivation ameliorates this effect. We found that in IR-treated *181-Rik* KO retinas, apoptosis decreased while survived RGCs and RGC marker gene expression both increased, suggesting that the absence of *181-Rik* provides a protective effect on RGCs. Since *181-Rik* deletion does not appear to cause obvious deleterious phenotypes, targeting *181-Rik* by gene editing, RNA interference or peptide depletion may offer a promising avenue to develop therapeutics for the prevention and treatment of retinal IR-mediated RGC injury in ocular diseases such as glaucoma and diabetic retinopathy.

Retinal IR can induce a strong inflammatory response leading to RGC death [2, 3] and we have shown that *181-Rik* deficiency ameliorates this detrimental response. In IR-treated retinas, *181-Rik* inactivation led to the downregulation of a series of Nlrp3 inflammasome-related genes including *Tlr4, Nlrp3, Asc, Gsdmd, Casp1*, and *IL-1β*, at RNA and/or protein levels. Similarly, microglia with *181-Rik* knocked down were compromised for Nlrp3 activation following OGD/R injury. Because the Stmp1 micropeptide was upregulated upon IR- or OGD/R treatment of retinas or microglia and Stmp1 overexpression in *181-Rik*-deficient microglia was able to rescue impaired Nlrp3 inflammasome activation, *181-Rik* is likely to trigger Nlrp3 inflammasome activation via Stmp1 both in vivo and vitro (Fig. 10). Previously, *181-Rik* deletion in macrophages was shown to exert no effect on the expression levels of pro-IL-1β, pro-Casp1 and Nlrp3 proteins, so *181-Rik* was thought to be involved only in Nlrp3 inflammasome licensing/activation but not in the priming process [53]. By contrast, we found that *181-Rik* ablation resulted in downregulated RNA levels of Nlrp3 inflammasome-related genes such as *Asc, Gsdmd*, and *Casp1* meanwhile *181-Rik* deletion/knockdown in mice or microglia decreased protein levels of pro-Casp1 and pro-IL-1β, suggesting that *181-Rik* may be involved in Nlrp3 inflammasome priming as well.

Retinal IR causes inflammation by activating resident microglia [83, 84] and *181-Rik* clearly regulates this activation process because: (1) in IR-treated *181-Rik* KO mice, we observed obviously fewer activated microglia in both the retina and optic nerve. This phenomenon may be partly explained by the decreased proliferation of microglia in KO retinas; (2) the morphology and marker expression of KO microglia resembled more to the homeostatic microglia than the activated ones. In addition, phagocytosis of RGC by activated microglia was also inhibited upon *181-Rik* inactivation; and (3) scRNA-seq and immunolabeling analyses revealed an alteration in the composition of microglial subpopulations in IR-treated KO retinas. For instance, the proportion of MG0 homeostatic microglia increased in the KO retina. All these evidence indicates that *181-Rik* would normally promote microglial activation. Therefore, despite other possible pathways, one principal mechanism by which *181-Rik* regulates IR-induced RGC death may be by activating microglia and inflammasomes such that the activated microglia eventually undergo pyroptosis and release inflammatory factors to induce RGC death (Fig. 10). Nevertheless, it is not possible to gauge the extent of RGC protection mediated specifically by microglia using our current conventional *181-Rik* knockout mice. A microglia-specific *181-Rik* knockout is needed to address this issue in the future.

### S100a8/a9 mediates *181-Rik* function in Nlrp3 inflammasome activation

In this work, we have identified by scRNA-seq analysis a subpopulation (MG8) of microglial cells that appear to be pro-inflammatory and regulated by *181-Rik*. The MG8 microglia are characterized by the expression of both S100a8 and S100a9, which are cellular Ca^2+^ binding proteins and Ca^2+^ sensors and have been shown to promote inflammation via the Nlrp3 inflammasome pathway [59, 85]. We thus consider MG8 as a pro-inflammatory microglia cluster. Consistent with this idea, in IR-treated *181-Rik* KO retinas, the number of MG8 microglia was greatly reduced and so were the RNA expression levels of *S100a8* and *S100a9*. Correspondingly, we observed downregulated expression of S100a8/a9 proteins as well as a decreased number of S100a8- and S100a9-immunoreactive microglia in the KO retina. S100a8 knockdown in microglial cells led to the expected downregulation of Nlrp3 inflammasome pathway-related genes *Casp1, IL1β*, and *IL-18* following OGD/R treatment. Therefore, *181-Rik* may trigger Nlrp3 inflammasome activation by activating S100a8/a9 expression.

In summary, we have identified a lncRNA *181-Rik* that encodes a mitochondrion-located micropeptide Stmp1. Its inactivation in mice protects RGCs from retinal IR injury by inhibiting the activation of microglia and inflammasomes. Its deficiency in mice or primary microglia leads to altered mitochondrial dynamics, reduced MMP and ROS, shifted balance in glucose, fatty acid, and amino acid metabolism, and diminished inflammation. By inhibiting Ucp2 expression and activating the expression of Ca^2+^ sensors S100a8/a9, *181-Rik* appears to regulate mitochondrial and metabolic functions to activate the Nlrp3 inflammasome pathway. Our findings thus provide new insights into the pathogenesis caused by retinal IR injury and may provide a fresh target to develop therapeutics for the prevention and treatment of IR-associated neurodegenerative diseases such as glaucoma and diabetic retinopathy.

## Supplementary information

Supplementary Materials

Table S1

Full and Uncropped Western Blots

Reproducibility_checklist

## Data Availability

The scRNA-seq data have been deposited in the NCBI Sequence Read Archive (SRA) database under accession code PRJNA907755. The data supporting the findings of this study are available within the article and its supplementary information files. Additional data are available from the corresponding author upon reasonable request.
