# Supplementary material for: A lncRNA-encoded mitochondrial micropeptide exacerbates microglia-mediated neuroinflammation in retinal ischemia/reperfusion injury
Source: Cell Death Dis. 2023 Feb 15;14(2):126. doi: 10.1038/s41419-023-05617-2 (PMC9932084; doi:10.1038/s41419-023-05617-2)
Supplement: Supplementary file 1 — Supplementary Materials [file 41419_2023_5617_MOESM1_ESM.pdf]

A lncRNA-encoded mitochondrial micropeptide exacerbates microglia-mediated neuroinflammation in retinal ischemia/reperfusion injury

Xintong Zheng[1], Mingwei Wang[1], Shuting Liu[1], Haiqiao Chen[1], Yifei Li[1], Fa Yuan[1], Ludong Yang[1], Suo Qiu[1], Hongwei Wang[1], Zhi Xie[1], and Mengqing Xiang[1,2,*]

[1]State Key Laboratory of Ophthalmology, Zhongshan Ophthalmic Center, Sun Yat-sen University, Guangdong Provincial Key Laboratory of Ophthalmology and Visual Science, Guangzhou 510060, China

[2]Guangdong Provincial Key Laboratory of Brain Function and Disease, Zhongshan School of Medicine, Sun Yat-sen University, Guangzhou, 510080, China

Supplementary Figures S1-12 and Tables S2-6

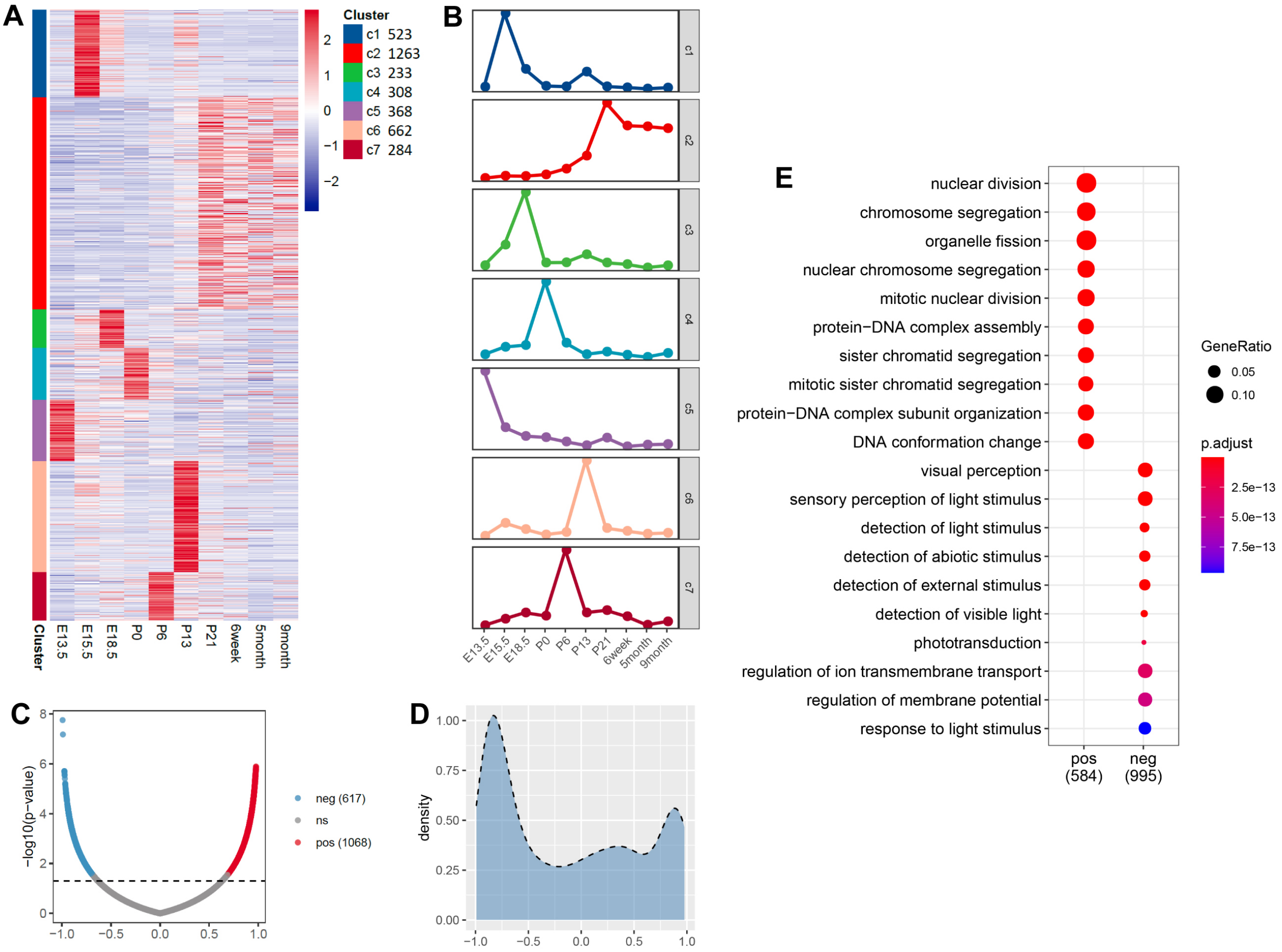

Figure S1. LncRNA expression patterns and correlation between *181-Rik* and differentially expressed genes during murine retinal development. (**A**) Heatmap of 3641 lncRNAs based on their Z-scored expression levels in the retina at the indicated stages (from E13.5 to 9 months) during mouse development. This analysis yielded 7 clusters c1-c7 which are color-labeled to the left and to the upper right along with the number of lncRNAs distributed in each cluster. (**B**) Plots of the mean expression of all lncRNAs in clusters c1-c7 during mouse retinal development. (**C**) Correlation between *181-Rik* expression and protein-coding genes differentially expressed during retinal development. Dotted line: -log10(p-value) = 1.30. neg: negatively correlated; ns: no significance; pos: positively correlated. There are 1068 positively correlated protein-coding genes and 617 negatively correlated ones. (**D**) Distribution of correlation coefficients of *181-Rik* relative to differentially expressed protein-coding genes. (**E**) Enrichment analyses of GO-biological process terms associated with protein-coding genes positively (pos) or negatively (neg) correlated with *181-Rik* expression.

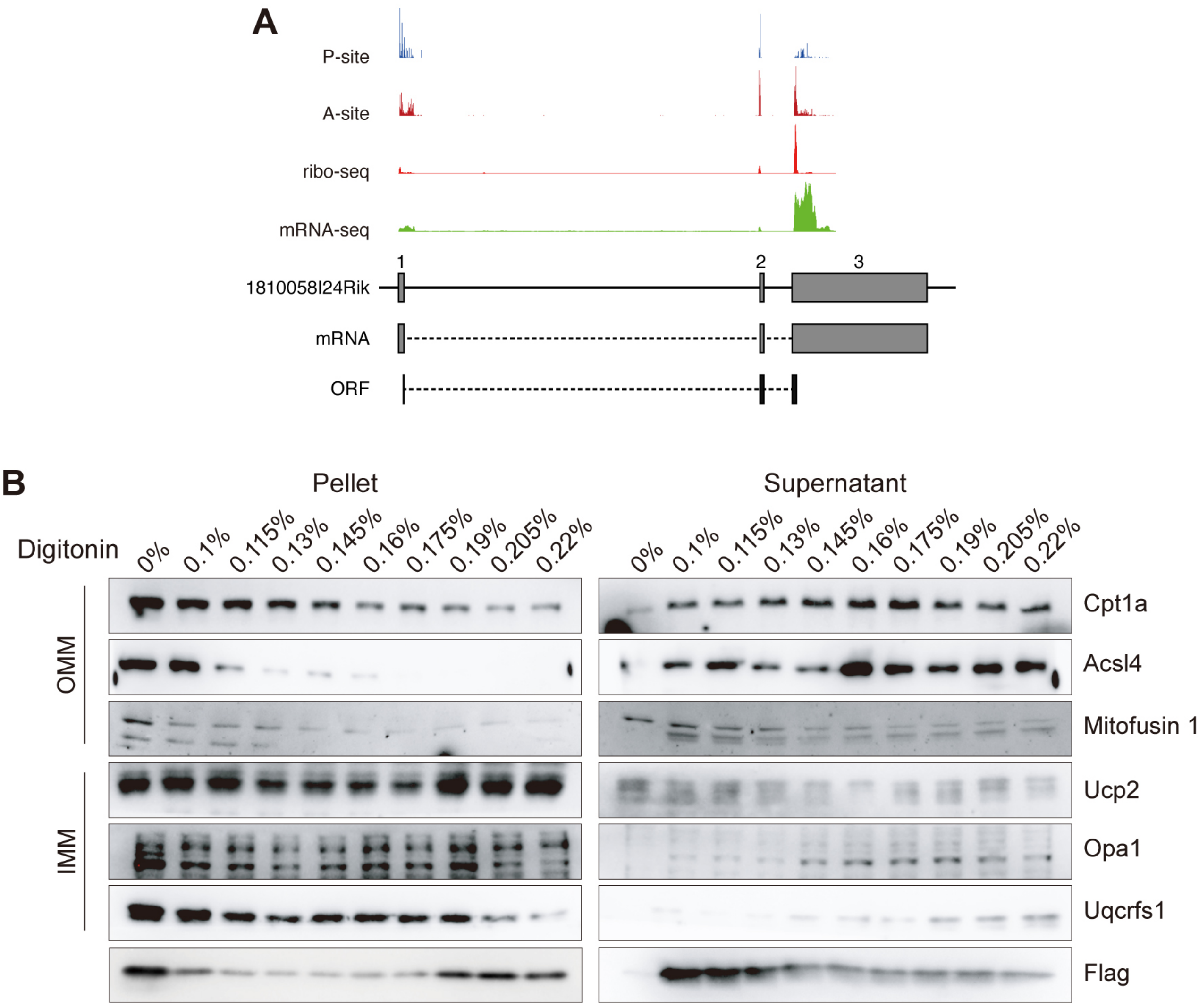

Figure S2. Analysis of *181-Rik* expression and Stmp1 suborganelle location. (**A**) Schematic diagram of the *181-Rik* gene, mRNA and open reading frame (ORF) as well as the corresponding mRNA-seq and ribo-seq tracks. The mRNA-seq, ribo-seq and A/P-site coverage plots are all from the GWIPS-viz browser. The ribo-seq reads peak at exons 1, 2 and the beginning part of exon 3, corresponding to the putative ORF region. A-site (for elongating ribosomes): aminoacyl site of ribosome that accepts the t-RNA; P-site (for initiating ribosomes): peptidyl site of ribosome that releases the t-RNA. (**B**) Western blotting analysis of mitochondrial proteins from NIH3T3 cells transfected with the Stmp1-Flag expression construct with the indicated antibodies. Mitochondria were isolated from cells and then extracted by serial concentrations of Digitonin. Proteins localized within the inner or outer mitochondrial membranes (IMM or OMM) had different distribution patterns in the extraction pellet and supernatant. Cpt1a, Acsl4 and Mitofusin1 are OMM proteins while Ucp2, Uqcrfs1 and Opa1 are IMM proteins. Stmp1-Flag had a distribution pattern characteristic of both IMM and OMM proteins.

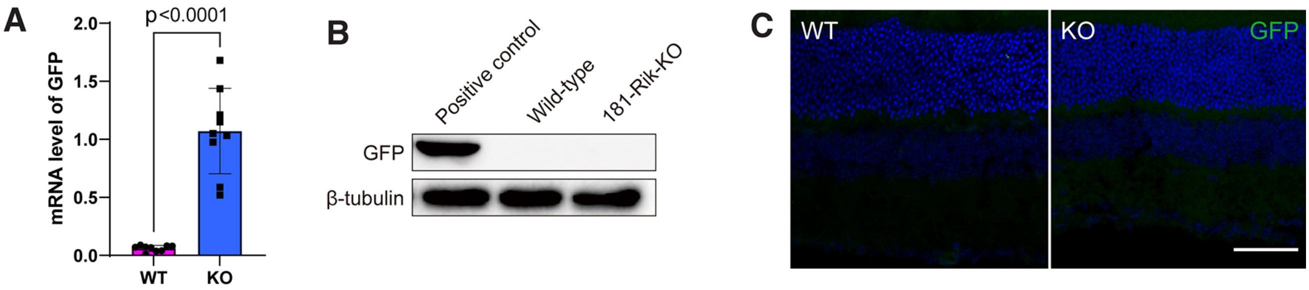

Figure S3. Absence of GFP protein expression in *181-Rik* mutant retinas. (**A**) qRT-PCR assay revealed a high GFP mRNA level in KO retinas. Data are presented as mean ± SD (n = 8 or 9). (**B**) Western blotting analysis showed the absence of GFP protein expression in both WT and KO retinas. GFP-expressing HEK293T cells served as the positive control and β-tubulin served as the internal protein control. (**C**) Immunostaining of WT and KO retinal sections did not detect GFP immunoreactivity in either WT or KO retinas. Scale bar: 50 μm.

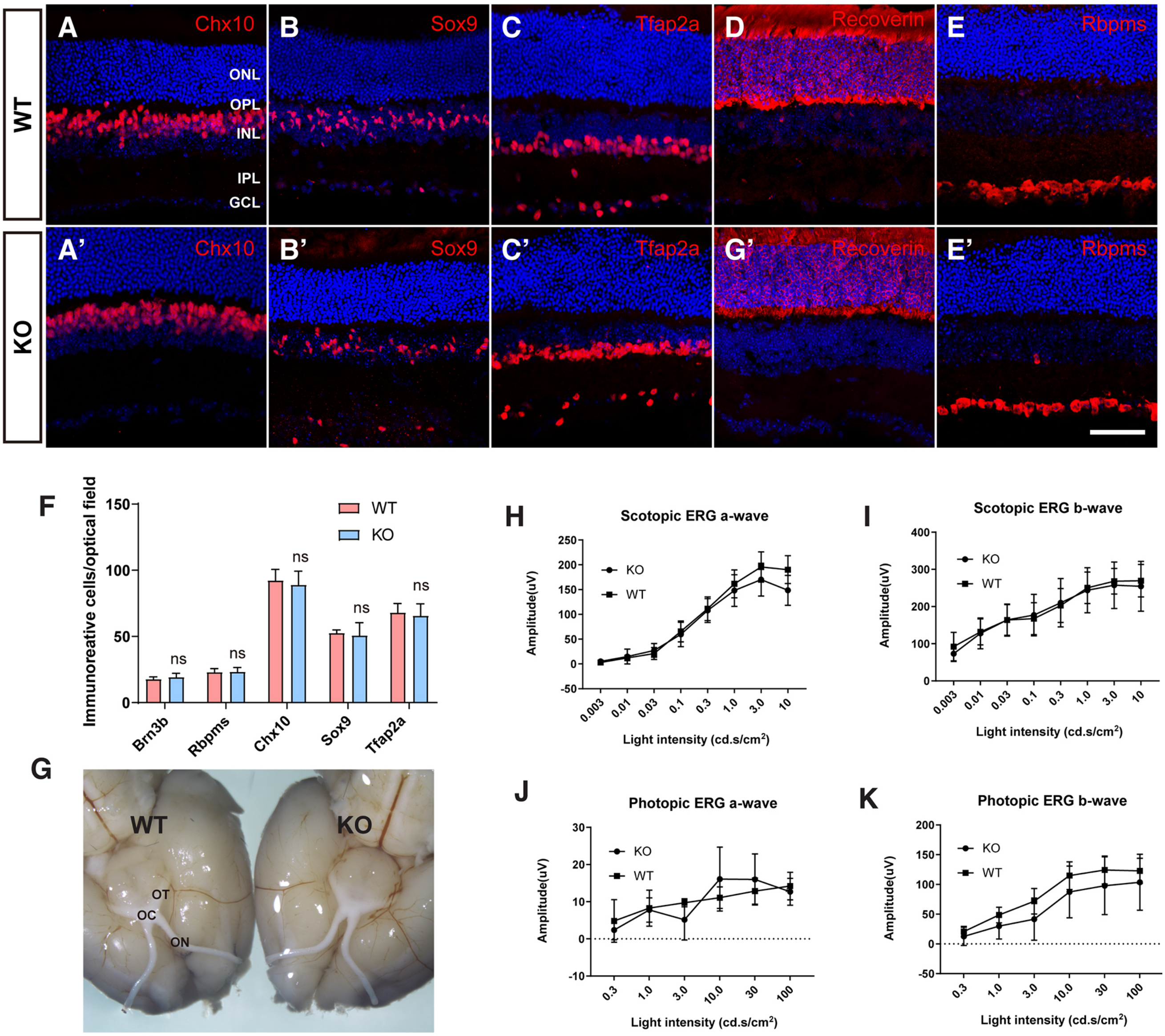

Figure S4. ***181-Rik*** **inactivation causes no obvious retinal phenotype.** (**A-E,A'-E'**) Immunostaining of normal WT and KO retinas with the indicated antibodies revealed no obvious difference in the number of major cell types in KO retinas. Chx10, Sox9, Tfap2a, Recoverin, and Rbpms are markers for bipolar, Müller, amacrine, photoreceptor, and ganglion cells, respectively. Scale bar: 50 µm. (**F**) Quantification of cells immunoreactive for the indicated protein markers in WT and KO retinas. Data are presented as mean ± SD (n = 3 or 4). (**G**) The size of the optic nerve (ON), optic tract (OT) and optic chiasm (OC) in adult WT and KO mice showed no obvious difference. (**H-K**) Scotopic and photopic electroretinogram (ERG) responses of adult WT and KO mice showed no significant differences in amplitudes of a and b waves. Data are presented as mean ± SD (n = 3 or 4). Abbreviations: GCL, ganglion cell layer; INL, inner nuclear layer; IPL, inner plexiform layer; ONL, outer nuclear layer; OPL: outer plexiform layer.

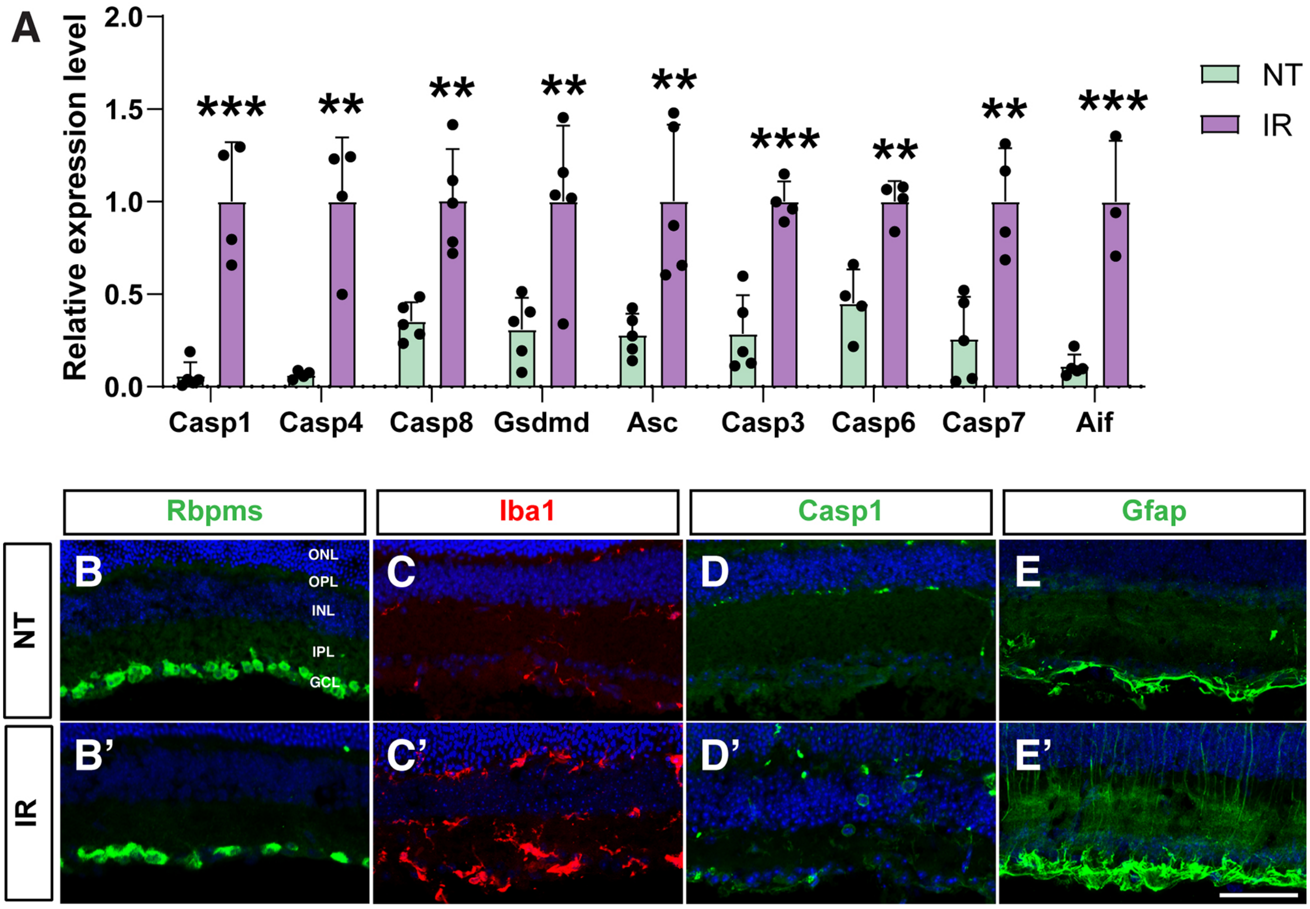

Figure S5. Validation of ischemia and reperfusion (IR) mouse models. (**A**) On day 2 post IR injury, qRT-PCR assay revealed significant upregulation of apoptotic and inflammatory genes in IR-injured wild-type retinas compared to untreated (NT) controls. Data are presented as mean$\pm$SD (n = 3 or 4), **p < 0.01, ***p < 0.001. (**B-E,B'-E'**) On day 2 post IR injury, retinal sections from untreated and IR-treated mice were immunostained with antibodies against Rbpms, Iba1, Casp1 or Gfap. All sections were also counterstained with nuclear DAPI (blue). Except for Rbpms, Iba1, Casp1 and Gfap all showed elevated immunoreactivity in IR-injured retinas. Scale bar: 50 μm. Abbreviations: GCL, ganglion cell layer; INL, inner nuclear layer; IPL, inner plexiform layer; ONL, outer nuclear layer; OPL: outer plexiform layer.

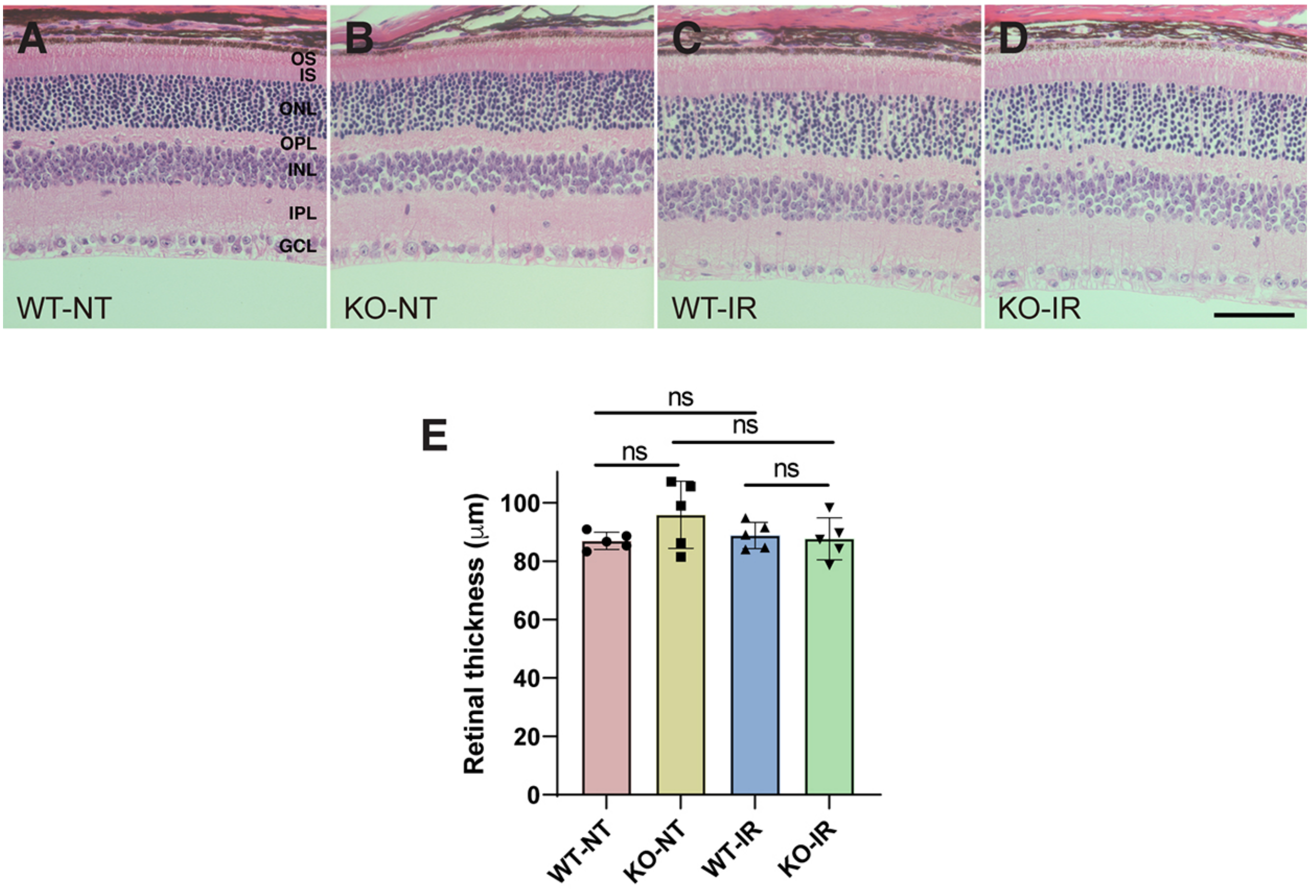

Figure S6. Central retinal thickness of IR-injured *181-Rik* mutant mice. (**A-D**) At 7 days post IR injury, untreated (NT) and treated (IR) WT and KO retinal sections were stained by hematoxylin-eosin (HE). Shown are images from the central region. Scale bar: 50 μm. (**E**) Measurements of central retinal thickness. Data are presented as mean ± SD (n = 5). Abbreviations: GCL, ganglion cell layer; INL, inner nuclear layer; IPL, inner plexiform layer; IS, inner segment; ns, no significance; ONL, outer nuclear layer; OPL: outer plexiform layer; OS, outer segment.

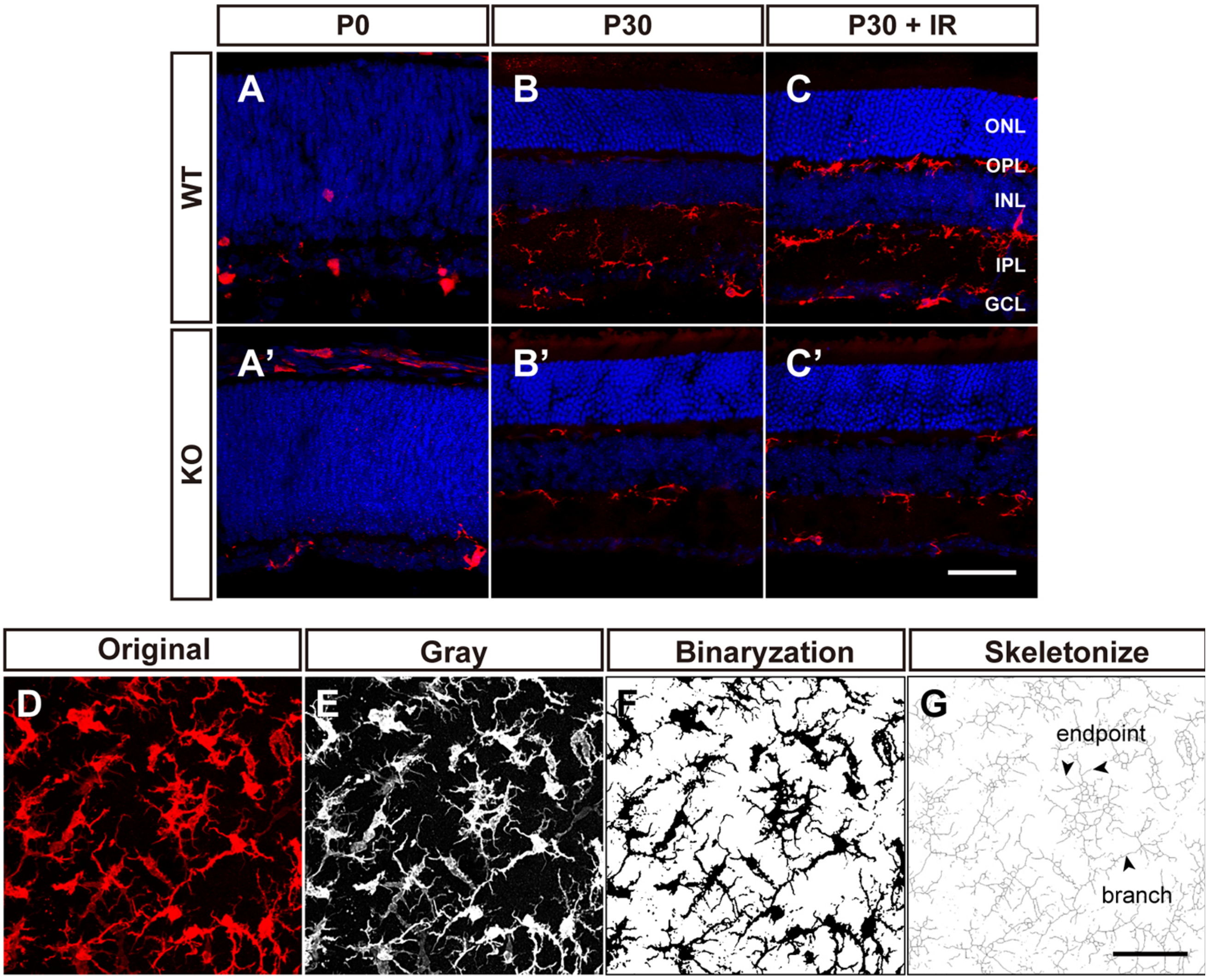

Figure S7. Analysis of microglial morphology in IR-injured *181-Rik* mutant retinas. (**A-C,A'-C'**) Iba1 immunoreactivity in P0 and P30 WT and KO retinal sections as well as in P30 retinal sections from WT and KO mice at 7 days post IR injury. Scale bar: 50 μm. (**D-G**) The process of microglial morphology analysis. The original image of Iba1 immunostaining in a retinal wholemount is converted into binary and skeletonized images using ImageJ. Representative endpoints and branch are indicated by arrowheads. Scale bar: 50 μm. Abbreviations: GCL, ganglion cell layer; INL, inner nuclear layer; IPL, inner plexiform layer; ONL, outer nuclear layer; OPL: outer plexiform layer.

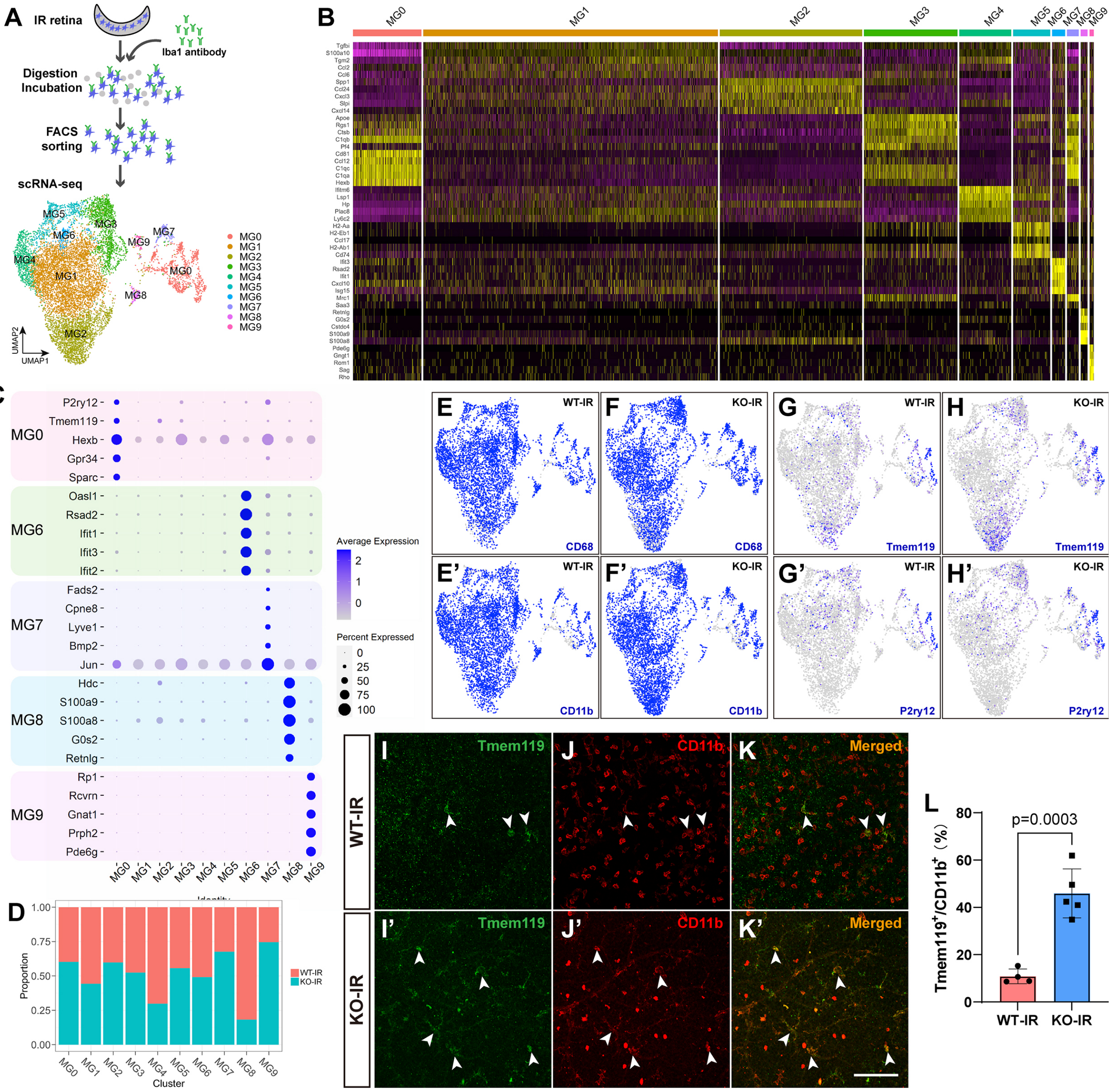

Figure S8. scRNA-seq analysis of microglia and their changes in IR-injured *181-Rik* mutant retinas. (**A**) Workflow of scRNA-seq analysis. Iba1-immunolabeled microglia were sorted by FACS (fluorescence-activated cell sorting) from pooled WT and KO retinas at 2 days post IR injury and subjected to scRNA-seq analysis. At the bottom is a UMAP plot of all single microglial cells from WT and KO retinas with the 10 clusters MG0-MG9 color-labeled. (**B**) Heatmap of expression of the top 5 enriched genes from each microglial cluster, grouped by the cluster name**. (C**) Five characteristic genes expressed in each of the indicated microglial clusters: MG0 and MG6-9. (**D**) Proportion of WT-IR and KO-IR microglia in each cluster. (**E-H,E'-H'**) UMAP plots colored by the expression of *CD68*, *CD11b, Tmem119*, or *P2ry12* in WT-IR and KO-IR microglia. (**I-K,I'-K'**) Double-immunostaining of WT and KO retinal wholemounts from mice at 2 days post IR-injury with antibodies against Tmem119 and CD11b. Scale bar: 100 μm. (**L**) Percentage of Tmem119^{+} microglia in WT-IR and KO-IR retinas. Data are presented as mean±SD (n = 4 or 5).

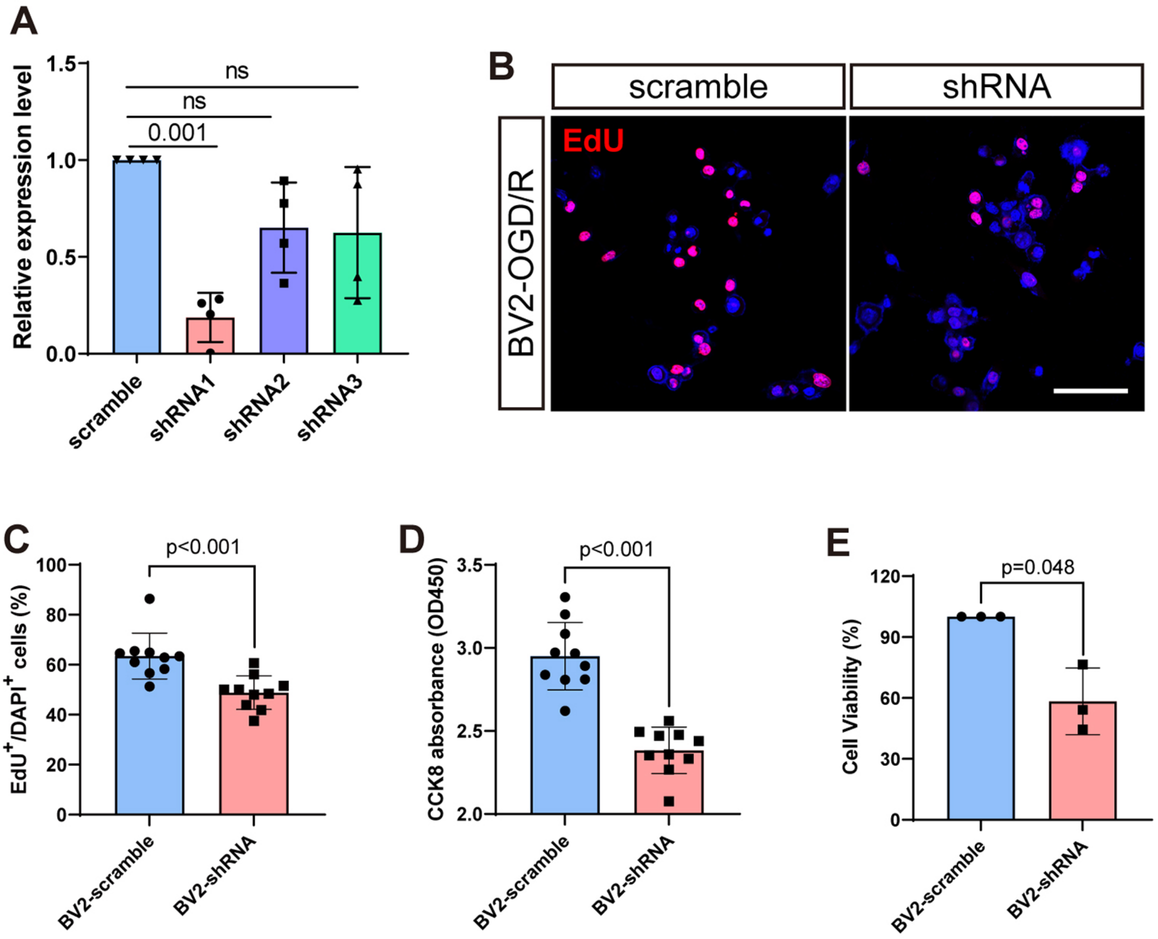

Figure S9. Inhibition of cell proliferation by *181-Rik* knockdown. (**A**) qRT-PCR assay of *181-Rik* RNA expression in primary microglia infected with scramble or *181-Rik* shRNA1-3 lentiviruses, comparing the knockdown effect of different *181-Rik* shRNAs. Data are presented as mean±SD (n= 4). shRNA1 had the best knockdown effect and was used in all following experiments. (**B**) EdU labeling (red) of OGD/R-treated BV2 cells infected with scramble or *181-Rik* shRNA lentiviruses. Cells were counterstained with nuclear DAPI (blue). Scale bar: 100 μm. (**C**) Quantification of EdU$^+$ cells in OGD/R-treated BV2 cells infected with scramble or *181-Rik* shRNA lentiviruses. Data are presented as mean±SD (n= 10). (**D**) CCK8 absorbance (OD450) of OGD/R-treated BV2 cells infected with scramble or *181-Rik* shRNA lentiviruses. Data are presented as mean±SD (n= 10). (**E**) Cell viability determined by the MTT (methyl thiazolyl tetrazolium) assay of OGD/R-treated BV2 cells infected with scramble or *181-Rik* shRNA lentiviruses. Data are presented as mean±SD (n= 3).

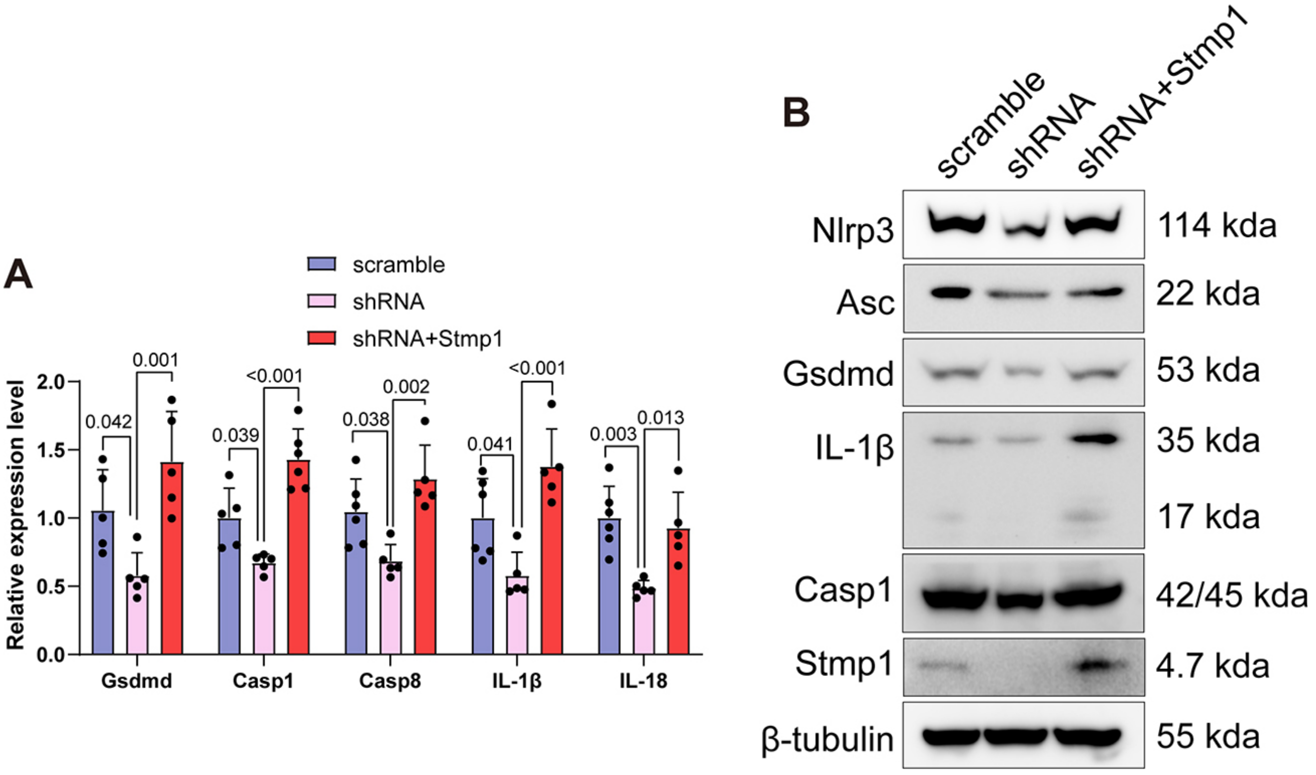

Figure S10. Rescue of the inhibitory effect of *181-Rik* knockdown on Nlrp3 inflammasome pathway by Stmp1 overexpression. (**A**) qRT-PCR assay of the indicated Nlrp3 inflammasome pathway-related genes in 3 groups of OGD/R-treated microglia (scramble, shRNA and shRNA+Stmp1). Data are presented as mean±SD (n= 5, 6). The p-values are indicated above the histograms. (**B**) Western blotting analysis was carried out for the indicated Nlrp3 inflammasome pathway-related proteins in 3 groups of OGD/R-treated microglia (scramble, shRNA and shRNA+Stmp1).

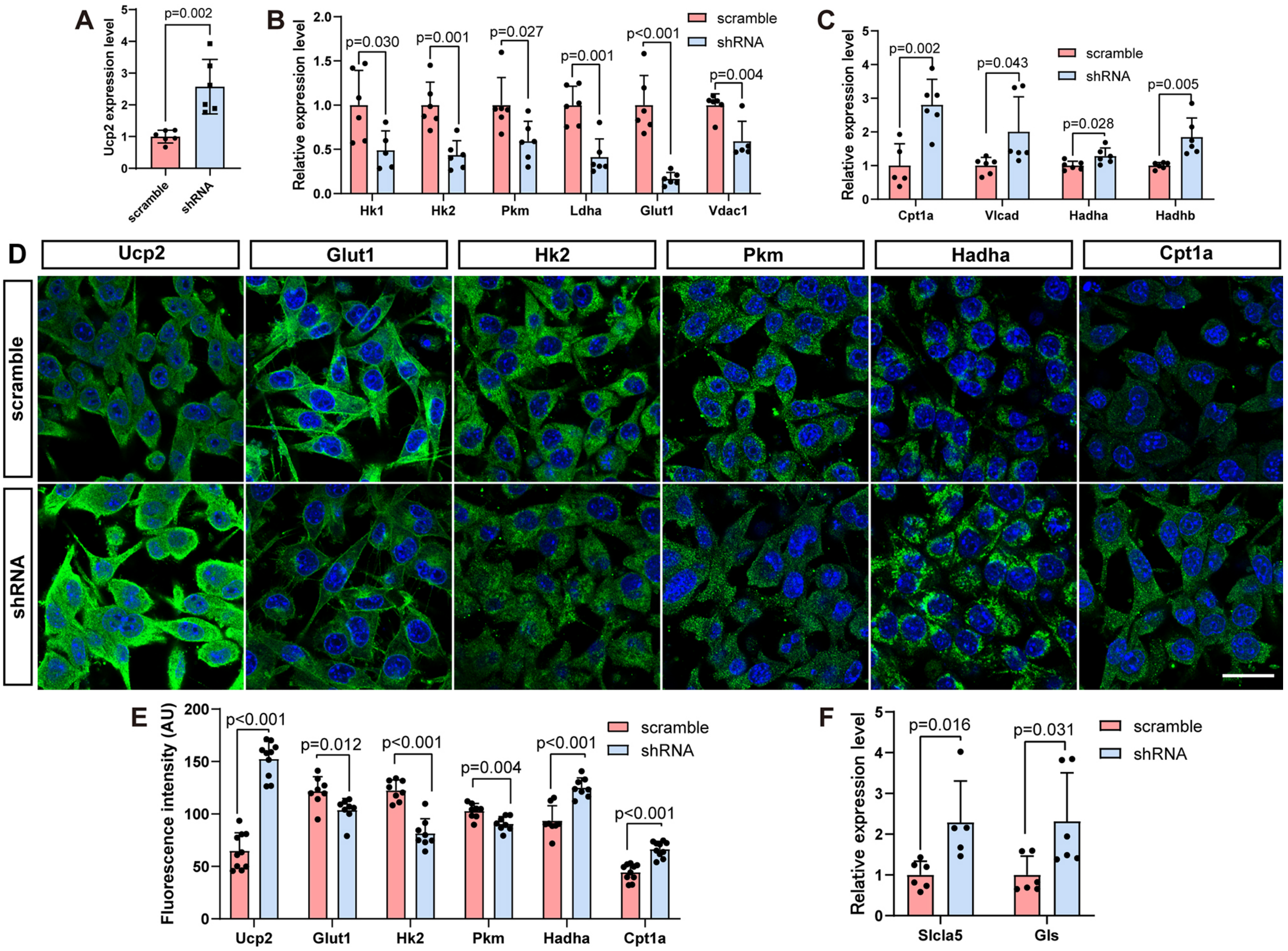

Figure S11. Metabolic alterations caused by *181-Rik* knockdown. (**A-C,F**) qRT-PCR assay of Ucp2 (**A**), glucose metabolism- (**B**), fatty acid metabolism- (**C**) and glutamine metabolism-related genes (**F**) in microglia infected with *181-Rik* shRNA or scramble lentiviruses. Data are presented as mean±SD (n= 5 or 6). (**D,E**) Immunostaining for the indicated proteins (**D**) and quantification of the corresponding immunofluorescence intensity (**E**) in microglia infected with *181-Rik* shRNA or scramble lentiviruses. Data are presented as mean±SD (n= 8-10) in arbitrary unit (AU). Scale bar: 25 μm.

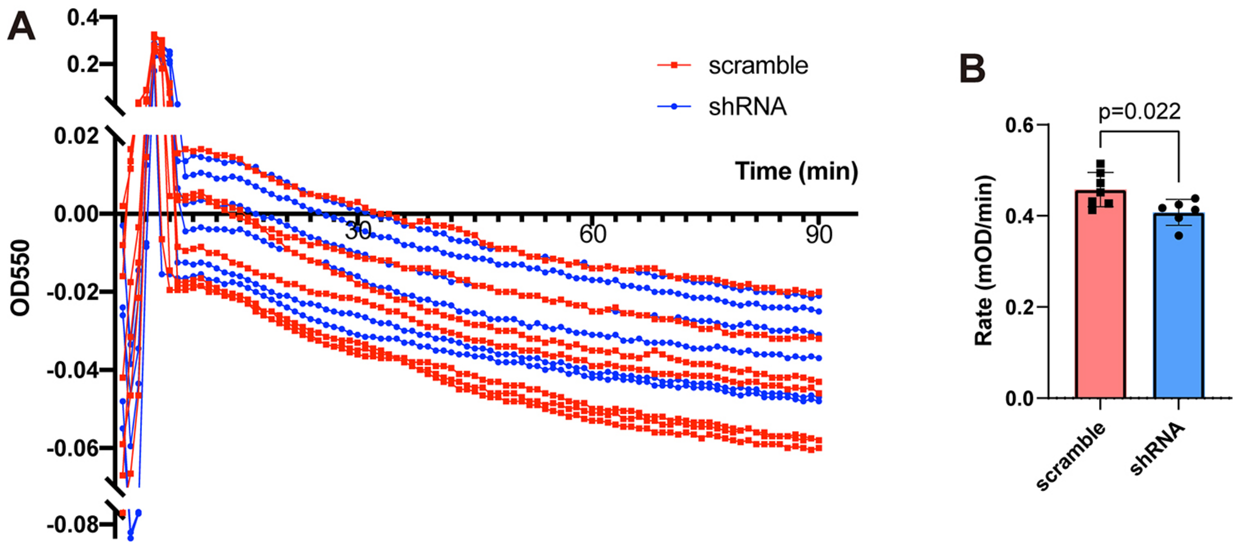

Figure S12. Reduced complex IV (CIV) activity caused by *181-Rik* knockdown. (**A**) Specific absorbance at 550 nm in mitochondria from microglia infected with *181-Rik* shRNA or scramble lentiviruses decreases over time, which reflects the CIV activity. (**B**) Reaction rate of CIV activity of microglia infected with *181-Rik* shRNA or scramble lentiviruses, calculated from (A). Data are presented as mean±SD (n= 6 or 7).

Supplementary Tables

Table S2. shRNA primer sequences

Primer name	Forward	Reverse
181-Rik-shRNA1	CCACTTACTGTGAGCTCTTCTCGAGAAGAGCTCACAGTAAGTGGTTTTT	AAAAACCACTTACTGTGAGCTCTTCTCGAGAAGAGCTCACAGTAAGTGG
181-Rik-shRNA2	GAAATCTTCGCGCTTGGATCTCGAGATCCAAGCGCGAAGATTTCTTTTT	AAAAAGAAATCTTCGCGCTTGGATCTCGAGATCCAAGCGCGAAGATTTC
181-Rik-shRNA3	CCTAAGTCCTTGGGTCTTTCTCGAGAAAGACCCAAGGACTTAGGTTTTT	AAAAACCTAAGTCCTTGGGTCTTTCTCGAGAAAGACCCAAGGACTTAGG
S100a8-shRNA	CACTACTGAGTGTCCTCAGTTCTCGAGAACTGAGGACACTCAGTAGTGTTTTT	AAAAACACTACTGAGTGTCCTCAGTTCTCGAGAACTGAGGACACTCAGTAGTG
scramble	GGAATCTTCGATATATTGGACCTCGAGGTCCAATATATCGAAGATTCCTTTTT	AAAAAGGAATCTTCGATATATTGGACCTCGAGGTCCAATATATCGAAGATTCC

Table S3. qRT-PCR primer sequences

Gene	Forward	Reverse
181-Rik	TCTCCCCGTTCCCGTATCTT	TGCTAGACAGTGAGTCCCCA
Gsdmd	TTCCAGTGCCTCCATGAATGT	GCTGTGGACCTCAGTGATCT
IL-1β	TTCAGGCAGGCAGTATCACTC	GAAGGTCCACGGGAAAGACAC
Asc	CTGGAGTCGTATGGCTTGGA	GTCCACAAAGTGTCCTGTTCTG
Casp8	TGCTTGGACTACATCCCACAC	GTTGCAGTCTAGGAAGTTGACC
Casp1	ACAAGGCACGGGACCTATG	TCCCAGTCAGTCCTGGAAATG
IL-18	GACTCTTGCGTCAACTTCAAGG	CAGGCTGTCTTTTGTCAACGA
Tlr4	TCCCTGCATAGAGGTAGTTCC	TCAAGGGGTTGAAGCTCAGA
Myd88	AGGACAAACGCCGGAACTTTT	GCCGATAGTCTGTCTGTTCTAGT
β-actin	CAACGGCTCCGGCATGTGC	CTCTTGCTCTGGGCCTCG
Rbpms	TAGTGAGCAAACGGACTGAG	TGTTGTGAAGTCTTGAAGGG
Thy1	TTTGTGAGCTTCAAGTCTCG	CACTTGACCAGCTTGTCTC
Brn3a	CCTCGTCTGAGAAGATCGCC	CAGGGACCCCTCAGCTCC
Brn3b	TGGACATCGTCTCCCAGAGTA	GTGTTCATGGTGTGGTAAGTGG
Brn3c	GTCTCCCACGGCAAGAATCA	GTGCTCAAGTAAGTCGCCCT
GFP	GACGACGGCAACTACAAGAC	CGGCCATGATATAGACGTTG

Casp4/11	AGCGTTGGGTTTTTGTAGATGC	CCTTGTGAACTCTTCAGGGGA
Casp3	CTTCATCATTCAGGCCTGCCG	CAGCCTCCACCGGTATCTTC
Casp6	AAAAGTAGGGAAGTGTTCGATCC	CGAGTCAGGTTGTCTCTGTCTG
Casp7	TTCTTCCAGCGAAGACGGAG	TTCCGGACGTCCATACCTGT
Aif	TGATTGCAACGGGAGGCAC	GGATCACTTCTATGCCCGAGG
Trem2	TGAAGAAGCGGAATGGGAGC	ACAGGATGAAACCTGCCTGGAG
C1qa	AAACTTGGCAGTGTCCTGGT	GTCTCCATGGTGTCCCTGC
C1qb	ATAAAGGGGGAGAAAGGGCTC	TAGTTCTCGTTCGCGTTGGT
C1qc	AGTCCCTTACACCCTCAGGA	GGCTGGGATTCCTGGCTC
Nos2	GGTGAAGGGACTGAGCTGTT	ACGTTCTCCGTTCTCTTGCAG
Grn	ATGTGGGTCCTGATGAGCTG	GCTCGTTATTCTAGGCCATGTG
Lcn2	TCTGTCCCCACCGACCAATG	GGGGAGTGCTGGCCAAATAA
Casp12	AGACAGAGTTAATGCAGTTTGCT	TTCACCCCACAGATTCCTTCC
Tnfrsf1a	CTTCAGCACCCCAGGCTTTA	TCGCAAGGTCTGCATTGTCA
Tnfrsf1b	CTAAGTGTCCTCCTGGCCAAT	TGGGTTTTCAAGGCGCAGTA
Gapdh	TCCCACTCTTCCACCTTCGATGC	GGGTCTGGGATGGAAATTGTGAGG
S100a8	AAATCACCATGCCCTCTACAAG	CCCACTTTTATCACCATCGCAA
S100a9	ATACTCTAGGAAGGAAGGACACC	TCCATGATGTCATTTATGAGGGC
Opa1	CGACTTTGCCGAGGATAGCTT	CGTTGTGAACACACTGCTCTTG
Mfn1	ATGGCAGAAACGGTATCTCCA	CTCGGATGCTATTCGATCAAGTT
Mff	ATGCCAGTGTGATAATGCAAGT	CTCGGCTCTCTTCGCTTTG
Ucp2	ATGGTTGGTTTCAAGGCCACA	CGGTATCCAGAGGGAAAGTGAT
Cpt1a	CTCCGCCTGAGCCATGAAG	CACCAGTGATGATGCCATTCT
Vlcad	CTACTGTGCTTCAGGGACAAC	CAAAGGACTTCGATTCTGCCC
Hadha	TGCATTTGCCGCAGCTTTAC	GTTGGCCCAGATTTCGTTCA
Hadhb	ACTACATCAAAATGGGCTCTCAG	AGCAGAAATGGAATGCGGACC
Slc1a5	CATCAACGACTCTGTTGTAGACC	CGCTGGATACAGGATTGCGG
Gls	TTCGCCCTCGGAGATCCTAC	CCAAGCTAGGTAACAGACCCT
Hk1	AGGGCGCATTACTCCAGAG	CCCTGTGGGTGTCTTGTGTG
Hk2	TGATCGCCTGCTTATTCACGG	AACCGCCTAGAAATCTCCAGA
Pkm2	GCCGCCTGGACATTGACTC	CCATGAGAGAAATTCAGCCGAG
Ldha	TGTCTCCAGCAAAGACTACTGT	GACTGTACTTGACAATGTTGGGA
Glut1	CAGTTCGGCTATAACACTGGTG	GCCCCCGACAGAGAAGATG
Vdac1	CCCACATACGCCGATCTTGG	GTGGTTTCCGTGTTGGCAGA

Table S4. Primary antibodies for immunostaining

Name	Company	Cat. No	Host	Dilution
Iba1	Abcam	ab178847	Rb	1:1000
Iba1	Wako	019-19741	Rb	1:1000
Flag	Sigma	F1804	Ms	1:1000
Tomm20	Abcam	ab186734	Rb	1:1000
Stmp1	Novus Biologicals	NBP2-56550	Rb	1:100

Gsdmd	Santa Cruz	sc-393656	Ms	1:200
Casp1	Santa Cruz	sc-56036	Ms	1:200
Ki67	BD Biosciences	550609	Ms	1:50
CD206	R&D Systems	AF2535	Gt	1:1000
CD16/32	BD Biosciences	553141	Rt	1:1000
CD11b	Abcam	ab8878	Rt	1:1000
Rbpms	PhosphoSolutions	1830-	Rb	1:1000
S100a8	Invitrogen	PA5-47439	Gt	1:30
S100a9	Abcam	ab242945	Rb	1:500
GFP	MBL	598	Rb	1:1000
Chx10	Santa Cruz	sc-21690	Gt	1:1000
Sox9	Millipore	AB5535	Rb	1:1000
Tfap2a	Abcam	ab108311	Rb	1:1000
Recoverin	Millipore	AB5585	Rb	1:10000
Gfap	Dako	Z0334	Rb	1:1000
Mfn1	Abcam	ab104274	Rb	1:1000
Mff	CST	84580	Rb	1:500
Ucp2	Novus Biologicals	NB100-59742	Gt	1::50
Glut1	abcam	ab115730	Rb	1:500
Hk2	abcam	ab209847	Rb	1:500
Pkm	abcam	ab150377	Rb	1:500
Hadha	abcam	ab203114	Rb	1:500
Cpt1a	abcam	ab128568	Ms	1:500
Tmem119	CST	90840	Rb	1:200
Cox4	abcam	ab33985	Ms	1:1000

Table S5. Primary antibodies for Western blotting

Name	Company	Cat. No	Host	Dilution
β-tubulin	Abcam	ab179513	Rb	1:2000
Nlrp3	Abcam	ab214185	Rb	1:1000
Tlr4	CST	14358	Rb	1:1000
Asc	CST	67824	Rb	1:1000
Gsdmd	Abcam	ab219800	Rb	1:1000
Casp1	Abcam	ab179515	Rb	1:1000
IL-1β	Abcam	ab234437	Rb	1:1000
β-actin	CST	4970	Rb	1:2000
Flag	Sigma	F1804	Ms	1:1000
GFP	MBL	598	Rb	1:1000
TNF-α	Abcam	ab215188	Rb	1:1000
S100a9	Abcam	ab242945	Rb	1:1000
S100a8	Invitrogen	PA5-47439	Gt	1:100
Casp8	Abcam	ab108333	Rb	1:1000

Casp7	Abcam	ab181579	Rb	1:1000
Stmp1	Novus Biologicals	NBP2-56550	Rb	1:100
Cpt1a	Abcam	ab128568	Ms	1:1000
Acsl4	Abcam	ab155282	Rb	1:1000
Mfn1	Abcam	ab104274	Rb	1:1000
Ucp2	Abcam	ab203244	Rb	1:1000
Opa1	Abcam	ab42364	Rb	1:1000
Uqcrfs1	Abcam	ab14746	Ms	1:1000
Histone H3K27me3	Active Motif	39157	Rb	1:1000
Stmp1	Abmart	Ordered	Rb	1:100

Table S6. Secondary antibodies

For immunostaining	Company	Dilution
Donkey anti-rat Alexa488 IgG	Invitrogen	1:1000
Donkey anti-rat Alexa 594 IgG	Invitrogen	1:1000
Donkey anti-rat Alexa 647 IgG	Invitrogen	1:1000
Donkey anti-mouse Alexa488 IgG	Invitrogen	1:1000
Donkey anti-mouse Alexa 555 IgG	Invitrogen	1:1000
Donkey anti-mouse Alexa 594 IgG	Invitrogen	1:1000
Donkey anti-mouse Alexa 647 IgG	Invitrogen	1:1000
Donkey anti-rabbit Alexa488 IgG	Invitrogen	1:1000
Donkey anti-rabbit Alexa 555 IgG	Invitrogen	1:1000
Donkey anti-rabbit Alexa 594 IgG	Invitrogen	1:1000
Donkey anti-rabbit Alexa 647 IgG	Invitrogen	1:1000
Donkey anti-goat Alexa488 IgG	Invitrogen	1:1000
Donkey anti-goat Alexa 594 IgG	Invitrogen	1:1000
**For Western blotting**	**Company**	**Dilution**
Anti-rabbit IgG, HRP-linked Antibody	CST	1:2000
Anti-mouse IgG, HRP-linked Antibody	CST	1:2000
Anti-goat IgG, HRP-linked Antibody	CST	1:2000
