# Supplementary material for: A lncRNA-encoded mitochondrial micropeptide exacerbates microglia-mediated neuroinflammation in retinal ischemia/reperfusion injury
Source: Cell Death Dis. 2023 Feb 15;14(2):126. doi: 10.1038/s41419-023-05617-2 (PMC9932084; doi:10.1038/s41419-023-05617-2)
Supplement: Supplementary file 3 — Full and Uncropped Western Blots [file 41419_2023_5617_MOESM3_ESM.pdf]

Original Western Blot

A lncRNA-encoded mitochondrial micropeptide exacerbates microglia-mediated neuroinflammation in retinal ischemia/reperfusion injury

Figure 1

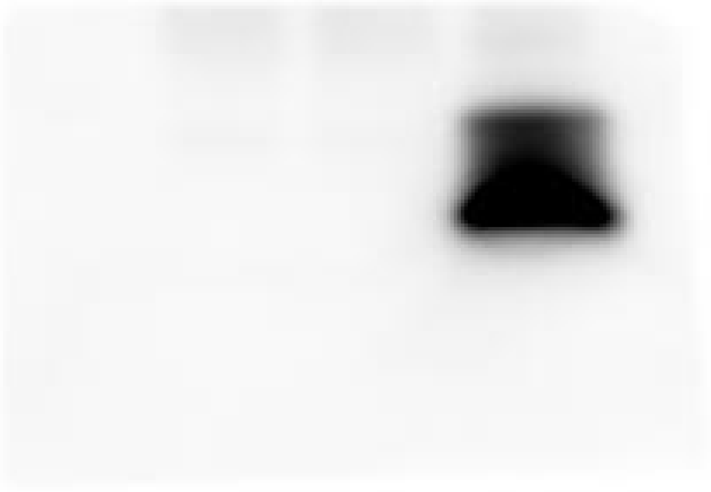

Flag

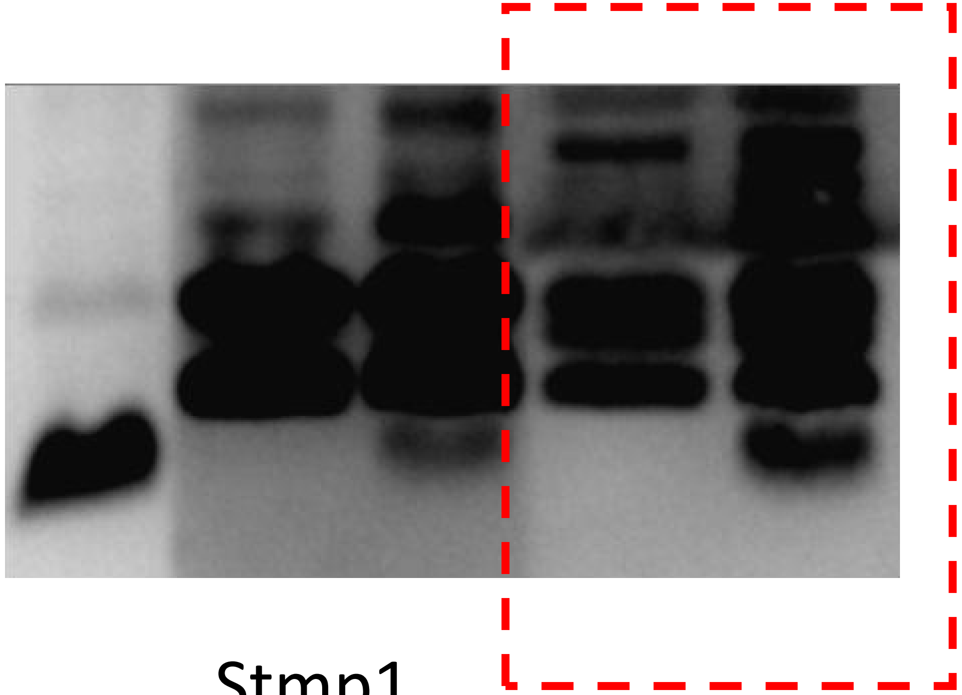

Stmp1

Stmp1

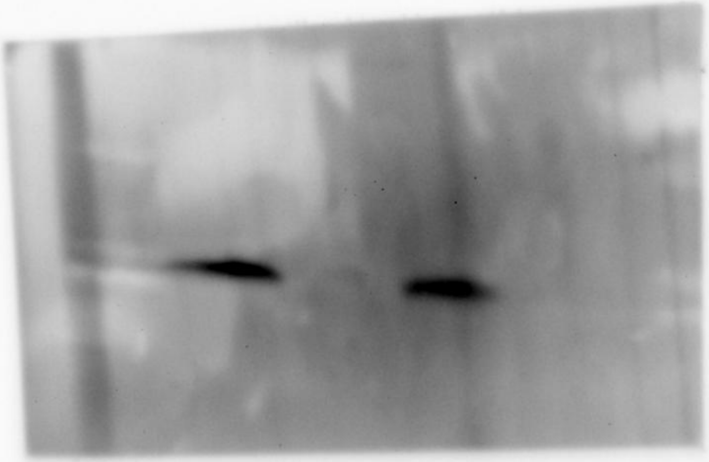

Mfn1

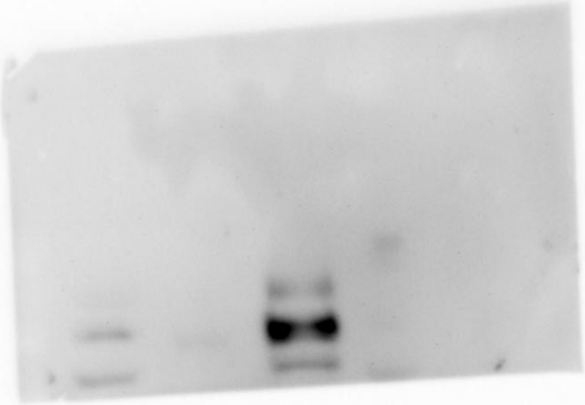

β-tubulin

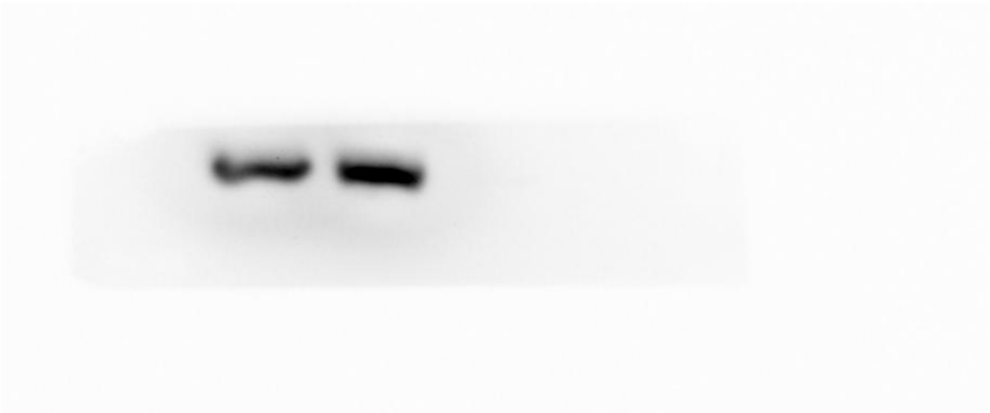

Histone
H3K27me3

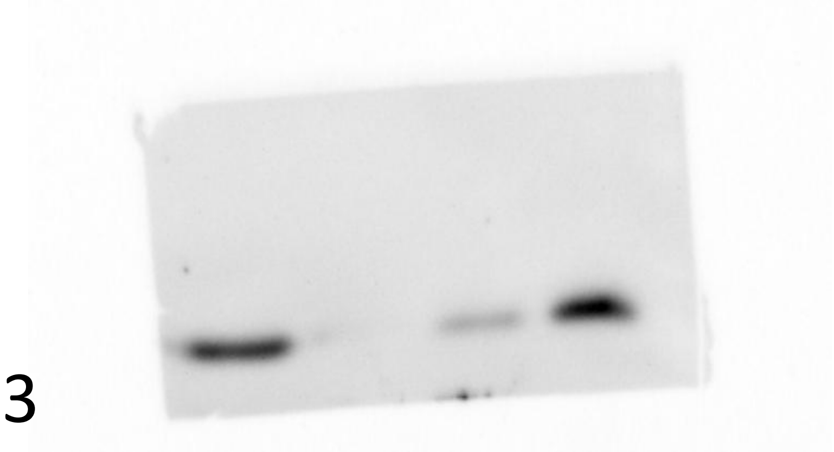

Figure 4

Stmp1

Nlrp3

Tlr4

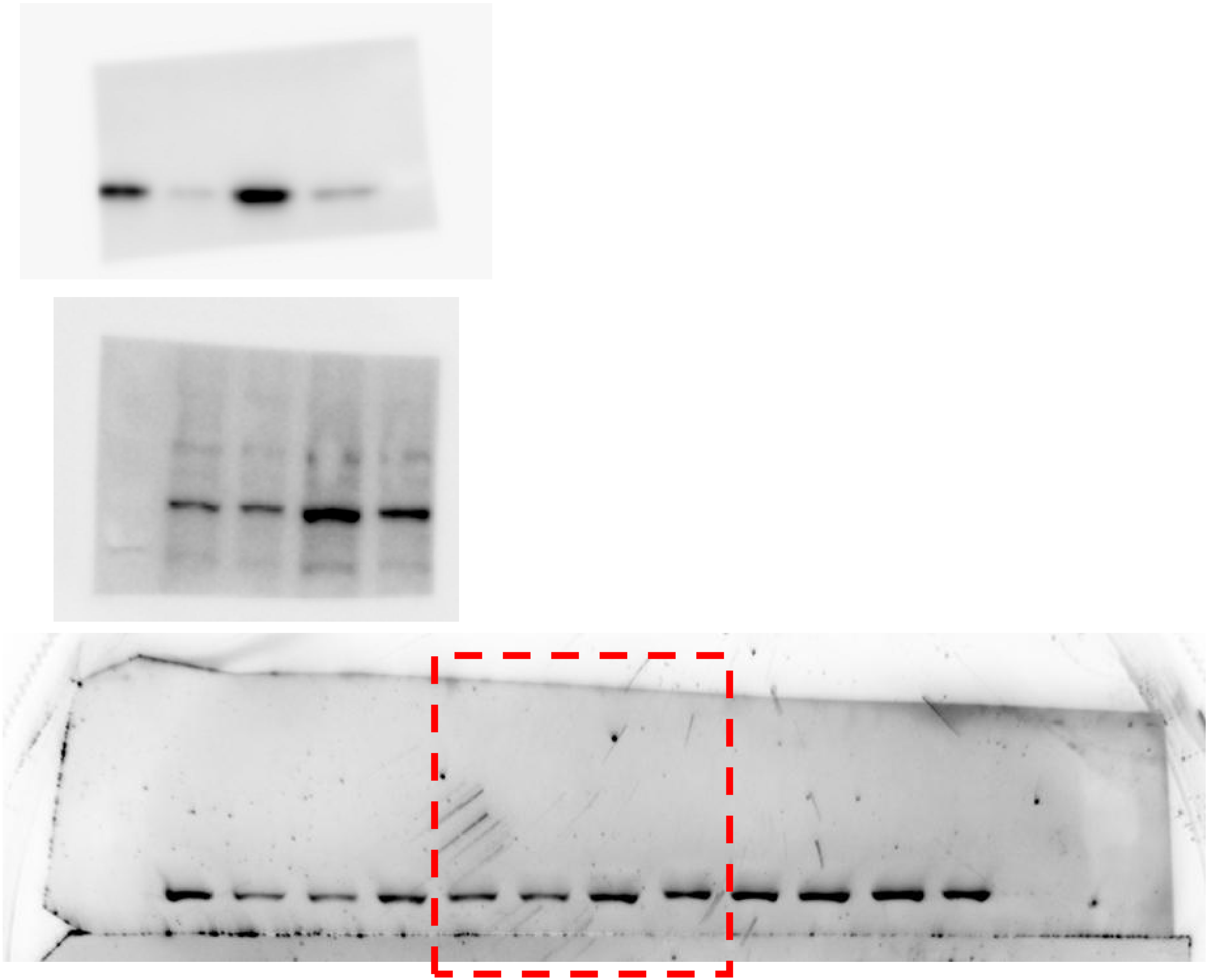

Asc

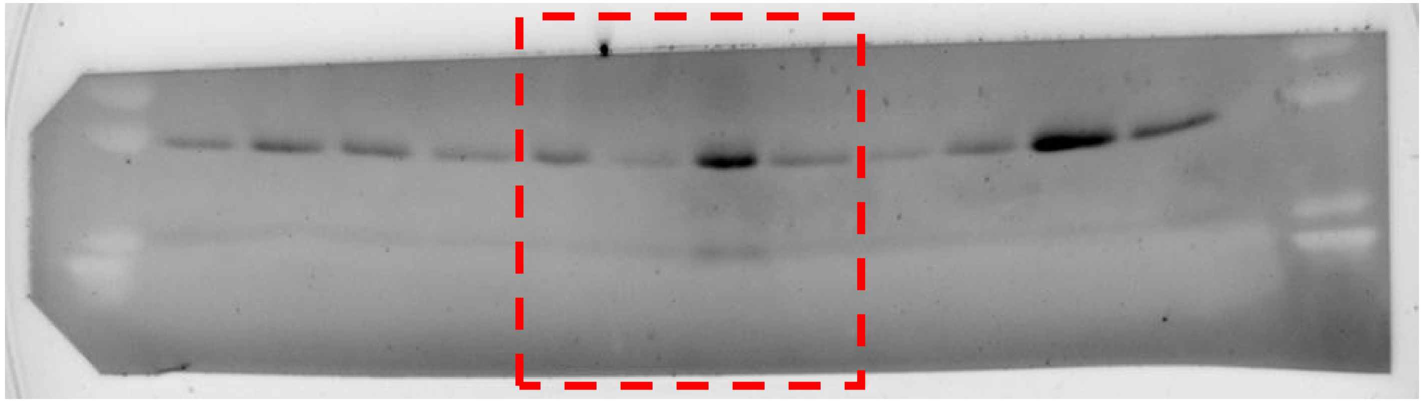

Gsdmd

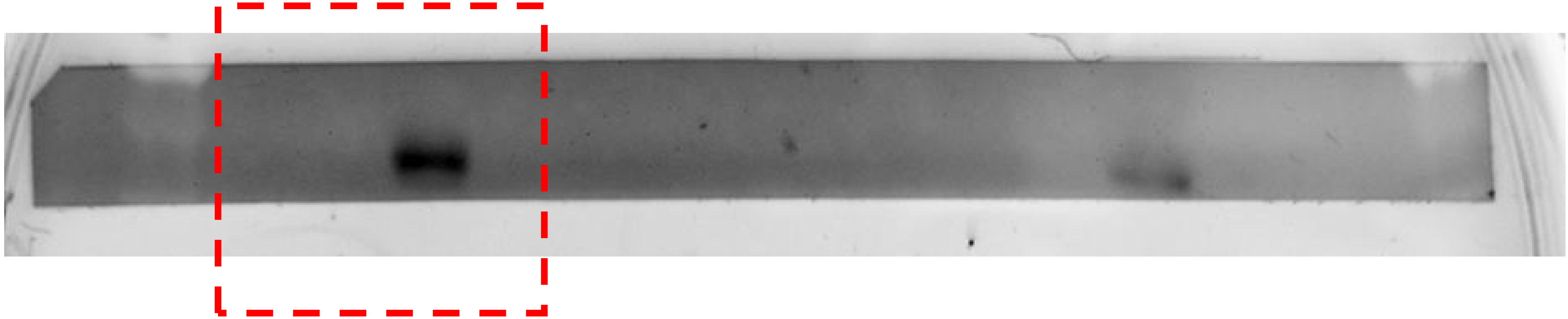

Casp1

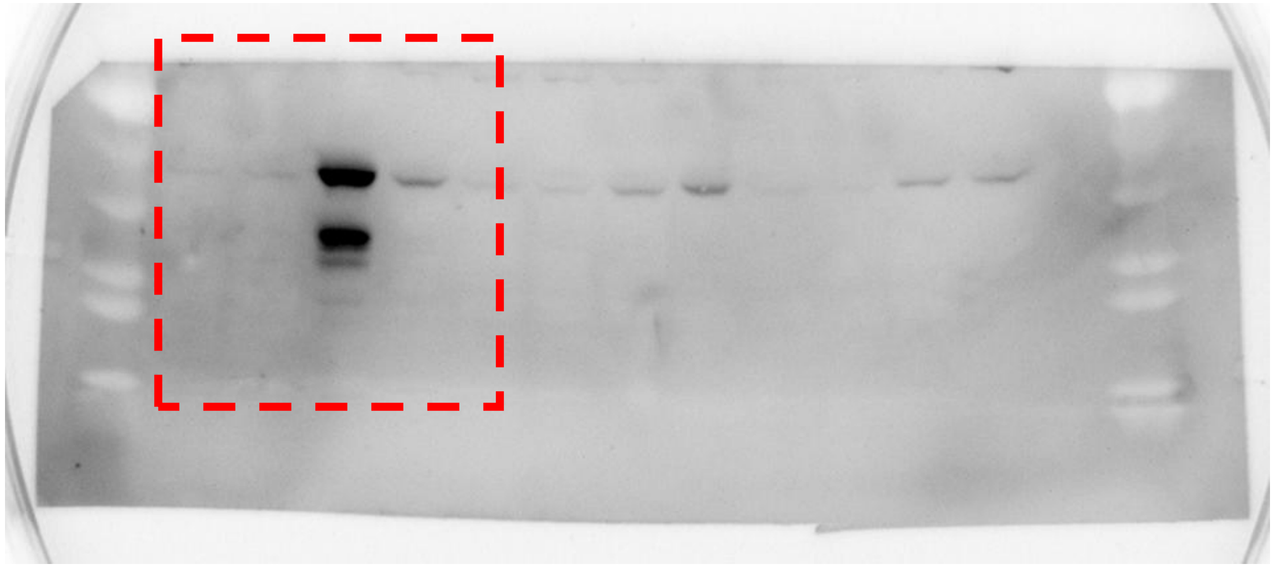

IL-1β

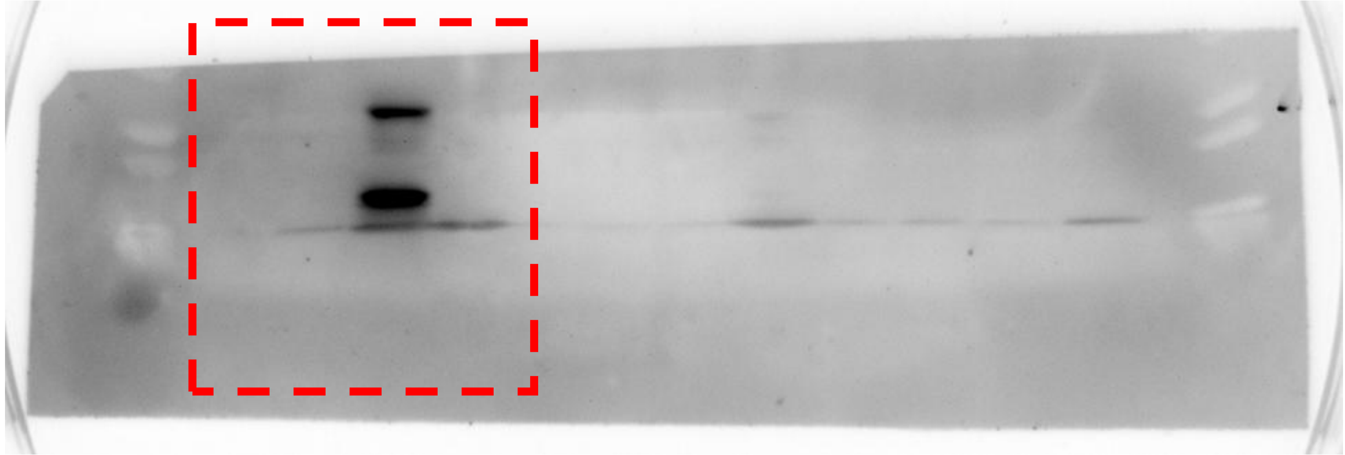

Casp8

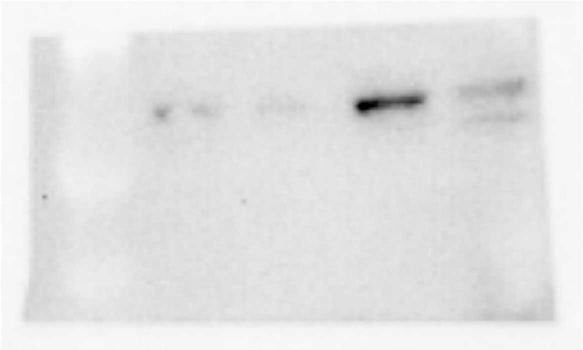

TNF-α

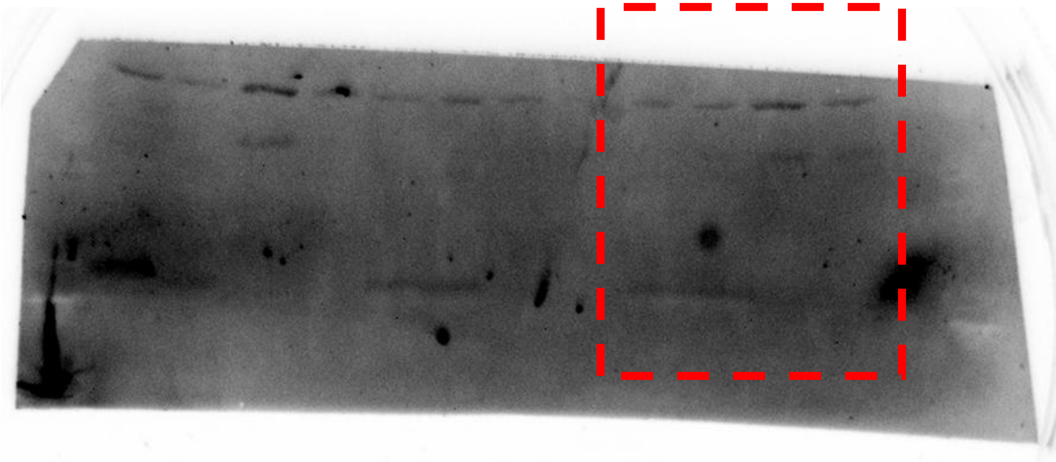

Casp7

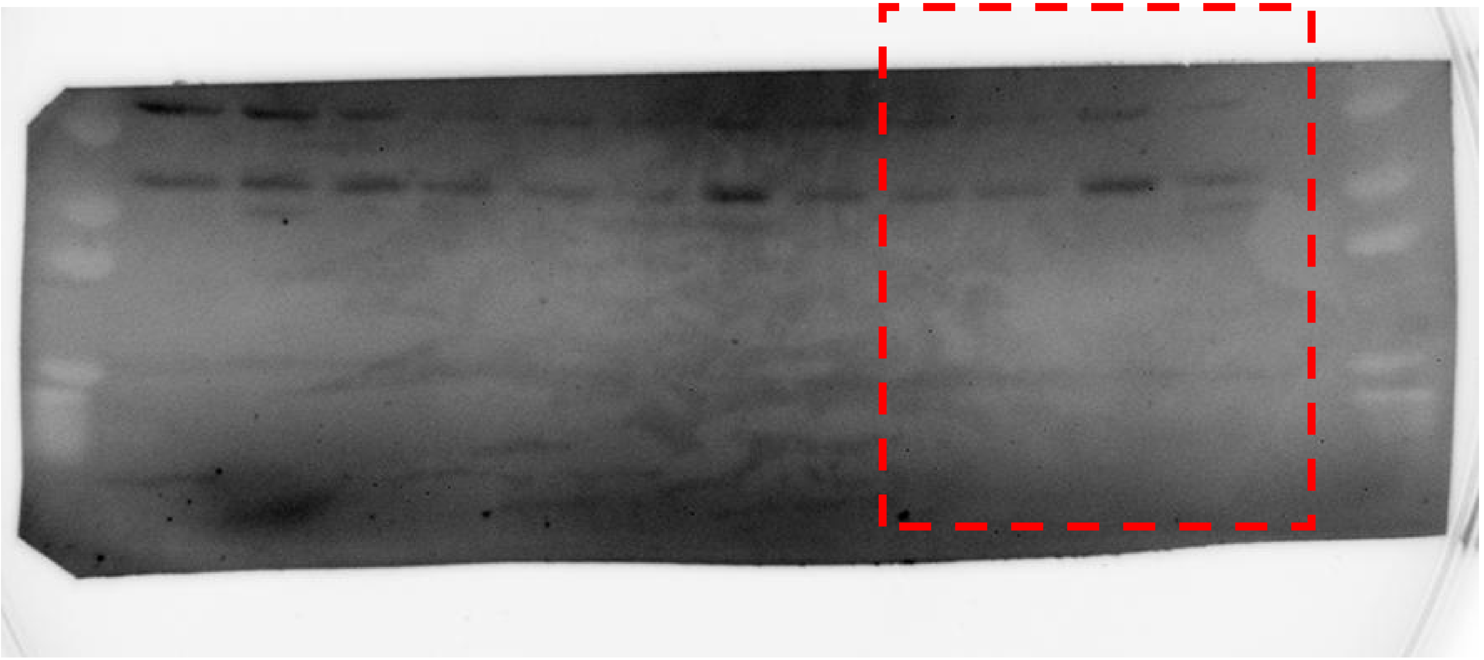

β-tubulin

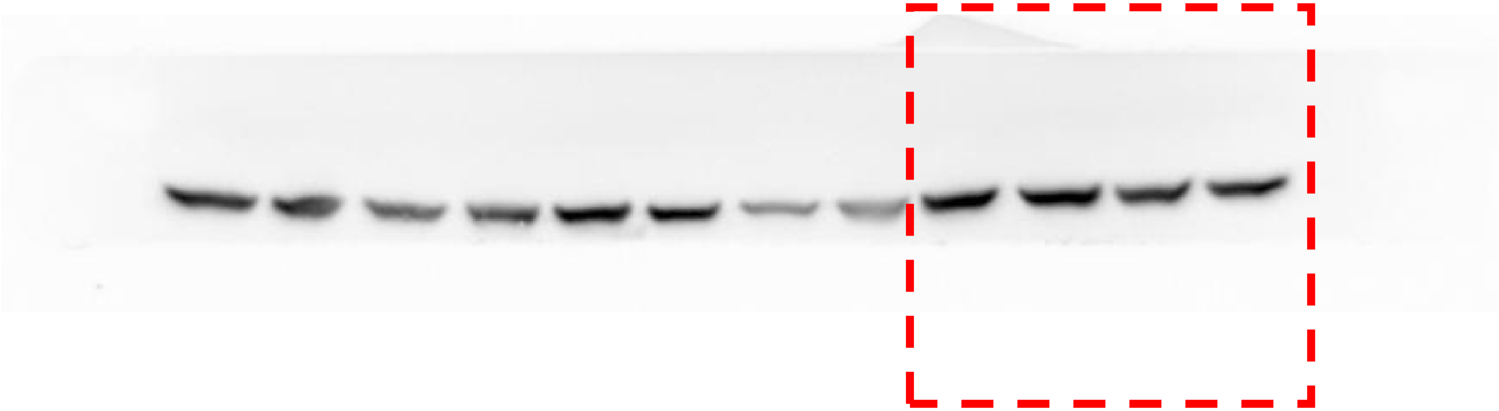

Figure 7

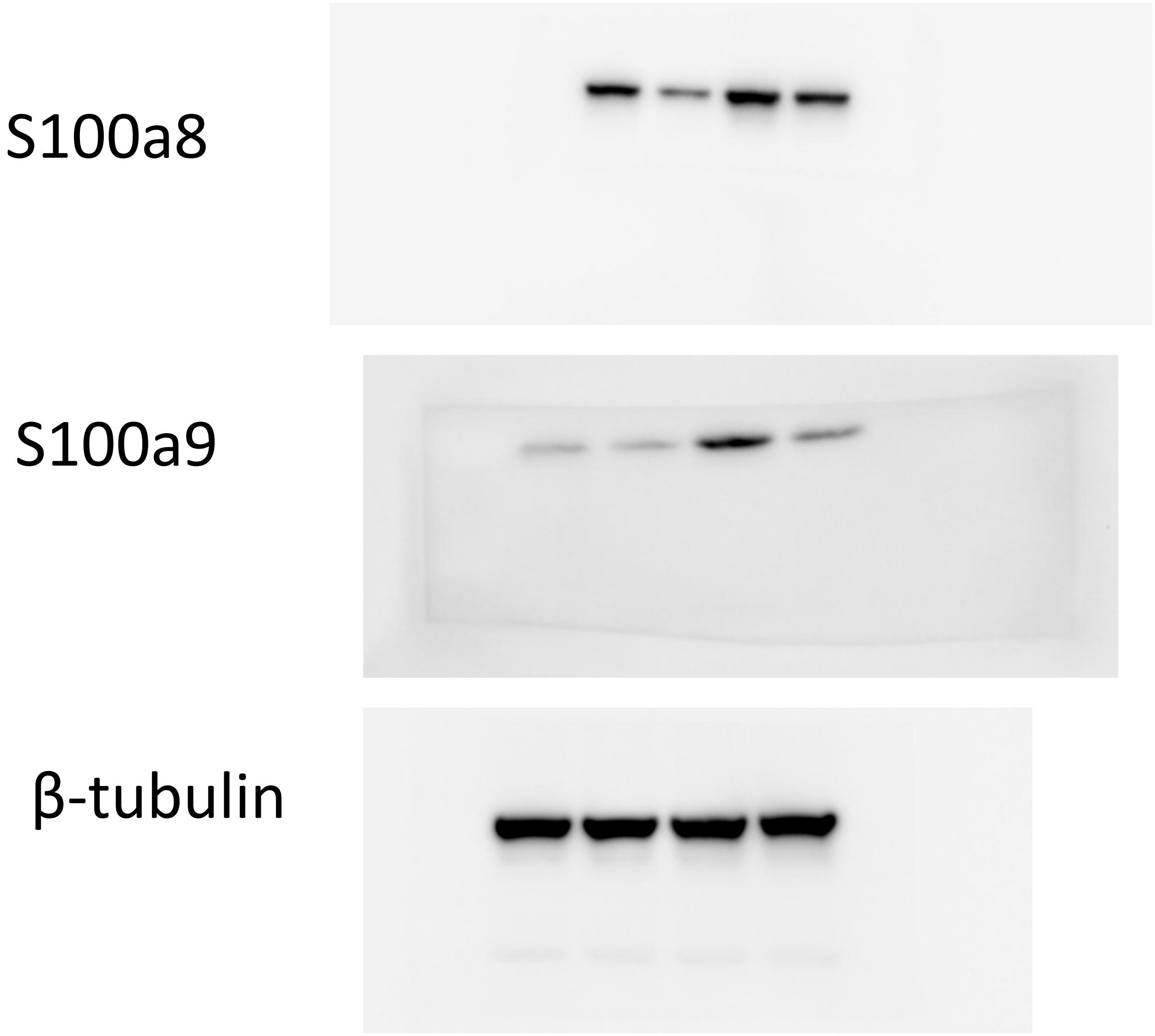

Figure 8

Stmp1

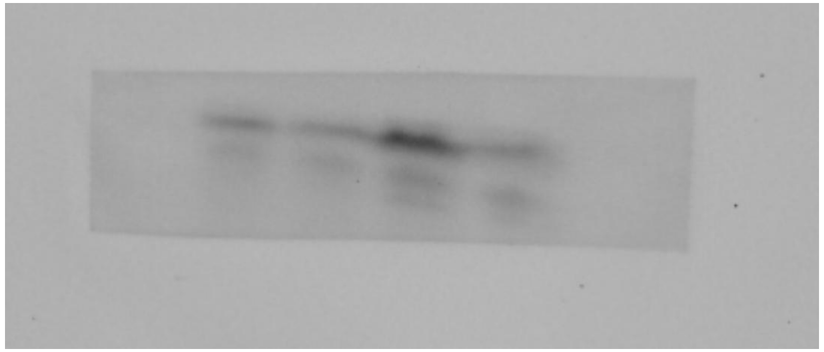

Nlrp3

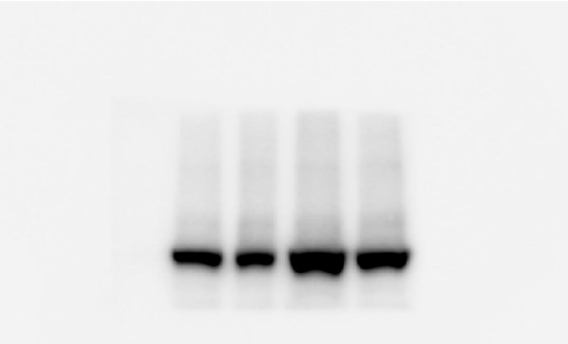

Nlrp6

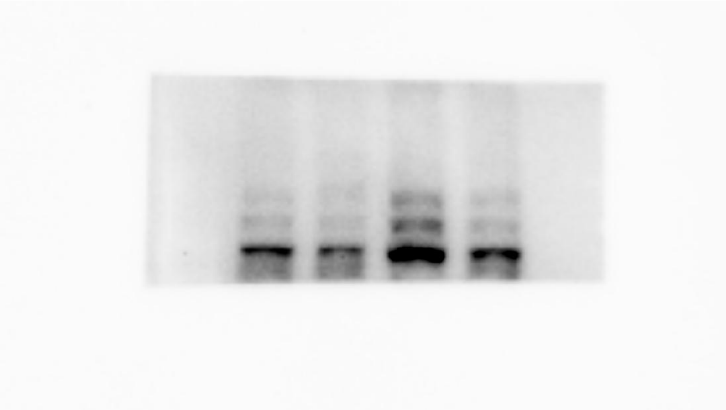

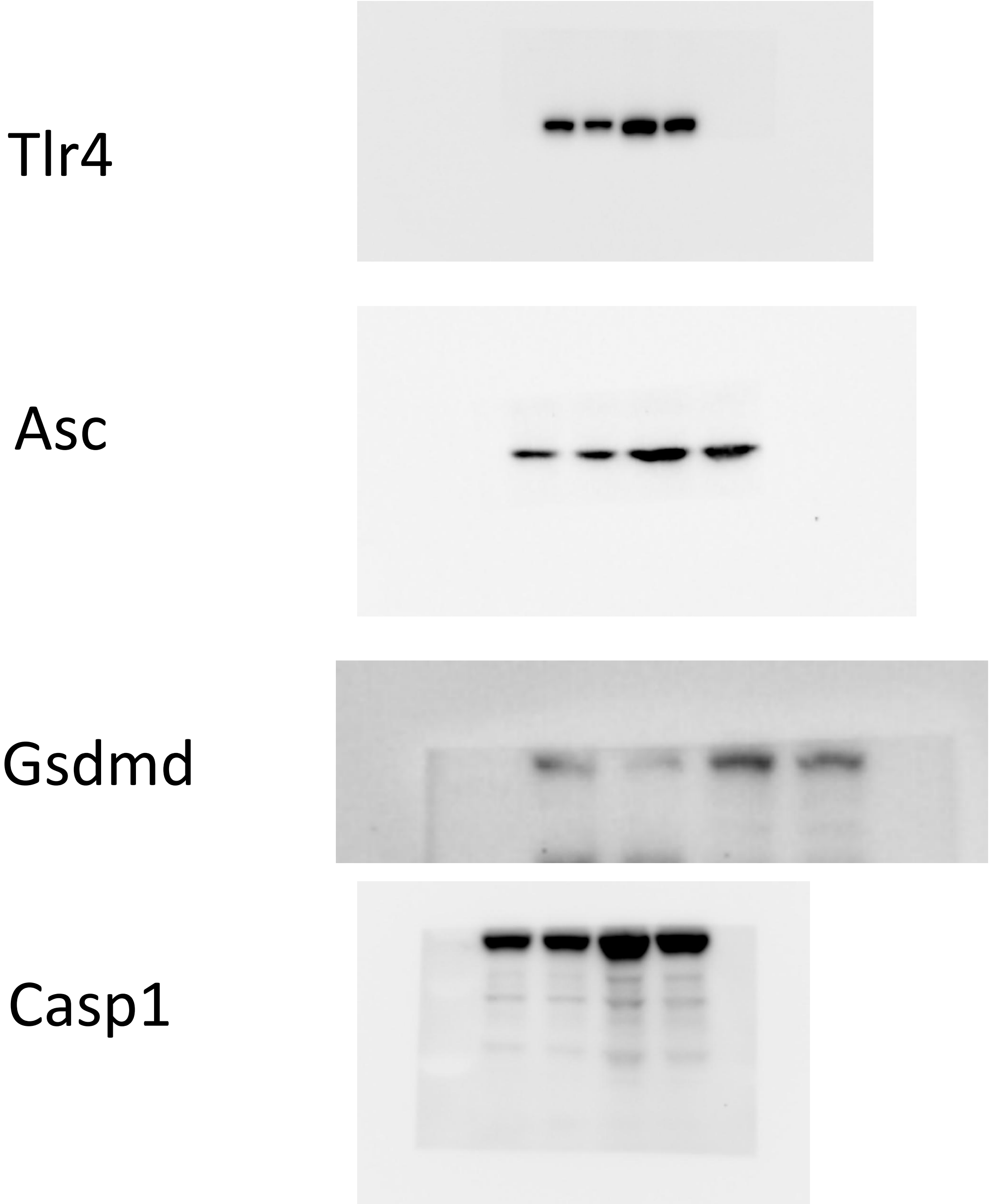
Tlr4
Asc
Gsdmd
Casp1

IL-1β

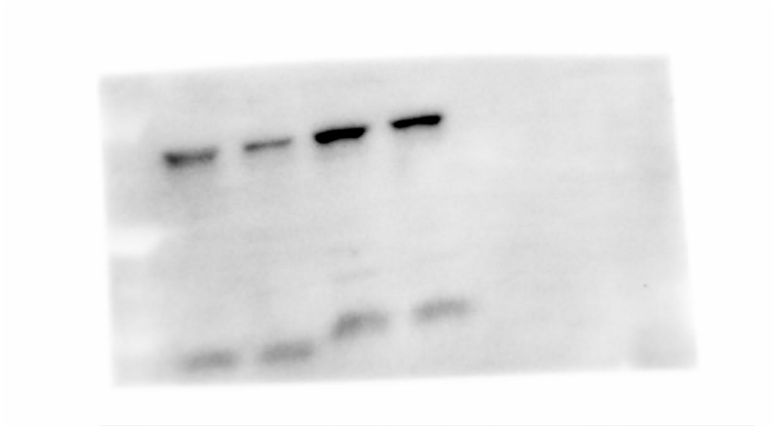

Casp8

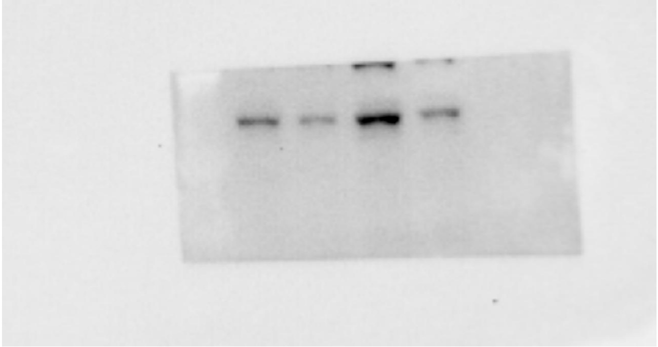

β-tubulin

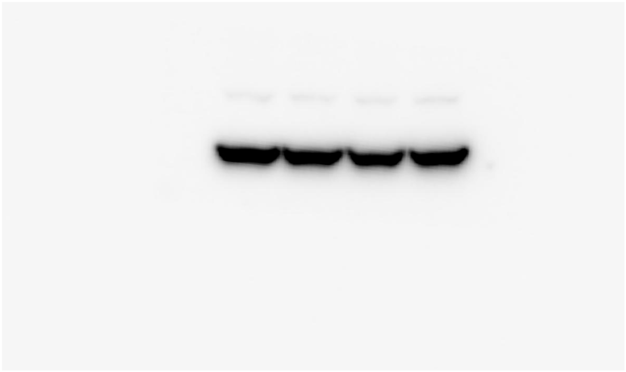

Figure S2

Cpt1a

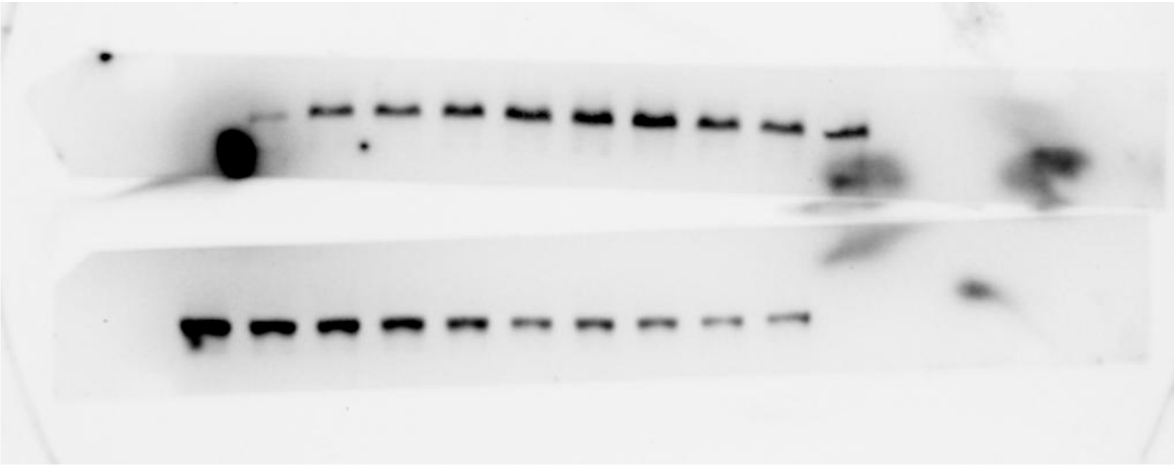

Acsl4

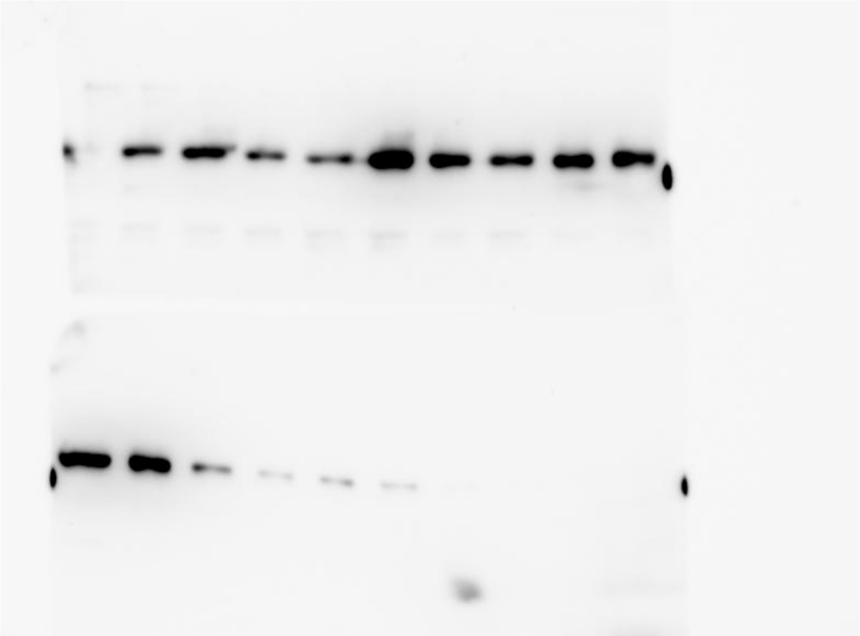

Mitofusion1

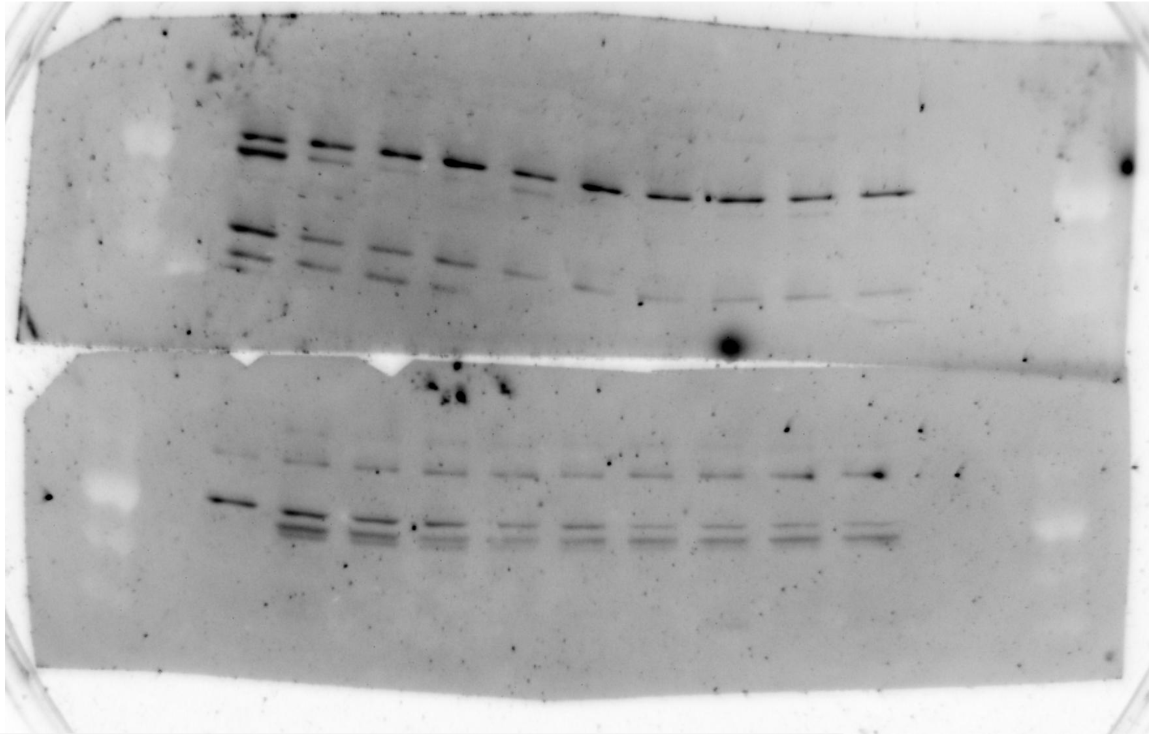

Ucp2

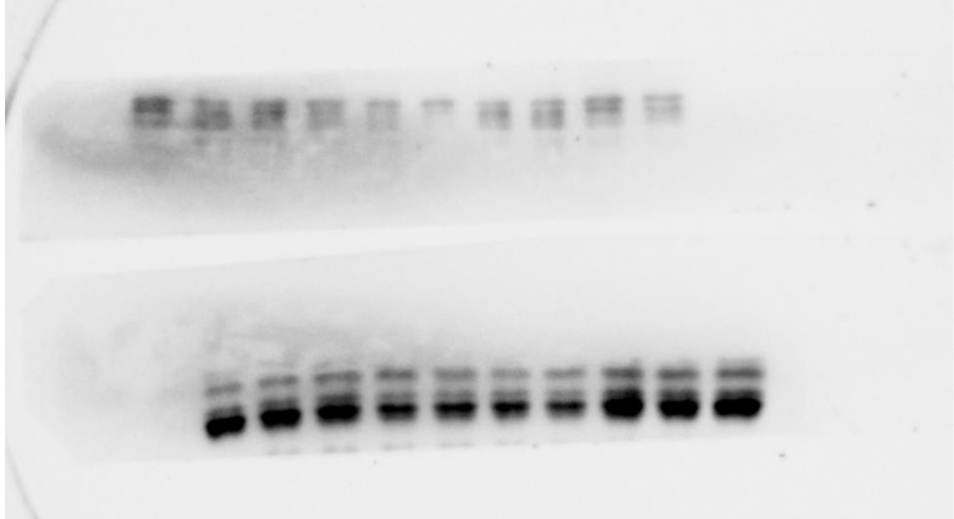

Opa1

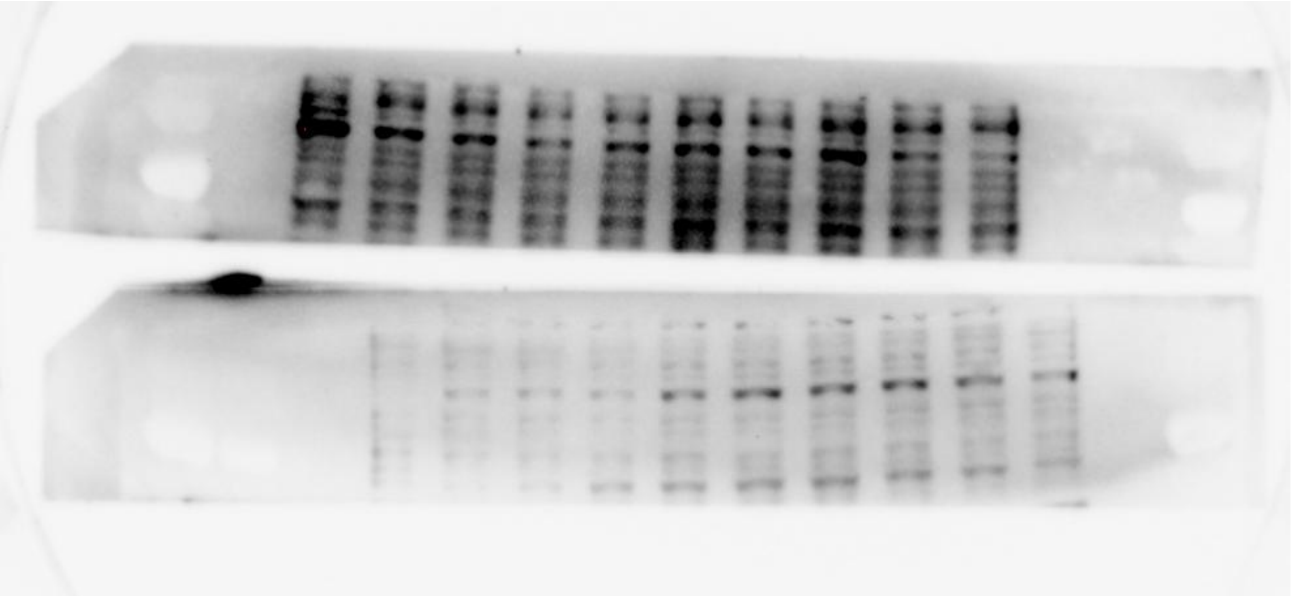

Uqcrfs1

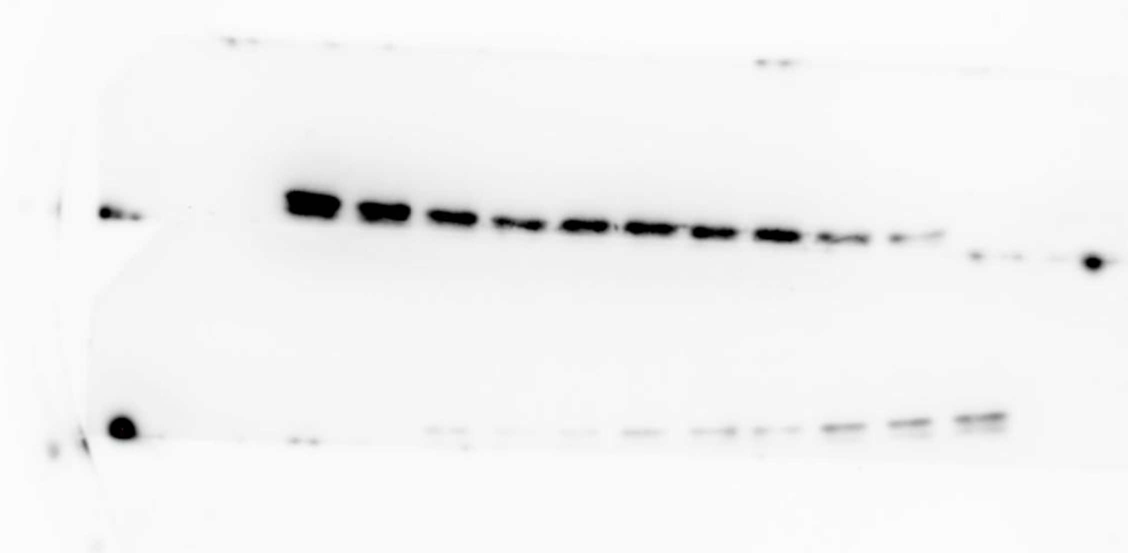

Flag

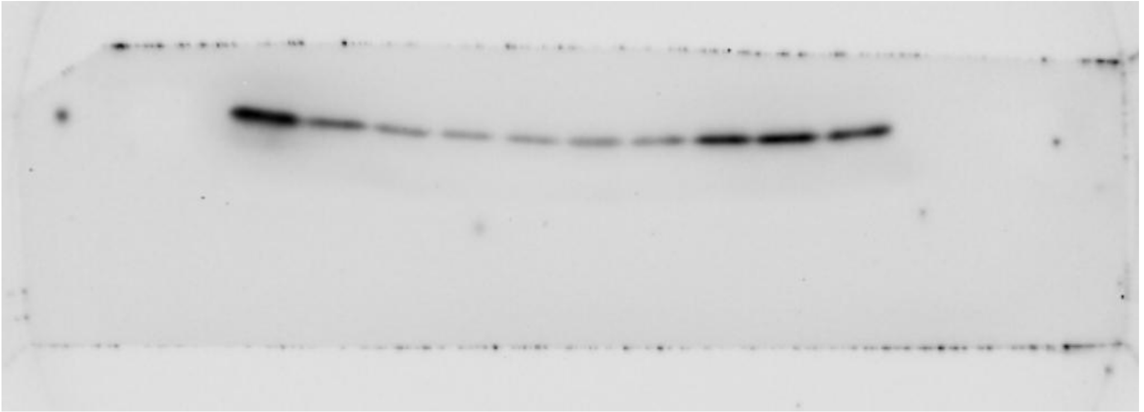

Flag

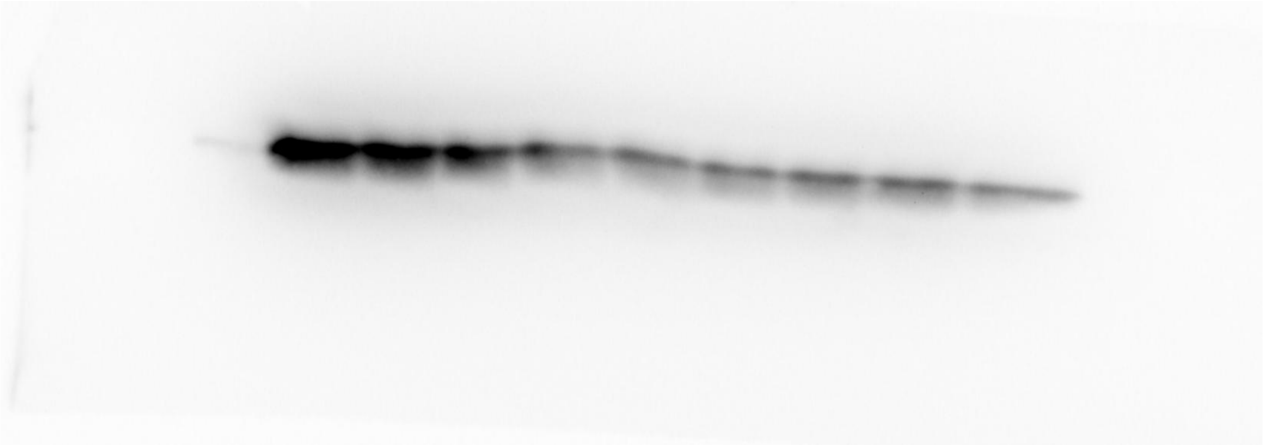

Figure S10

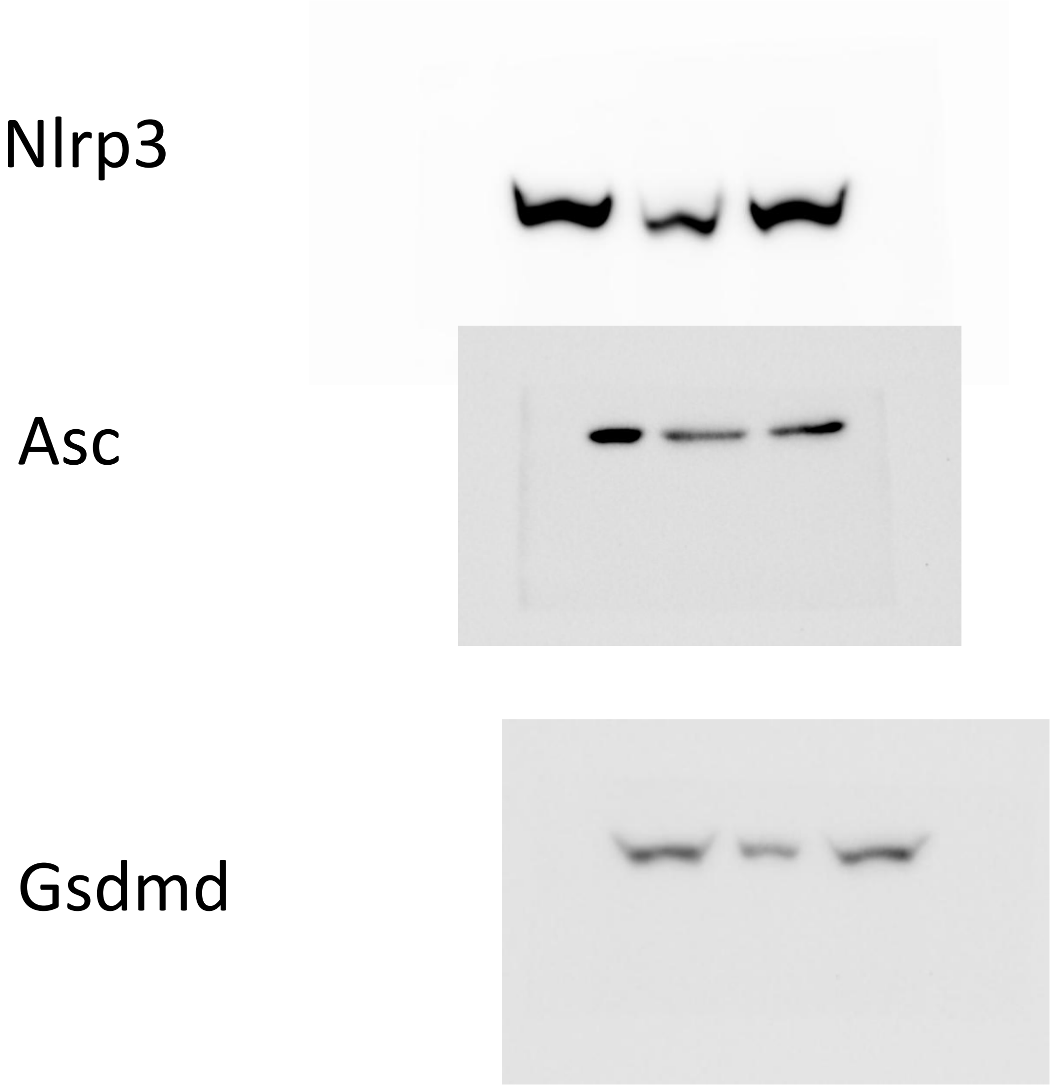

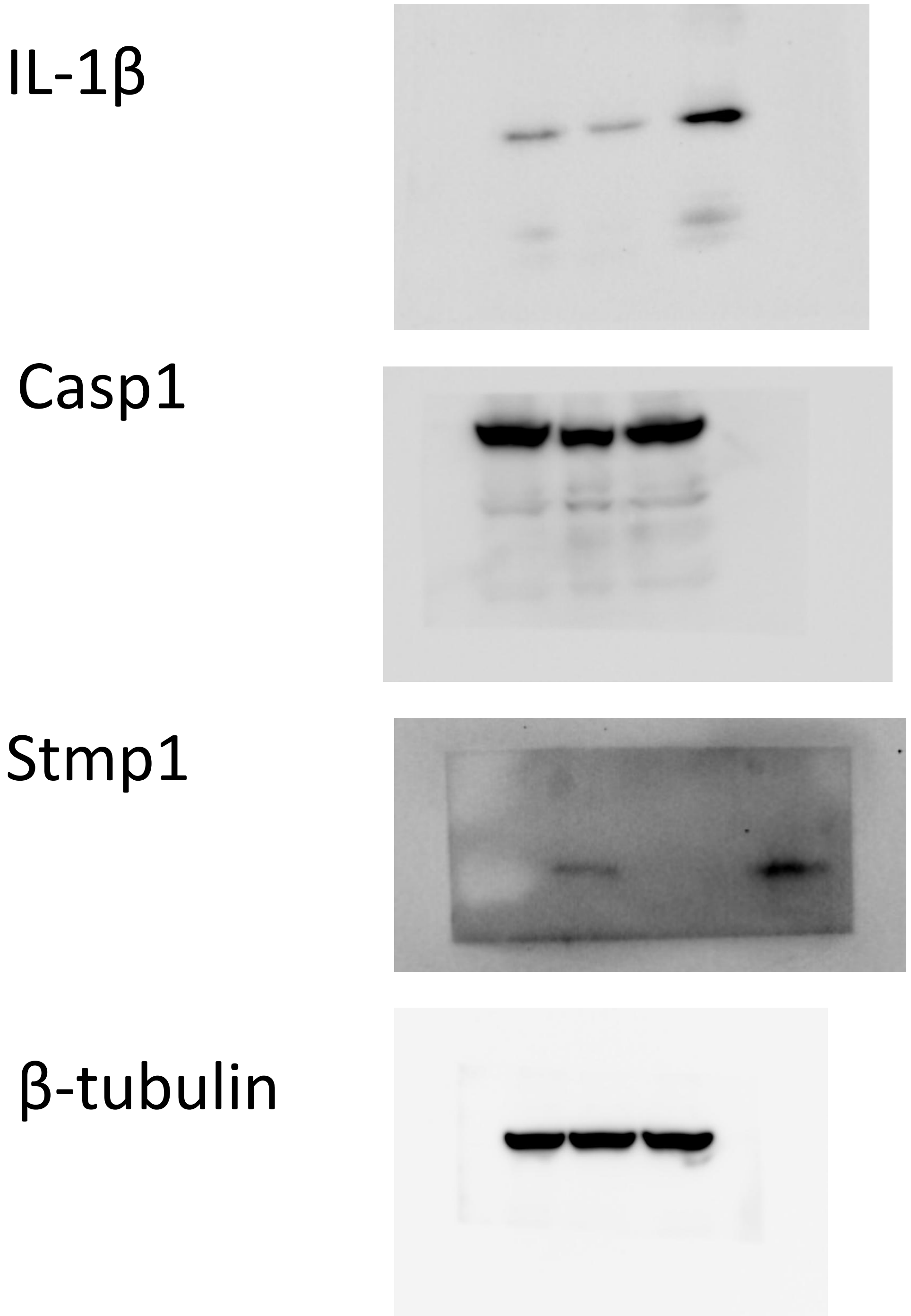
IL-1β
Casp1
Stmp1
β-tubulin
